# Metabolic Responses to the Zinc Stress in the Roots and Leaves of *Amaranthus caudatus*: The Proteomics View

**DOI:** 10.3390/plants14213315

**Published:** 2025-10-30

**Authors:** Anastasia Gurina, Tatiana Bilova, Daria Gorbach, Alena Soboleva, Nataliia Stepanova, Olga Babich, Christian Ihling, Anastasia Kamionskaya, Natalia Osmolovskaya, Andrej Frolov

**Affiliations:** 1 Department of Plant Physiology and Biochemistry, St. Petersburg State University, 199034 St. Petersburg, Russia; anastasia.gurina@list.ru (A.G.); n.osmolovskaya@spbu.ru (N.O.); 2 Laboratory of Analytical Biochemistry and Biotechnology, K.A. Timiryazev Institute of Plant Physiology, Russian Academy of Science, 127276 Moscow, Russia; daria.gorba4@yandex.ru (D.G.); oriselle@yandex.ru (A.S.); 3 Federal Research Centre Fundamentals of Biotechnology, Russian Academy of Science, 119071 Moscow, Russiaakamio@fbras.ru (A.K.); 4 Research and Educational Center “Applied Biotechnologies”, Immanuel Kant Baltic Federal University, 236041 Kaliningrad, Russia; olich.43@mail.ru; 5 Department of Pharmaceutical Chemistry and Bioanalytics, Institute of Pharmacy, Martin-Luther Universität Halle-Wittenberg, 06120 Halle, Germany; christian.ihling@pharmazie.uni-halle.de

**Keywords:** *Amaranthus caudatus*, zinc stress, bottom-up proteomics, metabolic adjustment

## Abstract

Zinc excess (Zn stress) could lead to deleterious effects in plants such as enhanced ROS production, inhibition of photosynthetic machinery, and impairment of nutrient uptake. Hence, we aimed to investigate the complexity of metabolic responses to Zn stress in *Amaranthus caudatus* young and mature leaves, as well as in roots by means of proteomics. Our previous metabolomics research has indicated potential involvement of gluconate and salicylate in Zn tolerance mechanisms. However, proteomics study of metabolic adjustments underlying Zn stress tolerance can give additional insight to the issue, as a lot of enzymes are known to be affected by the excess of transitional metals. The results obtained through bottom-up proteomics were complementary to our earlier metabolomics data and, furthermore, enlightened other important details in the metabolic response of *A. caudatus* plants to the applied Zn stress. In particular, the significant involvement of redox-related enzymes was shown, especially for the roots, and their possible interactions with salicylate and jasmonate signaling could be proposed. Furthermore, Zn^2+^-induced changes in roots and young leaves strongly affected sugar metabolism, enhanced protein quality control system, while mature leaves were characterized by remarkable decrease in subunits of photosynthetic electron transport complexes. Thus, this work emphasizes massive metabolic reprogramming aimed to reinforce root defense responses while supporting young leaves with sugar metabolites. Mass spectrometry proteomics data are available via ProteomeXchange with identifier PXD069557.

## 1. Introduction

Zinc (Zn) is one of the eight essential micronutrients [1] acting as a cofactor of multiple enzymes involved in signal transduction, protein biosynthesis, maturation and structure integrity, DNA replication and repair, detoxication of reactive oxygen species (ROS) [2,3]. In agreement with this, more than 2000 proteins in the *Arabidopsis thaliana* proteome were predicted to have Zn-binding cites [4] that might indicate high responsivity of plant proteome to Zn.

In plants, Zn uptake relies on the root absorption of Zn(II) cations, which are sufficiently abundant in soils, although in poorly soluble state [5]. Therefore, the equilibrium concentrations of free Zn^2+^ in the soil solution are quite low (typically not exceeding a few micromoles per L) [6] and essentially depend on the physicochemical properties of the soil, secretion activity of the plant roots, and metabolic features of the rhizosphere microbial community [2]. In agreement with this, plants typically accumulate Zn at relatively low levels (30–100 µg/g dry weight) [7]. These metabolically adequate contents can be efficiently managed by constitutive detoxification systems [8]. Accumulation of Zn(II) ions above these values results in overwhelming the plant metal detoxification capacity and development of heavy metal stress, which is typically accompanied by clear signs of toxicity [4].

Despite limited availability of Zn for its uptake by plant roots, several scenarios might lead to enhanced solubilization of the soil Zn pool [2]. Thus, acidified soils favor enhanced liberation of Zn from insoluble complexes and impact on development of Zn toxicity due to accumulation of this micronutrient in tissues [9]. Moreover, increased Zn contents might be underlied by prolonged application of phosphate-based fertilizers and by the use of sewage sludge [9]. Not less importantly, stressors such as drought or flooding can positively affect Zn availability and thereby favor higher Zn^2+^ concentrations in the soil solution [10]. Finally, accumulation of Zn in the plant tissues might be underlied by industrial activities and enhanced soil pollution [11].

From the point of plant physiology, high tissue Zn contents represent an abiotic stressor triggering a well-defined pattern of physiological responses: [9] decrease in seed germination rates, retardation of seedling growth [12], chlorosis in young leaves [13], necrosis and reddening lesions in mature leaves [14], decrease in photosynthetic efficiency [15], inhibition of nutrient [15,16] and water uptake [17], reduced stomatal activity [15]. To an essential extent, these effects can be attributed to overproduction of reactive oxygen species (ROS) and development of oxidative stress [18,19], which is, at least partly, underlied by impaired redox reactions in the electron transport chains (ETCs) of the chloroplasts and mitochondria [20]. Another toxicity mechanism might be associated with the loss of protein functionality due to the displacement of metal ions (particularly Fe^2+^/Fe^3+^, Mg^2+^, and Mn^2+^) by Zn^2+^ from their functional sites of enzymes [3].

Fortunately, plants possess well-established mechanisms of biochemical adaptation to Zn stress, such as the activation of antioxidant defense, enhanced synthesis of transport proteins involved in the uptake, partitioning and sequestration of Zn ions, as well as redirection of the central metabolism to cover the associated energy demand [18]. However, plants essentially differ in efficiency of the principal mechanism behind the tolerance to the Zn stress—in ability to rapidly compartmentalize Zn into vacuoles via transporters [21] and scavenging of Zn^2+^ in the cytosol by chelation with organic acids [22], amino acids (cysteine) [23], and glutathione [19].

The genus *Amaranthus* is not only a valuable source of nutrients and biologically active metabolites [24], but is also a highly adaptive crop [25] and a versatile tool for phytoremediation of Zn-contaminated agricultural soils [26]. In our previous work [27] we addressed the metabolic rearrangements triggered by the Zn stress in the roots and leaves of *A. caudatus* plants, and described the accompanying adaptive metabolic shifts therein. However, to address these metabolic changes in the most comprehensive way, the expressional changes at the proteome level (i.e., stress-induced dynamics of effector enzymes, transporters and ROS scavenging systems) need to be understood as well.

In this context, proteomics represents a versatile tool for disclosing the molecular mechanisms behind the Zn toxicity and plant tolerance to Zn stress, as was shown by Fukao et al. (2011) in *Arabidopsis thaliana* seedlings, Šimon et al. (2021) in *Oryza sativa* roots, and by Lucini et al. (2015) in *Lactuca sativa* leaves using gel-based and gel-free bottom-up approaches [28,29,30]. A study on *A. thaliana* seedlings grown under Zn excess can serve as a model to illustrate the significance of proteomics data in understanding the development of adaptive physiological reactions or Zn toxicity symptoms [30]. The seedling toxicity symptom such as chlorosis was associated with an increase in abundance of a few proteins known to be responsive to iron deficiency, indicating that Zn displaces iron from active sites of enzymes involved in chlorophyll synthesis. Another *A. thaliana* Zn toxicity symptom is a reduction in seedling root growth, associated with decrease in abundance of a few subunits of V-ATPase which are responsible for cell expansion and Zn^2+^ ion sequestration into vacuole [30]. However, despite the impressive body of the data accumulated so far, such a promising food crop as *A. caudatus* was still not addressed by the proteomics approach in respect of Zn stress.

Therefore, here we employ LC-MS-based bottom-up proteomics to address the metabolic responses of the *A. caudatus* root and leaves to moderate Zn stress. Thereby, we aimed to identify the Zn-responsive proteins in the roots, young and mature leaves of the *A. caudatus* plants exposed to Zn stress for seven days, as well as their comprehensive discussion in the context of the Zn^2+^-induced adaptive metabolic shifts discovered in these organs earlier [27].

## 2. Results

### 2.1. Suppressing Exposure to Zn Caused Suppression of the Photosystem II Efficiency in the A. caudatus Leaves

After two weeks of growing under the hydroponic conditions, the six-week-old *A. caudatus* plants were exposed to 300 µmol/L ZnSO_4_ × 7H_2_O supplemented to the nutrient solution. A one-week stress application did not cause severe alterations in leaf and root morphology in comparison to the age-matched untreated controls. The visibly detectable changes were observed only for the young leaves, which became light green upon application of the Zn stress (Supplementary Information 1, Figure S1-1).

**Figure 1:**
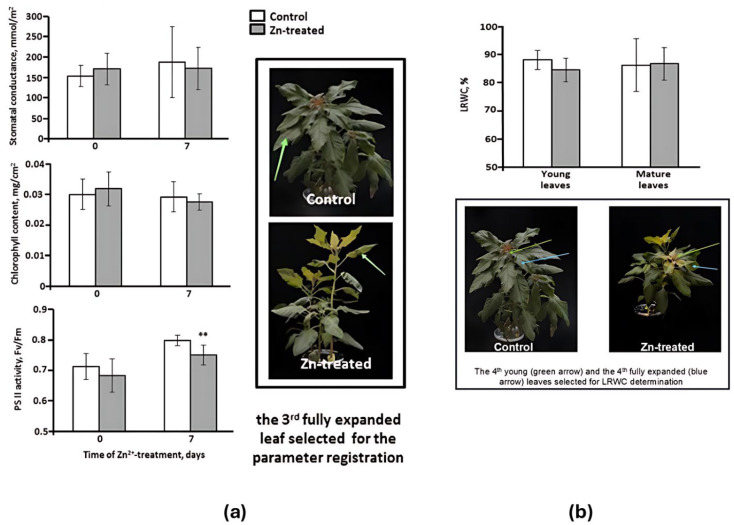
Impact of exogenous Zn^2+^ (300 μmol/L) on stomatal conductivity, chlorophyll content, photosystem II (PS II) activity (**a**), and leaf relative water content (**b**) of the third mature leaf (the leaf is indicated by green arrows at photos in the insert; numeration of the mature leaves were performed just beneath the plant top with young (i.e., not fully expanded) leaves) from six-week-old (before Zn^2+^-exposure, 0 day) and seven-week-old *A.caudatus* plants (*n* = 9) (after Zn^2+^-exposure, 7 day) grown in hydroponic nutrient solution. Gray columns indicate the Zn-treated group, namely plants whose physiological parameters were measured prior (0 day) and after the Zn-stress exposure (7 day). The Zn-stress exposure of the plants was performed by supplementation of 300 μmol/L ZnSO_4_ in hydroponic nutrient solution for one week. White columns indicate the control group of plants which were grown during the whole experimental period (from 0 to 7 days) in hydroponic nutrient solution without the addition of 300 μmol/L ZnSO_4_. F_v_/F_m_—variable fluorescence (F_v_)/maximal fluorescence (F_m_). Asterisks ** indicate significant difference between Zn-treated and control groups per time point at *p* (*t*-test) < 0.01. The experimental data obtained for a leaf of each plant are presented in Osmolovskaya et al. [27] Supplementary Information 2, Tables S2-1–S2-3).

Physiological assays accomplished by means of non-invasive methods for the 3rd fully expanded leaf revealed no significant changes in stomatal conductance, leaf relative water contents (LRWC), and chlorophyll contents upon Zn stress application in comparison to control plants (Figure 1a,b). However, as could be seen from the decrease in the F_v_/F_m_ values, photosystem II (PS II) efficiency appeared to be affected by stress conditions (Figure 1a,b). These results were also confirmed in the second independent experiment, which was accomplished to validate the initial findings (Figures S1-4, S1-2a and S1-3b).

### 2.2. Protein Isolation and Tryptic Digestion

The protein concentrations in the resulting young and mature leaves and roots extracts (assessed with the BCA assay) were in the range of 0.33–1.42 mg/mL, and the protein recoveries were 0.13–0.72 mg/g fresh weight (Table S1-1). Densitometric analysis of the electropherograms obtained with 5 µg of the total protein extracts loaded on each lane of the gels demonstrated comparable signal intensities per line across all gels normalized by the lines loaded with 5 µL protein ladder (50.85 × 10^4^ ± 68.19 × 10^3^ arbitrary units, RSD = 13%), indicating sufficient precision of protein assay. The following proteolysis, accomplished with 25 µg of the individual protein extracts could be considered as complete or close to complete as no distinct protein bands were detectable in the electropherograms acquired with filter remnants, assuming sensitivity of the Coomassie staining better than 30 ng (Figures S1-5 and S1-6) [31]. Unfocused signals at the very beginning of the lines most likely could be attributed to non-protein negatively charged biopolymers or glycoproteins.

### 2.3. Identification of Peptides and Annotation of Proteins

As the amaranth genome is still not established, for the database search we opted for the sequence of *C. quinoa*, which is the closest relative of *A. caudatus* with sequenced genome. The parallel search against the *A. thaliana* database (which contains more entries, although with a lower expected homology) yielded less numbers of confident matches. Therefore, the results of this search were not analyzed further.

The search against the *C. quinoa* protein sequence database resulted in the identification of 4608 peptides, comprising 1686 protein groups (non-redundant proteins) which represented 3163 possible individual proteins (Supplementary Information 2). To address the inter-organ differences in the absence and presence of the stress response, we accomplished inter-organ comparisons within each treatment group. This analysis, accomplished for the control group, yielded 2420 peptides comprising 873 non-redundant proteins, which could be assigned to 2205 possible individual proteins in all organs (Supplementary Information 1, Figure S1-7). Among these numbers, 444 peptides, 215 protein groups, and 663 individual proteins were common (18–30% of the total counts). Analogously, 2305 peptides, 856 protein groups, and 2120 proteins could be found in roots, young, and mature leaves of the Zn^2+^-treated plants. Thereby, 26–32% of the total protein numbers appeared to be common for all organs (491, 231, and 681 peptides, protein groups and proteins, respectively, Figure S1-8).

At the next step, the paired stress-control comparisons were addressed for each of the organs. The analysis revealed 1738 and 2870 peptides discovered in roots and leaves (young and mature collectively), respectively. These peptides represented 1145 and 2018 individual proteins, falling in 629 and 1057 protein groups (Figures S1-9–S1-11). In total, 568 protein groups with 797 possible individual proteins were detected in roots both in control and stress-exposed groups, while 44 and 17 non-redundant proteins (with 189 and 159 accessions) were found in the control or Zn-treated plants (Figure S1-9a,b; for the identified peptides behind these annotations, see Figure S1-9c). Considering the identifications for young and mature leaves separately, 574 and 483 non-redundant proteins (with 1077 and 941 accessions) could be annotated to these groups, respectively (Figures S1-10a,b and S1-11a,b; for the identified peptides behind these annotations, see Figures S1-10c and S1-11c). The numbers of the protein groups and individual proteins common for the young leaves from the control and Zn-treated groups were 535 and 783, respectively. On the other hand, 8 non-redundant proteins (136 accessions) were found only in the control samples, while 31 of those (158 accessions) could be annotated solely to the stress-exposed group (Figure S-10a,b; for the corresponding identified peptides behind, see Figure S1-10c).

Finally, 466 protein groups were common for the mature leaves harvested in all treatment groups, while 7 and 10 of them were found only in the controls and Zn-exposed ones. These non-redundant proteins could be attributed to 690 potential accessions (with 132 and 119 of them found only in the control and Zn-exposed groups, Figure S-11a,b; for identified peptides behind, see Figure S1-11c).

### 2.4. Label-Free Quantification

At the first step, we addressed the quantitative differences within the whole dataset in glance. For this, one-way ANOVA was accomplished at the confidence level of *p* ≤ 0.05 and the fold change (FC) threshold of 1.5 (both for increase and decrease in abundance in comparison to control). This multi-group comparison (in total 6 sample groups representing two treatments and three organs) revealed 355 non-redundant proteins, which were differentially expressed in at least 2 sample groups (Supplementary Information 3, Table S3-1).

**Figure 2:**
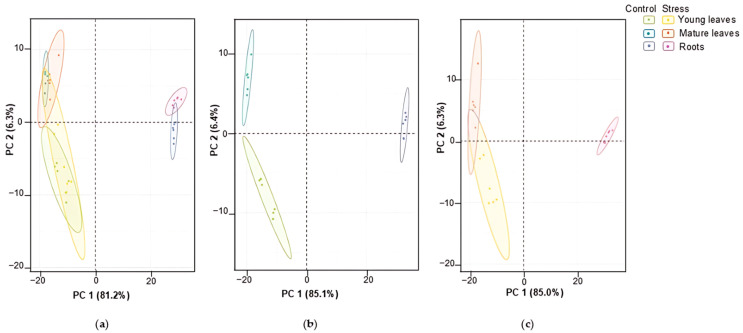
Principal component analysis with a scores plot performed for all treatment groups (roots, young, and mature leaves) with or without stress application (**a**), all groups of plants grown without Zn treatment (**b**), and all groups of plants grown under Zn stress conditions (**c**). PC—principal component; Control—plants grown without Zn treatment; Stress—plants grown under Zn stress (supplementation of 300 μmol/L Zn^2+^ to the hydroponic solution for 7 days).

Principal component analysis (PCA), accomplished for this differentially expressed part of the *A. caudatus* proteome, revealed clear separation in the organ-dependent manner with 81.2% of explained difference by the principal component 1 (PC1) visible in the corresponding scores plot (Figure 2a). Thus, the four leaf-related sample groups (representing the young and mature leaves collected from the control and the stress-exposed plants) clustered together and apart from the two root sample groups (control and stress).

The same pattern could be observed when the PCA tests were accomplished separately for all differentially expressed proteins annotated in the controls and in the Zn-exposed plants (360 and 375 differentially abundant non-redundant proteins, respectively; Tables S3-2 and S3-3). In both cases 85% of the total variance could be explained by PC1 (Figure 2b,c).

Based on corresponding loadings plots, the top 20 major contributors in these patterns could be assigned for each comparison (Figure S1-12). The analysis of these lists (Tables S1-2–S1-4) showed that the observed patterns of group separation were most likely defined by organ specificity and were not affected by the heavy metal treatment. It was especially relevant for the accessions expressed only in chloroplasts of the green parts.

Table S1-2 presents the five most responsive proteins (with their organ specificity) among all plant organs irrespective of Zn treatment, which were the major contributors in the observed differences. Thus, in roots of Zn-exposed plants, the germin-like protein appeared to be the positive contributor, whereas in the mature leaves of the control plants it was downregulated. UPF0603 followed an opposite pattern, being the most abundant in mature leaves and the least abundant in roots under control conditions. In contrast, the expression of the remaining three proteins was the highest in the control mature leaves but was the lowest in the roots of the Zn-exposed plants. The magnitude of these differences varied notably among the organs, ranging from 117.1 to 1461.8-fold.

Similarly, uncharacterized protein LOC110707738 isoform X1, UPF060, chloroplastic-like probable glutathione S-transferase parC isoform X1, chlorophyll *a*-*b* binding protein, and germin-like protein (72.9–infinite-fold) headlined the lists of the major contributors among all control groups (Table S1-3). Germin-like protein, peroxidase 12-like, NdhJ, putative pentatricopeptide repeat-containing protein, and UPF0603 (ranging from 214.4-fold to infinite-fold changes) were identified as the most significant contributors among all stress-treated groups (Table S1-4).

**Figure 3:**
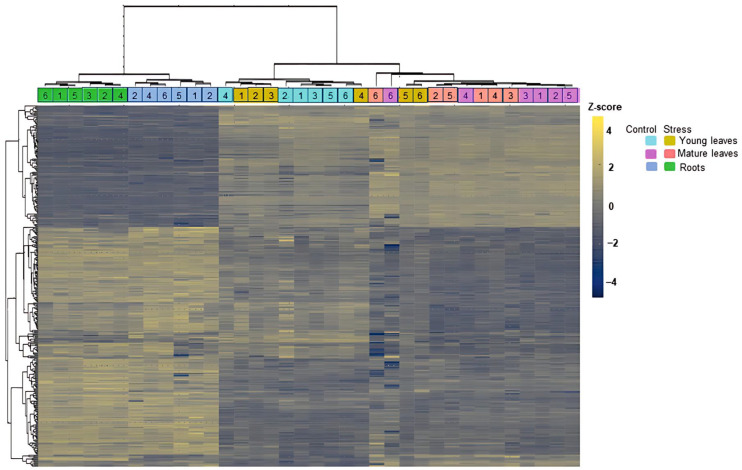
Hierarchical clustering with a heatmap representation showing expression patterns of individual samples performed for all the treatment groups (roots, young, and mature leaves) with or without stress application. Control—plants grown without Zn treatment; stress—plants grown under Zn stress (supplementation of 300 μmol/L Zn^2+^ to the hydroponic solution for 7 days). Figures indicate individual samples (n = 6) in each experimental group.

At the next step, hierarchical clustering analysis (HCA) with heatmap representation was accomplished for the whole sample set, which demonstrated comparable generalized results. The analysis revealed higher homogeneity of the root samples (obtained from both control and Zn-exposed plants), as could be seen by more distinct clustering of the corresponding groups at the column tree of the plot (Figure 3). On the other hand, the leaf samples (both young and mature) appeared to be distributed in a more or less uneven manner without any visible impact of the exposure to Zn (Figure 3).

Processing of samples from control and Zn-exposed plants separately revealed the same pattern for the roots (Figure S1-13a,b). In contrast to the above-described results of the global HCA test, the control mature and young leaf samples also formed apparent organ-specific clusters (Figure S1-13a), which were more or less dispersed upon stress exposure (Figure S1-13b).

Although the multiple comparisons by multivariate statistics and one-way ANOVA provided quite an informative general overview of the organ- and stress-specificity of the observed protein dynamics, they are still too complex for making direct and unambiguous conclusions about specific aspects of the plant response to Zn stress. Therefore, to address the response of individual plant organs to Zn stress in the most comprehensive way, we considered individual stress vs. control-paired comparisons at the level of each organ (Supplementary Information 4). The corresponding PCA tests revealed distinct stress-dependent inter-group separation in the corresponding scores plots with 52.8%, 40.8%, and 60.2% of the total explained variance for the roots, young, and mature leaves, respectively (Figures S1-14a–S1-16a).

In more detail, 78 root proteins appeared to be differentially expressed upon seven days of exposure to the Zn stress among 629 unambiguously annotated, with 34 and 44 of them up- and down-regulated in the Zn-exposed plants, respectively (*t*-test: *p* ≤ 0.05; FC ≥ 1.5, FDR correction at *q* ≤ 0.05; Tables S4-1 and S4-2). Analysis of the corresponding PCA data (and particularly the loadings plot) allowed the identification of the major contributors to the observed stress-related differences in the root differentially abundant proteins (Figure S1-14a,b; Table S1-5). Among them, the abundance of putative 12-oxophytodienoate reductase 11 isoform X1, probable glutathione S-transferase parC, chloroplast stem-loop binding protein of 41 kDa b, glutathione *S*-transferase-like protein, and probable inactive purple acid phosphatase 29 (PC1 0.09–0.15 absolute values; FC 1.6–27.7-fold) were significantly raised, while differential abundance level of proteins like plasma membrane ATPase 4-like, plasma membrane ATPase 4-like isoform X1, tubulin alpha-3 chain, ABC transporter F family member 3-like, and tubulin beta-5 chain-like were decreased (PC1 0.11–0.14; FC 1.7–3.5-fold). Further, heatmap cluster analysis was performed to highlight the differential accumulation patterns among proteins from the amaranth roots, where the clearest stress-related response could be observed (Figure S1-17).

Following the identification of the major patterns through PCA analysis, statistical evaluation further quantified the significance of differential protein accumulation. The statistical analysis thereby validated the observed clustering trends in the proteomics dataset. Increased level of protein abundances was observed for the probable glutathione S-transferase parC, putative 12-oxophytodienoate reductase 11 isoform X1, non-symbiotic hemoglobin 1-like, uncoupling protein 4-like, and S-transferase-like with the FC range of 10.6–39.1 (Table 1). Increased level of protein abundances was observed for such root proteins as peroxidase 3-like, ABC transporter F family member 3-like, aspartyl protease AED3-like, glutamine synthetase, and dihydrolipoyl dehydrogenase 2 (FC 2.3–5.0, Table 1).

For the young leaves, 40 proteins occurred as differentially expressed among 574 totally annotated, with 17 proteins demonstrating increased abundance and 23 proteins with decreased abundance (*t*-test: *p* ≤ 0.05; FC ≥ 1.5, FDR correction at *q* ≤ 0.05; Tables S4-3 and S4-4). Analysis of the PCA data by the first two principal components (PC1 and PC2) visualized via the corresponding scores and loadings plots allowed identification of the major contributors to the observed differences (Figure S1-15a,b; Table S1-10). Among them, CBS domain-containing protein CBSX3, dihydropyrimidine dehydrogenase (NADP^+^), plasma membrane ATPase 4-like, peroxiredoxin-2E-1, dihydrolipoyl dehydrogenase (PC1 0.17–0.22 absolute values; FC 1.5–2.0-fold) abundance were increased, while uncharacterized protein LOC110707738 isoform X1, 60S ribosomal protein L10-like, 60S ribosomal protein L4-like, magnesium-protoporphyrin IX monomethyl ester cyclase, PsbC showed significant decrease in its abundance (PC1 0.09–0.21; FC 1.6–3.0-fold). Additionally, heatmap cluster analysis was conducted to reveal differential accumulation patterns among proteins from the young leaves, where the most distinct stress-related effects were observed (Figure S1-18).

*T*-test results were in good agreement with the multivariate statistics data results. Proteins which demonstrated the most pronounced abundance increase under Zn-stress conditions were represented by the receptor-like protein 12, aspartate aminotransferase, CBS domain-containing protein CBSX3, ferredoxin-NADP reductase and probable aldo-keto reductase 4 (1.9–2.7-fold), and magnesium-protoporphyrin IX monomethyl ester cyclase, chloroplastic-like, PsbC protein, 60S ribosomal protein L4-like, stearoyl-[acyl-carrier-protein] 9-desaturase, and porphobilinogen deaminase comprised top of the list of the entries with the decreased level of abundance after Zn exposure (1.9–3.1-fold) (Table 2).

Mature leaf proteome accounted for 21 differentially accumulated accessions from the 483 annotated in total. Among these, 8 and 13 proteins appeared to be stress dependently up- and down-regulated, respectively (*t*-test: *p* ≤ 0.05; FC ≥ 1.5, FDR correction at *q* ≤ 0.05; Tables S4-5 and S4-6). PCA test results and loading plots for the mature leaf samples especially revealed eigenvalues of PC1 for the 20 major contributors (Figure S1-16a,b; Table S1-11). Thus, heat shock protein 83, probable glutathione S-transferase parC, betaine aldehyde dehydrogenase, annexin D2-like protein, citrate synthase comprised the group of the most differentially abundant proteins (PC1 0.14–0.22; FC 1.5–4.3-fold), while differential accumulation of PetA protein, catalase, photosystem II 10 kDa polypeptide, LRR receptor-like serine/threonine-protein kinase GSO1, 50S ribosomal protein L9 were significantly declined (PC1 0.21–0.27; FC 1.6–2.1-fold). Cluster analysis coupled with heatmap representation was carried out to emphasize the protein abundance level changes in the mature leaves, where the most pronounced stress-related responses were observed (Figure S1-19).

*T*-test results were consistent with the multivariate statistical data. The stress-induced proteins were represented by the heat shock protein 83, annexin D2-like protein, probable glutathione S-transferase, uncharacterized protein LOC110692268, and urease-like protein (1.7–4.2-fold). At the same time, callose synthase 10-like, PetA, NdhH, photosystem II 10 kDa polypeptide, magnesium-chelatase subunit ChlI were the top 5 proteins with decreased abundance level under Zn treatment (1.7–2.6-fold) (Table 3).

### 2.5. Functional Annotation of the Differentially Accumulated Proteins

Functional annotation of the differentially expressed proteins relied on the Mercator MapMan software (v 4.5) with the outputs being manually verified and curated based on the literature data. This was complemented by the advanced annotation workflow using open biological databases with an accession-based search (BRENDA Enzyme Database, Gene Ontology, Kyoto Encyclopedia of Genes and Genomes, The Arabidopsis Information Resource, UniprotKB).

**Figure 4:**
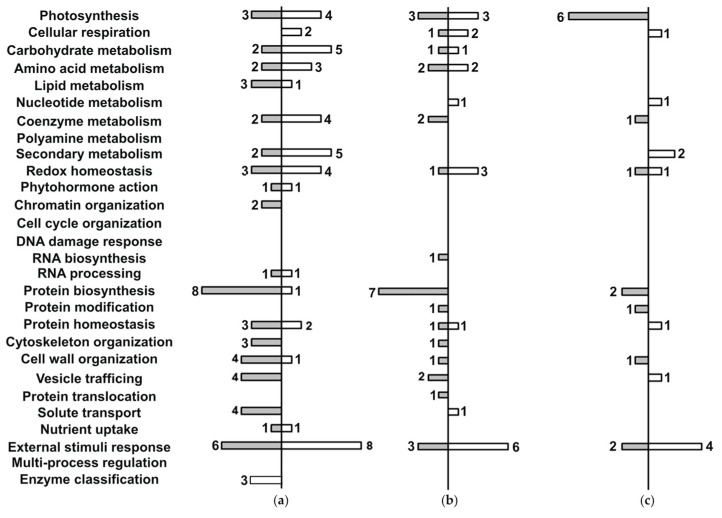
Functional annotation of the proteins identified as differentially expressed in the roots (**a**), young (**b**), and mature leaves (**c**) upon the exposure of the *A. caudatus* plants to 300 μmol/L Zn^2+^ supplemented to the hydroponic solution for 7 days (Zn stress) in comparison to the untreated controls. The bar labels indicate the numbers of proteins identified as up- (white) and down- (gray) regulated upon exposure to Zn stress. Protein annotation relied on Mercator MapMan (v4.5) software. Individual proteins comprising each functional class are listed in Supplementary Information 5.

Representation of individual functional classes was rich in the *A. caudatus* root proteome both in terms of the functional diversity (i.e., the number of annotated bins) and the numbers of entries per bin. All the differentially abundant root proteins were distributed among 20 bins out of 28 available (Figure 4a; Supplementary Information 5, Tables S5-1 and S5-2), containing 78 entries in general. Thereby, multiple proteins could be attributed to more than one cellular function. For three entries, no specific molecular function could be assigned. Therefore, these species were annotated as unknown. Such diversity of the proteome response could be attributed mostly to the proteins which demonstrated a down-regulation trend in their response to Zn stress.

Root proteins that demonstrated decrease in abundance when stress-induced comprised 18 functional classes (bins), while proteins with increased level of abundance were included in 16 bins. The latter part of the root proteome consisted of proteins involved in external stimuli response with eight entries (two glutathione S-transferases, uncoupling protein 4-like, probable aldo-keto reductase 4, heat shock protein 83) demonstrating a 2.4–39.1-fold abundance increase. Enzymes of the carbohydrate and secondary metabolism pathways also contributed to the observed differences with five proteins annotated within each functional class. The carbohydrate metabolism group included alcohol dehydrogenase 1 (10.1-fold), sucrose synthase-like (3.5-fold), phosphoglycerate kinase 3 (2.2-fold). Secondary metabolism protein functional class included probable glutathione S-transferase parC (39.1-fold), putative 12-oxophytodienoate reductase 11 (27.6-fold), BADH acyltransferase DCR-like (2.3-fold).

The external stimuli response functional class also dominated among root proteome with decreased level of protein abundance, where it was represented by six members—aspartyl protease AED3-like, several isoforms of the plasma membrane ATPase 4-like, and peroxidase 57-like, although in this case the stress-related proteins were less affected in comparison to the up-regulated ones (1.9–2.9 fold). Remarkably, in contrast to the proteins related to the stress response, the enzymes involved in protein biosynthesis were strongly suppressed under Zn stress conditions (eight entries in total). This group was represented by the ABC transporter F family member 3-like protein and different components of ribosomal machinery (1.5–3.5 fold-change).

The stress-related dynamics of the leaf proteins demonstrated different, although to some extent similar, patterns. Thereby, as can be seen from Figure 4, in leaves the Zn-induced changes were less pronounced in comparison to the roots. This effect could be seen both in terms of the numbers of the differentially expressed proteins and the degree of their abundance changes. Thus, only 40 differentially abundant species with the regulation degree not exceeding 3.1-fold were detected for the young leaves (in comparison to 78 differentially abundant proteins in roots, which demonstrated up to 39.1-fold changes).

The analysis of the young leaf proteome showed that 17 of 28 available functional classes (bins) appeared to be involved in the stress-related response in young leaves (Figure 4b, Tables S5-3 and S5-4). The down-regulated part of the leaf proteome was represented with 15 bins, whereas only 9 of them constituted its up-regulated part.

The proteins, discovered as Zn-dependently down-regulated in young leaves in response to the Zn treatment, dominated by the polypeptides involved in protein biosynthesis (seven entries). This functional class was represented by several ribosomal proteins and elongation factors TuB and 2-like (1.5–2.1-fold change) as the most responsive to the toxic metal. For the up-regulated part of the young leaf proteome, the group of the proteins responsive to external stimuli appeared to be the most abundant: in total, six proteins demonstrating a 1.7–2.7 abundance increase (receptor-like protein 12, probable aldo-keto reductase 4, polygalacturonase inhibitor-like, plasma membrane ATPase 4-like, and universal stress protein PHOS34-like) could be identified.

Finally, the proteome of the mature leaves appeared to be the least affected by Zn toxicity (Figure 4). Indeed, only 21 accessions representing only 12 bins (7 bins covering both increase and decrease in protein abundance level, with two representatives without specific function annotated) were differentially expressed in comparison to the untreated controls, which was approximately half of those discovered in young leaves (Figure 4c, Tables S5-5 and S5-6). Thereby, the proteins involved in responses to external stimuli (bin 26) appeared to be the most representative group of proteins with the increased abundance level, with four entries demonstrating up to 4.2-fold Zn-induced abundance change (heat shock protein 83, betaine aldehyde dehydrogenase, probable glutathione S-transferase parC, and annexin D2-like polypeptide). On the other hand, polypeptides involved in photosynthesis (six entries) were the most pronounced group with the decreased abundance level, although the degree of the abundance drop did not exceed 2.1-fold. The suppressed proteins of this class included PetA, NdhH, photosystem II 10 kDa polypeptide, cytochrome b6-f complex iron-sulfur subunit, NdhA.

Since this experiment revealed a relatively small number of Zn-responsive proteins (78, 40, and 21 for roots, young, and mature leaves, respectively), pathway enrichment analysis is unlikely to yield results for such small sets of differentially regulated proteins with fewer than 100 in each plant organ. Thus, further elucidation of important Zn-regulated metabolic pathways was based on numbers of Zn-responsive proteins assigned to a specific function. This highlighted at least six major protein functions: stress-response and redox metabolism, protein biosynthesis and homeostasis, photosynthesis, sugar metabolism, energy metabolism, and ion transport.

### 2.6. Prediction of Sub-Cellular Localization of Zn Stress-Exposed Differentially Accumulated Proteins

Prediction of the protein sub-cellular localization relied on BUSCA online tool with subsequent manual verification based on the literature data (Supplementary Information 6). This was further supported by annotation through an accession-based search in several open biological databases (BRENDA Enzyme Database, Gene Ontology, KEGG, The Arabidopsis Information Resource (TAIR), UniprotKB).

**Figure 5:**
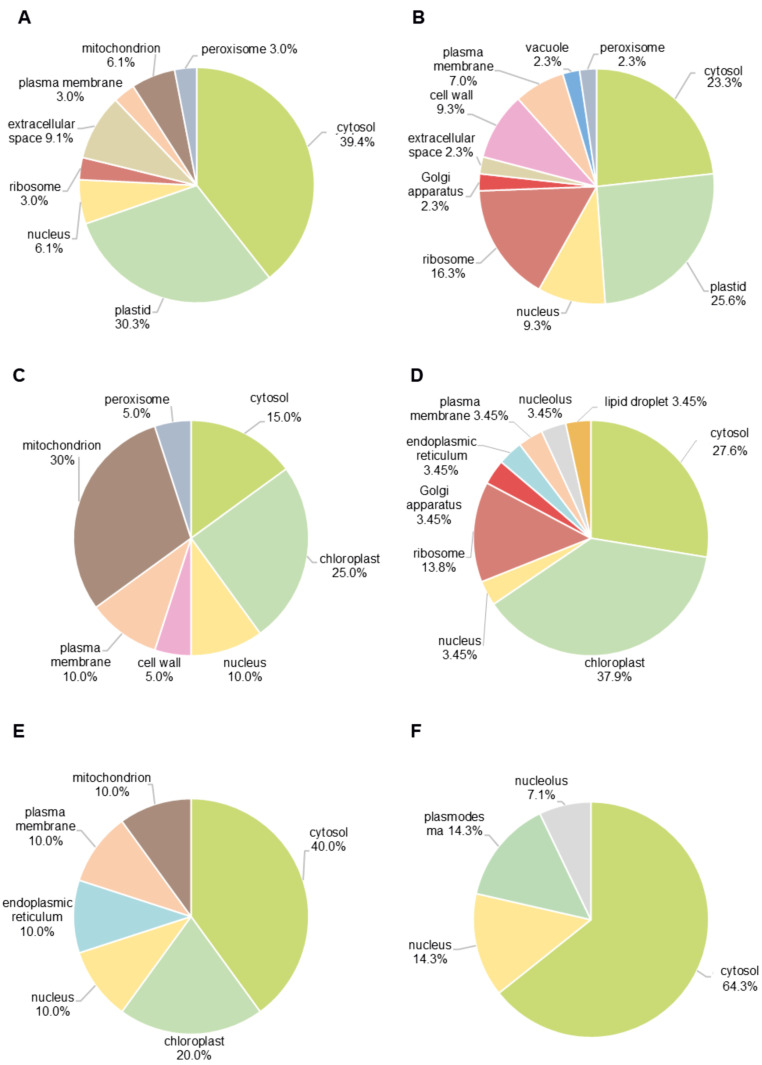
Prediction of sub-cellular localization of the differentially expressed proteins identified as up- (**A**,**C**,**E**) and down-regulated (**B**,**D**,**F**) in amaranth roots (**A**,**B**), young (**C**,**D**) and mature (**E**,**F**) leaves relied on BUSCA software. Individual proteins referred to each compartment are listed in Supplementary Information 6.

All accessions annotated as differentially accumulated in roots and leaves (both young and mature) were distributed among 17 cellular compartments, where cytosol (15–64% of proteins) and plastids (20–38% of proteins) tended to dominate in terms of the numbers of the individual proteins (Figure 5). The response to Zn stress in roots involved much higher diversity of compartments in comparison to the leaves, especially to the mature ones (Figure 5A,B). Remarkably, more than two-fold differences in the numbers of the up-regulated cytosolic proteins could be observed in the roots of the stress-exposed plants as compared to the young leaves collected from the same plants (Figure 5A,C, 39% and 15%, respectively).

In contrast to roots, stress response in leaves was accompanied with the changes in only seven compartments, predominantly cytosol, chloroplast, and nucleus (Figure 5E,F). On the other hand, the lowest contribution was shown for peroxisomes (0–5% of proteins), vacuoles (0–2% of proteins), and nucleoli (0–7% of proteins). Interestingly, abundances of ribosomal proteins were mostly decreased in response to Zn exposure with the highest numbers of affected polypeptides observed in roots (Figure 5B, 16%). In contrast, mitochondrial proteins were mostly increased in abundance with the most pronounced effect observed in the young leaves (Figure 5C, 30%). Surprisingly, proteins annotated to the Golgi apparatus were found only in the down-regulated part of the proteome, but this compartment was represented by only a few polypeptides (Figure 5B,D, 2–3%).

## 3. Discussion

With the growing involvement of heavy metals in the industrial production and household, high capacity of multiple plant species to accumulate pollutants of this type [32] has attracted particular attention since it can be efficiently used for soil remediation purposes [33]. While for some heavy metals (e.g., cadmium) no constitutive physiological role in plants has been found so far, others are known as essential micronutrients, critically impacting catalytic and structural functions of cellular proteins [9]. Although Zn belongs to the latter group, enhancement of its root absorption leads to clear manifestation of toxicity signs [9] and development of a well-defined stress response [2,34,35]. Understanding particular molecular mechanisms behind the Zn-associated toxicity and accompanying stress responses is critically important for the development of new approaches to attenuate deleterious metal-related physiological effects and to establish efficient phytoremediation tools.

Here, we employ the comprehensive bottom-up proteomics approach to characterize the Zn-related changes in the proteomes of roots, young, and mature leaves of *A. caudatus* plants exposed to high Zn^2+^ concentrations during their growing in a hydroponics system. The roots and leaves differentially responded to the stressor, which could be seen by pronounced difference in the qualitative and quantitative patterns of differentially abundant proteins (DAPs). The organ-specificity of DAPs patterns can be explained in the context of the roles of these organs in Zn stress response. Indeed, as roots represent the first barrier on the way of soil solutes to the plant organism, the most pronounced Zn-induced shifts in the plant proteome (78 DAPs) were observed in this organ. Young leaves were the second affected plant organ. Their relatively intense response (forty DAPs) might be explained by their high metabolic activity. In agreement with this, mature leaves with relatively low metabolic activity appeared to be the least affected (Figure 3; Table 1, Table 2 and Table 3). Results of our previous metabolomics study [27] were in good agreement with the results of the proteomics experiment presented here.

### 3.1. Plant Response to Zn^2+^ Was Accompanied by Characteristic Changes in Redox Metabolism and Stress-Inducible Proteome

Protein functional class related to the external stimuli responses (bin 26) was one of the most represented in all organs addressed. Moreover, a pattern of the proteins involved in the constitutive and stress-inducible antioxidative defense mechanisms (bin 10 “Redox homeostasis”) appeared to be affected in the metabolically active organs (roots and young leaves, Supplementary Information 5).

Enhancement in production of reactive oxygen species (ROS) is a universal reaction to any alterations in environment, which might be underlied by signaling events in terms of the stress response regulation [36] or stress-associated molecular damage due to overproduction of highly toxic radicals [37]. These reactions can be to some extent suppressed by the enzymatic antioxidant systems [38]. Here, the efficiency of these ROS-protective systems could be judged by the numbers of Zn-dependently up-regulated antioxidant enzymes, and by the degree of their up-regulation.

Thus, glutathione-*S*-transferase (GST), which was present in roots in two isoforms, showed a prominent up-regulation upon the exposure to Zn (Table S5-1). This effect can be explained by the role of this enzyme in the detoxification of highly reactive natural toxicants, such as lipid peroxides, by their conjugation with glutathione (GSH) [39]. On the other hand, this strong Zn-related increase in the relative abundance of GST can be explained by the direct involvement of GSH in detoxification of the Zn^2+^ ions which can be chelated directly by GSH to be transported in vacuole by GST [40,41].

This expressional response was in agreement with the shifts in the dynamics of the enzymes involved in the ascorbate-glutathione cycle: ascorbate peroxidase (APX, 1.7-fold down-regulated) and monodehydroascorbate reductase-like protein (MDHAR, 1.6-fold up-regulated, Tables S5-1 and S5-2) [42]. Such an increase in the abundance of the ascorbate-producing enzyme (MDHAR) might indicate enhancement in the ascorbic acid production and increase in its equilibrium concentration in cellular liquids. Ascorbate is not only a powerful scavenger of H_2_O_2_, but it plays a crucial role in the zeaxanthin synthesis (which is involved in dissipation of excess light energy under stress conditions) and reduction in catalytic transition metals in active centers of multiple ROS-detoxifying enzymes that is critical for maintaining their functionality [43].

On the other hand, decrease in the relative abundance of APX (present in two extracellular isoforms) and a concomitant rise in the levels of enzymes implicated in H_2_O_2_ production (several germin-like proteins) might indicate development of oxidative stress in amaranth roots (Tables S4-1 and S4-2). This transient increase in the tissue H_2_O_2_ contents might be a regulatory signal involved in the metabolic shifts associated with induction of protective phenylpropanoids and deposition of lignin in the cell wall [44].

It is known that H_2_O_2_ is a signaling agent employed in various defense responses and acting in a tight crosstalk with salicylate (SA) and jasmonate (JA) [45], activating the related signaling pathways, which are known to manage downstream activation of systemic tolerance mechanisms [46]. In line with this, the enzymes of JA metabolism were strongly affected by Zn stress. Thus, on one hand, probable linoleate 9S-lipoxygenase 5 (9-LOX5) and putative 12-oxophytodienoate reductase 11 (12-OPDAR), which are the key enzymes of the JA synthesis [47,48], appeared to be up-regulated in roots upon the exposure to Zn^2+^ (1.9- and 27.6- fold, respectively). On the other, peroxisomal 2-hydroxy-acid oxidase isoform X1, the principal enzyme of the JA metabolism, which is known to switch off the JA-signaling by hydroxylation of this hormone in its 12th position [49], was two-fold up-regulated in this organ. This concerted enhancement of JA biosynthesis and degradation might indicate strong involvement of H_2_O_2_-triggered JA-signaling in adaptation to Zn stress.

This assumption is supported by up-regulation of the root proteins, which are regulated by the JA- and SA-related pathways. For example, germin-like proteins, which positively impact on the tissue H_2_O_2_ levels [50] and play an important role in pathogen resistance (tightly regulated by JA-dependent mechanisms) [51], were found to be more than 1.5-fold up-regulated in roots. Further, non-symbiotic hemoglobin 1-like protein, which is known to be activated by JA and SA [52], was strikingly up-regulated in the roots (Table S5-1). This observation fitted well to the results of our metabolomics experiments [27], which indicated strong up-regulation of SA in roots and young leaves, highlighting a special role of SA in the mechanisms behind the plant tolerance to the Zn stress.

Similarly to the roots, the enzymes of the redox metabolism and an array of closely related proteins appeared to be the most responsive to the Zn exposure in the young leaves. This coordinated response suggests systemic regulation, where root-emerging redox shifts are transmitted with transporters, membrane receptors, and redox, signaling pathways to newly formed leaves [53]. The Zn-dependently regulated leaf proteome was predominantly represented by the proteins associated with response to external stimuli (bin 26, Tables S5-3 and S5-4, Figure 4b). A substantial part of these proteins was involved in signaling pathways and demonstrated differential patterns of the quantitative response. For example, leucine reached repeat (LRR)-containing receptor-like protein 12 and polygalacturonase inhibitor-like appeared to be Zn-dependently up-regulated, while the LRR-containing regulatory subunit of serine/threonine protein phosphatase 2A (PP2A) was down-regulated.

Currently, the LRR-containing proteins are the subject of extensive discussion with respect to various stressors, predominantly of the biotic nature [54,55]. The receptor-like protein 12 particularly, besides its function in stress protection, plays a pivotal role in the maintenance of meristems, serving as a CLV2-like peptide receptor in *A. thaliana* [56]. Polygalacturonase inhibitor protein is known to be induced by SA in response to biotic stress. It suppresses the activity of pathogen-secreted pectin-depolymerizing enzymes, which, in turn, limits the degree of cell-wall loosening [57]. Already at the beginning of this century, it was shown that pectin impacts binding heavy metal ions and prevents, thereby impacting their translocation into the cell [58]. PP2A is featured with broad specificity of substrates, and is known to be involved in auxin transport, ethylene and abscisic acid signaling, growth and development [59].

The described pattern of the Zn-induced protein expression shifts in young leaves might indicate the key role of the molecular signals transmitted therein from roots and mature leaves. The observed signature of signaling proteins might enable young leaves (as the most susceptible plant parts), to pre-adjust their metabolism for oncoming increase in tissue metal contents.

In contrast to the young leaves, the stress response of the mature leaf proteome dominated with a distinct pattern of up-regulated proteins as follows: heat shock protein 83 (HSP83), glutathione *S*-transferase parC (GST parC), and betaine aldehyde dehydrogenase (BADH). This response might highlight a general mechanism of the response to Zn stress, including maintaining the protein structure during/upon the stress exposure (HSP83) [60], conjugation of toxic compounds via GST [39], and accumulation of the osmolyte glycine betaine via BADH [61].

Annexin D2-like protein, a cytosolic polypeptide which was also found to be up-regulated in the mature leaves, is capable of relocating from cytosol to membranes (plasma membrane, ER, Golgi apparatus, vacuolar, nuclear, vesicles, etc. [62]) in response to enhanced cellular ROS production and increased cytosolic Ca^2+^ levels (Table S5-5) [63]. It is plausible to assume that both these stimuli are the parts of the regulatory signaling pathways including long-distance signal loops from roots. These effects, at least partly, are mediated by annexin D2, which likely activates defensive mechanisms [64].

Down-regulation of catalase more than 1.5-fold upon Zn treatment in mature leaves might be underlied by the signaling function of hydrogen peroxide, i.e., this might reduce H_2_O_2_ scavenging and thereby increase its equilibrium concentrations in tissue liquids. Catalase is known to be regulated by direct SA binding, which decreases the enzyme activity [65]. Thus, our observation is in agreement with the evidence for SA-repressed catalase mRNA expression described in the literature [66].

LRR receptor-like serine/threonine-protein kinase GSO1, the abundance of which was stress-dependently decreased in mature leaves, was intensively discussed in the context of root growth regulation and embryonic cuticle development [67,68]. Recently, GSO1 in combination with GSO2 was reported to be a positive regulator of cell proliferation [69]. Thus, it can be hypothesized that down-regulation of GSO1 in the mature amaranth leaves (Table S5-6) might be a signal linked to stress-related retardation of root and young leaf growth. This down-regulation of GSO1 protein likely designates stress-induced resource reallocation to prioritize defense processes under growth of new tissues [70].

### 3.2. Exposure to Zn^2+^ Affects Protein Biosynthesis and Homeostasis

Zn stress is known to cause protein damage, which is typically accompanied by their misfolding and aggregation [71]. In agreement with this, here we observed multiple alterations in the abundances of individual polypeptides associated with protein metabolism and structure maintenance (Figure 3). These shifts might be at least partly underlied by seven and five down-regulated ribosomal proteins (RPs) in amaranth roots and young leaves, respectively, and two transcript elongation factors (TEFs) in young leaves (Tables S5-2 and S5-4).

Now it is believed that besides their canonical structural role, RPs are implicated in additional specialized extraribosomal regulatory activities, which play important roles in the diversity of physiological processes [72]. Thus, it is proposed that some RPs could be capable of remodeling ribosomes for the synthesis of specific proteins that are required for a certain developmental stage or necessary for the response to defined biotic or abiotic stressor [73,74].

In addition, the regulatory function of RPs might be accomplished through interaction with specific proteins or/and metabolites—components of signaling pathways such as transcriptional factors and phytohormones [72]. Further, involvement of the TEF proteins in response to the applied Zn stress can be posited from suggestion that TEFs are implicated in transcriptional reprogramming, i.e., the process by which they regulate RNA polymerase II to generate transcripts adjusted for the specific demands of a given environment [75].

Another set of Zn-dependently regulated actors of protein metabolism was represented by the molecules involved in protein maturation and folding. Among them, stress-inducible HSP83 and proteases FTSH2 were up-regulated in the roots, whereas vacuolar-processing enzyme ClpA and aspartyl protease AED3-like protein appeared to be down-regulated (Tables S5-1 and S5-2). These results further suggest a multi-faceted protein protection mechanism, which includes protein refolding and protection against damage by heat shock proteins and proteases, and reducing the impact of impaired protein synthesis during ATP deficiency.

Among the accessions involved in proteolytic degradation in young leaves, the U-box domain containing 16-like protein was the only up-regulated protein from the group of ubiquitin ligases, which provided precisely tuned protein ubiquitination (Table S5-3) [76].

### 3.3. Zn Stress Results in Expressional Suppression of the Electron Transport Chain Proteins in the Chloroplast

Increased demand for energy and NAD(P)H supplementation is one of the most critical biochemical challenges associated with any stress response [77]. ATP and NAD(P)H are critically important for maintaining various metabolic processes including the activity of antioxidant system and correction of physiologically disadvantageous metabolic shifts [78].

The primary source of these energy-rich metabolites as well as organic carbon-rich substrates of energy metabolism is photosynthesis. However, the efficiency of this process in mature leaves is shown to be negatively affected by Zn stress [27]. Indeed, the excess of Zn might impair photosynthetic processes through disruption of chlorophyll synthesis by displacement of iron from active site of an enzyme that catalyze the formation of chlorophyll precursor, photochlorophilllide [30]; by substitution magnesium in the chlorophyll molecules [79]; and by causing direct damage of the proteins constituting electron transport chain (ETC) of the chloroplast [9].

In this study, we succeeded to obtain the first insight into these aspects at the proteome level. Thus, in agreement with the described scenario, we discovered the following six photosynthetic chloroplast ETC proteins which exhibited significant decrease in their abundance in mature leaves of Zn-exposed plants (Figure 4c): photosystems (PSs) I and II components (PsaA, PSII 10 kDa polypeptide), cytochrome proteins PetA and iron-sulfur subunit of cytochrome b_6_/f complex, NADH-dehydrogenases NdhH and NdhA (Table S5-6). These results might indicate the enhanced degradation of the ETC proteins under Zn stress, which, in turn, can lead to disturbances in electron transport and ATP production processes. The overload of the ETC with electrons and related overproduction of ROS can further damage ETC proteins, especially those containing iron [80].

The young leaves of the Zn-exposed plants which showed visible chlorosis symptoms displayed another pattern of DAPs implicated in photosynthetic ETC. Specifically, PsbC, NdhH, and UPF0603 (*A. thaliana* homolog At1g54780, annotated as thylakoid lumen protein 18.3 in TAIR) were downregulated, while ferredoxin-NADP^+^ reductase (FNR) and oxygen-evolving enhancer protein 2 (OEE2) were up-regulated (Tables S5-3 and S5-4). NdhH is a constituent of the protein complex implicated in cycled electron flow (CEF) around PSI [81]. This presents an alternative form of electron transport which is involved in ATP production under the conditions associated with high energy demand [82]. The down-regulation of NdhH in young leaves observed here might indicate the general impairment of photosynthetic complexes by Zn excess. The decline in the abundance of thylakoid lumen protein 18.3, regulating PSII reassembly, and PsbC, the core light-harvesting subunit of PSII, might stand for temporal cessation of PSII turnover in the stressful conditions by abovementioned direct Zn inhibition or as the result of oxidative damage [83].

The up-regulation of two other chloroplast proteins (FNR and OEE2) in young leaves under Zn stress probably reflects adaptive mechanisms against oxidative damage to maintain energy balance. Thus, an increased FNR abundance may suggest compensatory mechanisms accompanying disrupted efficiency of the photosynthetic machinery under Zn stress and compromised capacity of the Zn-exposed plant to supply NADPH equivalents for ROS counteraction and, most important, for sugars production in Calvin cycle [84]. OEE2 may serve to stabilize damaged PSII as its structural component [85].

In general, these findings indicate that exposure to high Zn^2+^ concentrations damages photosynthetic ETC or even prevents its establishment and chlorophyll biosynthesis in the young leaves causing their chlorosis. This, in turn, limits subsequent sugar synthesis. As a result of the decreased effectiveness of photosynthesis, young leaves have to compensate for the lack of monosaccharides by inducing their allocation from mature leaves.

### 3.4. Zn Stress Results in Rearrangement of Sugar and Energy Metabolism in A. caudatus Roots

In agreement with published data on the metabolic responses in heavy metal-exposed plants [86], our results indicate the inhibition of starch synthesis in the roots of *A. caudatus* plants grown under Zn stress. Indeed, fructose-1,6-bisphosphatase and glucose-1-phosphate adenylyltransferase—the two key enzymes involved in the early steps of starch biosynthesis—were found to be down-regulated in the Zn-affected root tissues.

Not less importantly, two sucrose synthase-like isoforms appeared to be Zn-induced in the amaranth roots (Tables S5-1 and S5-2). This enzyme is already known to be responsive to heavy metal ions with pronounced suppression of sucrose biosynthesis and activation of its degradation [87,88]. Thus, the results of our proteomics survey are in good agreement with observation of Li et al. Moreover, our proteomics data fit well with the results of the comprehensive metabolic profiling, accomplished for the same amaranth root material and clearly demonstrating a pronounced Zn-induced metabolic shift in favor of monosaccharides [27]. Some of the sugars might be involved in sugar signaling. Indeed, glucose produced by invertase may further maintain activity of hexokinase, one of major components of the sugar signaling cascades [89]. In fact, two hexokinase isoforms were annotated in amaranth roots. However, these enzymes did not exert Zn stress-associated expressional response (Tables S2-3 and S2-4, Tables S4-1 and S4-2). Interestingly, fructokinase, the primary fructose-phosphorylating enzyme, responded to Zn stress by increasing the number of isomers from 10 found in roots of control plants to 15 found in roots of Zn-treated plants. It is known that some of fructokinase isoforms could be inhibited by fructose in concentration exceeding 0.5–1 mmol/L [90] and it is proposed that fructokinase might act as a fructose sensor [91]. Differential expression patterns of different fructokinase isoforms were found in other plants (rice, sunflower) in response to various abiotic stresses [92,93]. Thus, our data, together with the literature data, suggest that under unfavorable conditions, different isoforms of fructokinase may play an important role in regulating the amount of metabolized carbohydrates.

Furthermore, three proteins representing bins 2 and 3 (Cellular respiration and Carbohydrate metabolism, respectively), namely 2,3-bisphosphoglycerate-independent phosphoglycerate mutase, phosphoglycerate kinase 3, and alcohol dehydrogenase 1 (Tables S5-1 and S5-2), were found to be upregulated under Zn stress conditions. In the context of the above discussed data on the stress-induced dynamics of the enzymes involved in starch and sucrose degradation, it can be inferred that energy metabolism serves as the principal pathway for the utilization of the overproduced monosaccharides in the roots of the Zn-exposed plants.

The up-regulation of glycolytic enzymes observed here can also be triggered by a decline in cellular energy status (i.e., in the ATP/ADP ratio [94]), and is currently regarded as a protective survival strategy under stress [95]. These changes are also consistent with the sugar signaling model, as evidenced by the role of Sucrose Non-fermenting-Related Kinase 1 (SnRK1) in this process [96]. This multimeric protein serves as a sugar sensor that is activated under energy-deprived conditions, such as stress, to promote catabolic pathways and downstream enhancement of defense proteins expression/activity [97]. On the other hand, Target of Rapamycin (TOR) kinase functions as an antagonist to SnRK1, promoting anabolic growth and ribosome biogenesis coordinating nutrient availability and energy status [98]. The expression levels of SnRK1 or TOR remained unchanged in response to zinc exposure in any of the plant organs in the present experiment. Nonetheless, it has been reported that these proteins are subject to regulation at the post-translational level, primarily through the process of phosphorylation [99], as well as through interactions with sugar phosphates, such as trehalose-6-phosphate [100]. In addition, a multitude of downstream targets have been identified [101]. The upregulation of several glycolytic enzymes discussed earlier, sucrose-degrading enzymes (sucrose synthase-like isoforms), and redox-related enzymes with heat shock protein 83 (also discussed above) in roots is consistent with the hypothesis that a sugar-regulated signal is generated, involving SnRK1 activation, which in turn activates catabolic processes and defense responses via transcriptional factors [102]. This signal emanated primarily more likely in mature leaves as a source tissue. Phloem-mobile sucrose is synthesized mainly in mature leaves and transported to sink tissues such as roots and young leaves, where it serves as substrate for energy generation and can influence energy-sensing pathways such as SnRK1, particularly in roots under stress conditions [96]. Furthermore, up-regulation of key enzymes of pyruvate dehydrogenase complex and TCA cycle (dihydrolipoyl dehydrogenase and citrate synthase, respectively) in the young leaves may occur in response to higher energy demands upon heavy metal stress, as these enzymes play crucial roles in energy metabolism and are often activated under conditions of nutrient stress to enhance metabolic efficiency and energy production [103]. The observed downregulation of multiple ribosomal proteins in root tissue in response to Zn exposure suggests that TOR signaling was likely repressed. The aforementioned repression led to a coordinated decrease in ribosomal biogenesis and overall protein biosynthesis as energy-intensive process.

Remarkably, such concerted shifts in the levels of abovementioned proteins in roots related to sugar metabolism, glycolysis, and fermentation were negligible in young and mature leaves. Nevertheless, our metabolomics study, accomplished with the same plant material [27], revealed a Zn-induced increase in the relative contents of soluble sugars in these organs, especially in young leaves. This finding suggests that the translocation of sugars from mature leaves rather than their *de novo* synthesis might underlie their up-regulation in the young leaves. Interestingly, this fact could not be supported by differential expression of sugar transporters in none of the studied organs of the Zn-exposed plants, although several of them were annotated in our dataset, e.g., sugar carrier protein C-like and sugar transport protein 1-like, both identified in roots and leaves (Supplementary Information 2). Thus, the plausible reason for the increase in the sugar relative contents could be changes in the process of translation and post-translational regulation of their transporters.

### 3.5. The Excess of the Zn(II) Ions Is Sequestered into the Vacuole and Cell Wall by Means of Several Transport Protein Systems

Exposure of the *A. caudatus* plants to Zn^2+^ caused clear alterations in the expressional patterns of membrane transporters. Thus, three isoforms of the root plasma membrane (P-type) H^+^-ATPase 4 and vacuolar (V-type) H^+^-ATPase subunit B2 appeared to be up to 2-fold down-regulated, which might cause compromised ability of the plant root to manage ionic balance and translocation under stress conditions [30,104,105]. On the other hand, multiple V-type H^+^-ATPases, annotated in the roots and leaves, did not show any stress-associated expressional response. This, however, does not ultimately indicate their unaffected functional state, as these V-type H^+^-ATPases are known to be regulated on the post-translational level (by phosphorylation) under the conditions of heavy metal stress (Cd, Cu, and Ni) [106].

The changes in the activity of P- and V-type H^+^-ATPases in roots (due to expressional or regulatory mechanisms) are critically important for suppression of Zn translocation to the shoot. This mechanism is implicated in the attenuation of Zn excess toxicity (i) by sequestering free Zn^2+^ ions or their chelates with low-molecular weight ligands from cytosol with the help of various types of Zn transporters (e.g., metal tolerance protein (MTP), vacuolar iron transporter (VIT), etc.) [9,107] or (ii) by promoting an accumulation of anionic metabolites (GSH, organic acids, amino acids, phytates, phosphate) in vacuoles, which further form complexes with Zn^2+^ [9,22]. This vacuolar sequestration of Zn^2+^ in root cells limits the ions translocation from roots to leaves and is regarded as one of HM tolerance mechanisms [108]. The proteomic datasets of amaranth roots and leaves studied here revealed no identification of any Zn transporters. Nevertheless, multiple representatives of ATP-binding cassette (ABC) transporters, a transporter group which was assumed to also be implicated in the translocation of free Zn^2+^ and their complexes across membranes into vacuoles [109], were annotated in all organs (Supplementary Information 2). However, no Zn-related alterations in their relative abundances could be observed in all organs (Supplementary Information 4). This fact might point to other mechanisms rather than transcriptional regulation that may be involved in control of ABC transporters. This might be the case here, as ABC transporters are often found to be post-translationally or epigenetically regulated [110,111]. The absence of the expressional response might be a species- or metal-specific feature. For example, transcriptional regulation of the ABC transporters was shown in tomato plants exposed to cadmium [112].

### 3.6. Consideration of the Zn-Induced Alterations in A. caudatus Proteome in the Context of the Accompanying Metabolic Adjustments Gives Access to the Mechanisms of Zn Stress Tolerance

**Figure 6:**
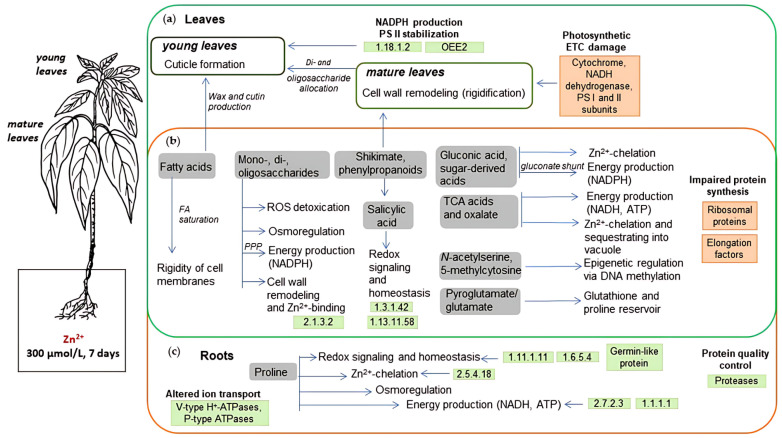
Consideration of the acquired proteomics data in the context of the metabolic shifts induced in the leaves and roots of the *A. caudatus* plants after their exposure to zinc in aq. culture (Zn^2+^, 300 μmol/L, 7 days, described earlier by Osmolovskaya et al. [27]). The scheme presents the alterations specific for leaves (**a**), common for leaves and roots (**b**), and specific for roots (**c**). The proteomic findings observed here are indicated with green (up-regulation) and red (down-regulation) rectangles as follows: 1.1.1.1—alcohol dehydrogenase; 1.11.1.11—ascorbate peroxidase; 2.1.3.2—probable inactive purple acid phosphatase 29; 1.18.1.2—ferredoxin-NADP^+^ reductase; 2.5.1.18—glutathione S-transferase; 1.6.5.4—monodehydroascorbate reductase; 2.7.2.3—phosphoglycerate kinase; 1.3.1.42—putative 12-oxophytodienoate reductase 11 isoform X1; 1.13.11.58—probable linoleate 9S-lipoxygenase 5; OEE2—oxygen evolving enhancer protein 2.

Generally, metabolic adjustments observed in our previous study on the same plant model [27] in response to Zn stress were more pronounced (both in terms of the numbers of affected metabolites and magnitude of corresponding shifts in their relative abundance) in the roots and young leaves than in the mature leaves. Thereby, multiple sugars and organic acids (in particular sugar acids) were the major classes of compounds to be strongly up-regulated in all organs. Generalized information relating to the further discussed principal changes in primary metabolism that occurred in the roots and leaves of *A. caudatus* plants in response to Zn stress and supported by findings of this proteomic research is presented in Figure 6.

Our proteomics data did not provide direct evidence of increased pectin synthesis, a common response to heavy metal stress in plants [113]. Indeed, increased pectin deposition in cell walls of plants upon Cd stress was associated with elevated expression of galacturonic acid transferase in tomato and potato, as well as with increased activity of pectin methylesterase [114]. Thus, both expression of key enzymes of pectin biosynthesis and pectin modifications (e.g., decreased methylesterification) influence heavy metal sequestration and, consequently, detoxification by cell wall. Thus, we can assume that regulation of this biopolymer does not rely mainly on the protein expression level in amaranth roots upon Zn stress. On the other hand, probable carbohydrate esterase At4g34215 likely belongs to SGNH-hydrolase superfamily of enzymes [115] and was down-regulated under Zn stress in the amaranth roots. Members of this superfamily are known for their ability to modify wide diversity of carbohydrates including pectins [116]. BAHD acyltransferase DCR-like up-regulated in roots could also be involved in pectin and cuticle remodeling [117]. The opposing regulation of these two cell-wall-modifying enzymes promotes the dynamic reconstruction of heteropolysaccharides in the root cell wall, improving Zn^2+^ chelation and counteracting their translocation into the shoot. Furthermore, the pattern of Zn-dependently up-regulated sugars dominated with galactose, which demonstrated a 9-fold increase in roots. This observation might be explained by the fact that galactose is putatively involved in the enhanced deposition of cell wall pectin, which has a high capacity for heavy metal binding [118].

Furthermore, galactose might be involved in the biosynthesis of galactolipids, which is known to be stimulated in response to phosphate starvation [119]. The deficit of phosphate might be underlied by enhanced involvement of this anion in chelation of Zn(II) ions under the conditions of their increased abundance in the plant tissues [120]. This assumption is confirmed by the fact that exposure of plants to high concentrations of Zn^2+^ results in low levels of phosphate in plants [121]. Our results also indicate the development of phosphate deficiency, which can be clearly seen from a 10-fold increase in the abundance of probable inactive purple acid phosphatase 29 (Table S5-1), which is a universally recognized indicator of this physiological state [122]. In the context of this finding, it is possible to assume that involvement of galactose in the biosynthesis of galactolipids can be activated by the substitution membrane phospholipids with galactolipids [123]. This is an adaptive strategy for membrane lipid remodeling during phosphate deprivation observed in several plants [119,123]. Additionally, LC-MS analysis of *N. caerulescens* leaves from Zn-treated plants revealed Zn-dependently altered galactolipid profiles, with a sharp increase in 12-oxo-phytodienoic acid (OPDA) contents, oxidized galactolipid species [124]. These changes fit well with the report of López-Orenes et al. on the heavy metal-induced SA-promoted oxylipin synthesis and the role of this oxylipin up-regulation in stress signaling and plant tolerance to abiotic stresses [125].

The role of SA in the plant response to the Zn exposure is another aspect to be discussed. In our earlier metabolomics survey [27] we observed a strong up-regulation of this phytohormone in young leaves and roots (23- and 27-fold, respectively). Although direct players involved in the SA biosynthetic pathway were not found in our proteomics dataset, multiple DAPs appeared to be involved in the processes, which, at least partly, rely on SA signaling. For example, SA is known to induce pathogenesis-related proteins, such as non-symbiotic hemoglobin 1-like protein in roots and polygalacturonase inhibitor-like in young leaves (Tables S4-1 and S4-3). Moreover, SA promotes biosynthesis of oxylipins [126], including jasmonates, which could be confirmed by the induction of putative 12-OPDAR and 9-LOX5 upon Zn stress in the roots (Table S4-1).

Increased (2–3.5-fold) expression of sucrose synthase in roots of Zn-treated plants (Table S4-1) might indicate stress-induced sucrose influx and its metabolism in roots. This enzyme together with hexose metabolizing hexokinases and fructokinases (see Section 4.4) supplies substrates for energy metabolism via their oxidation in glycolysis or PPP. This is in line with our previous metabolomics study which showed an increase in the levels of hexoses and, in particular, strong accumulation gluconic acid in roots and young leaves [27]. In plant cells gluconate acts as a powerful chelator of metal ions [127] and can be produced in the gluconate shunt of the pentose phosphate pathway (PPP) [128], described so far for bacteria, yeast, and fungi [129]. It is worth emphasizing that at the background of the ongoing oxidative stress, the gluconate formation, at least partly, may be caused by non-enzymatic glucose oxidation and thus contributes to metal-induced oxidative stress [27]. The presence of this phenomenon (i.e., enhanced ROS production) could be clearly seen by a 13-fold increase in the expression of mitochondrial uncoupling protein 4-like (Table S4-1), which discharges the mitochondrial membrane to reduce ROS production [130], and by a 2–5-fold reduction in the expression of five different peroxidases (Supplementary Information 3, Table S3-2). As a result, in conditions where sugar accumulation and oxidative stress occur simultaneously, generation of reactive carbonyl compounds (RCCs) might be activated, accompanied by the development of protein glycation and carbonyl stress [131]. This, in turn, may explain [132] the observed activation in expression of aldo-keto reductase (8-fold for probable aldo-keto reductase 4, Table S4-1) as a protein anti-glycation repair mechanism.

## 4. Materials and Methods

### 4.1. Plant Growth Conditions and Zn Stress Application

The detailed information on plant growth conditions was described in our previous work by Osmolovskaya et al. [27]. The study employed *Amaranthus caudatus* L. var. Karwa dauta. The seeds were provided by the Vavilov All-Russia Institute of Plant Genetic Resources, Saint Petersburg, Russia. The seeds were subjected to surface sterilization using a 3% (*v/v*) H_2_O_2_ solution for 20 min and rinsed with deionized water. The seeds were then germinated in containers filled with calcined quartz sand. The plants were cultivated in two separate experiments, each with an identical setup. Each experiment consisted of nine treatment groups, with a total of 27 plants distributed across nine vessels. The plants were grown under a controlled environment, with a 16 h day and 8 h night cycle, a relative humidity of 70–75%, and day/night temperatures of 24/18 °C. The plants were illuminated by fluorescent lamps (wavelength of 320–780 nm and a photosynthetic photon flux density of 120 µmol m^−2^ s^−1^).

In the initial seven days after the seeds germinated, the seedlings were irrigated with a ten-fold diluted nutrient solution (0.1 n.s., see Supplementary Information 1, Protocol S1-1) three times a day. Over three weeks, the concentration of the solution was gradually increased to 0.2 n.s., 0.5 n.s., and 1.0 n.s., respectively. After four weeks, the plants were transplanted into new 3 L hydroponic containers filled with the full-strength (1.0 n.s.) nutrient solution, with three plants per container.

After two weeks of growth in the hydroponic setup, the containers with six-week-old plants, featuring fully developed leaves that could be clearly distinguished from younger ones, were randomly divided into three equal groups (*n* = 3). The plants in the first group were harvested prior to the application of stress, for a separate evaluation of their root, young, and mature leaf biomass. The second group of plants was designated as the “Zn-treated” group with addition of 300 micromoles per liter of ZnSO_4_×7H_2_O to the nutrient solution. These stress conditions were chosen based on the previous research, including studies on amaranth [26]. The plants in the third group were considered as the control group and were not subjected to any treatment. The experiments were conducted in three independent biological replicates, with a total of nine plants in each group. The plants treated with zinc and the control plants were evaluated for a range of physiological characteristics (stomatal conductance, chlorophyll content, photosystem II activity, leaf water content) before (Day 0) and after the zinc stress (Day 7), and were then harvested for proteomics.

### 4.2. Protein Isolation and Digestion

The total leaf and root protein fractions were isolated from approximately 200 and 250 mg of fresh-frozen grounded leaf and root material per sample, respectively, by the phenol extraction method as it was described by Frolov et al. [133]. The resulting dry acetone protein pellets were reconstituted in 4% (*w/v*) aqueous (aq.) sodium dodecyl sulfate (SDS) under continuous sonication in 5 min cycles with ice cooling. Protein concentrations were determined by BCA assay kit according to the manufacturer’s protocol and cross-validated by sodium dodecyl sulfate polyacrylamide gel electrophoresis (SDS-PAGE) as described previously [134].

Aliquots of protein extracts (25 µg) were digested with trypsin according to the filter-aided sample preparation (FASP)-based protocol of Leonova et al. [135] with minimal changes (for details see Supplementary Information 1, Protocol S1-2). The completeness of protein digestion was verified with SDS-PAGE as described by Greifenhagen and co-workers [134]. The resulting tryptic hydrolysates were desalted and pre-cleaned with reverse-phase solid phase extraction (rpSPE) with six layers of C18 material-filled 200 µL tips (Merck KGaA, Darmstadt, Germany) according to the in-house established procedure [136]. The samples were dried under reduced pressure and stored at −20 °C until analysis.

### 4.3. Nano LC-MS/MS Experiments

Each of the samples containing dried tryptic hydrolysates were sequentially reconstituted in 60, 20, and 3% (*v*/*v*) aq. acetonitrile containing 0.1% (*v*/*v*) formic acid, and resulted samples were loaded on an Acclaim PepMap trap column (300 µm × 5 mm, 5 µm particle size, Thermo Fisher Scientific, Waltham, MA, USA) during 15 min at the flow rate of 30 µL/min. Peptides were separated on a RP C18 emitter column (PicoFrit, 75 μm × 250 mm, 15 μm tip diameter (New Objective) packed with ReproSil-Pur C18-AQ material, 1.9 μm, 120 Å, Dr. Maisch, Ammerbuch-Entringen, Germany) using an Ultimate 3000 RSLC nano chromatography system (Thermo Fisher Scientific, Waltham, MA, USA) coupled on-line to an Orbitrap XL mass spectrometer (Thermo Fisher Scientific, Waltham, MA, USA) operated in the positive ion mode via a nano-ESI source under the LC and MS settings specified in Tables S1-9 and S1-10, respectively. The nanoLC-ESI-Orbitrap-MS analysis relied on data-dependent acquisition (DDA) experiments, comprising in each cycle one survey Orbitrap-MS scan and dependent MS/MS scans for five the most abundant signals with the charge states from two to seven selected in the previous cycle.

### 4.4. Processing, Post-Processing, and Statistical Analysis of the Proteomics Data

The acquired LC-MS data were processed with Proteome Discoverer (v2.2, Thermo Fisher Scientific, Waltham, MA, USA) for identification of tryptic peptides and annotation of proteins (FDR correction at *q* ≤ 0.05 and Percolator node to filter high-confident identifications; for other search settings, see Table S1-11), whereas label-free quantification relied on QIProgenesis software (Waters GmbH, Eschborn, Germany). To select proteins that are significantly differentially abundant, thresholds for fold change (FC) and FDR adjusted *p*-value were set at 1.5 as the lowest value and 0.05 as the highest value, respectively. With this, proteins that showed an increase greater than 1.5-fold in abundance under Zn stress compared to the control (FC(Zn-stress/Control) ≥ 1.5) were considered as up-accumulated; proteins that showed a decrease greater than 1.5-fold in abundance under Zn stress compared to the control (FC(Control/Zn-stress) ≥ 1.5) were considered to be down-accumulated. Thus, the FC values were adjusted for cases of proteins showing a decrease in abundance by presenting the inverse of the FC value. For identification and annotation of proteins *Arabidopsis thaliana* (Uniprot) and *Chenopodium quinoa* polypeptide sequence databases uploaded from Uniprot and Kyoto Encyclopedia of Genes and Genomes (KEGG), respectively, on 12 September 2023 were used. The quantitative data outputs from QIProgenesis software were post-processed in R programming environment (v4.0.2, packages as follows: factoextra, pheatmap, VennDiagram) following log_2_-transformation. The differentially expressed proteins were annotated with Mercator MapMan platform (v4.5, https://plabipd.de/portal/mercator-sequence-annotation, accessed on 30 September 2023) to characterize functional roles and BUSCA online tool (http://busca.biocomp.unibo.it/, accessed on 1 October 2023) for sub-cellular localization prediction.

## 5. Conclusions

Comprehensive knowledge about the underlying mechanisms of plants adjustments and tolerance to heavy metals stress is critically important to achieve high efficiency of crops cultivation in polluted soils. In this regard, the integration of proteomics and metabolomics data is the best way to achieve this goal. The employed bottom-up proteomics approach revealed clear organ-specific responses to Zn exposure in *A. caudatus*, which were more pronounced in roots, indicating their crucial role as the first barrier on the way of pollutant uptake and distribution in plants. Our data indicated Zn-induced fine-tuned adaptive shifts in redox homeostasis, and enhancement of protein biosynthesis, repair/folding, and degradation systems. These adaptations, however, could not exclude the appearance of pronounced signs of toxicity and damage, like expressional suppression of ETC complexes in young and mature leaves.

Notably, the results of the proteomics study were consistent with the findings of the associated metabolomics research. Thus, the induction of SA-mediated protective reactions, as well as impact of SA on JA biosynthesis could be confirmed at the proteomics level. Furthermore, the strong up-regulation of gluconic acid (metal ion chelator in roots and young leaves) observed earlier was supported here by the induction of proteins forming soluble sugars for PPP which can also contribute to gluconate synthesis. Remarkably, while Zn-induced oxidative stress was evidenced by the upregulation of multiple antioxidative enzymes, a similar up-regulation was not observed for sugar transporters in roots and young leaves. Nevertheless, the annotation of several sugar transporters in these organs suggests the possibility of their regulation on post-translational level.

These findings may suggest that post-translational rather than transcriptional regulation is involved in fine-tuning control of the ion and sugar transport systems under Zn stress, which needs to be addressed in future studies.

To conclude, our results expand the existing knowledge on Zn-induced protein dynamics and give a better insight into the associated process of the metabolic adjustment as a part of the general response of plants to the Zn stress.

## Figures and Tables

**Table 1:** Top 20 most strongly regulated accessions among the 78 proteins constituting the part of amaranth root proteome, which was differentially expressed in response to Zn treatment.

Protein Name ^a^	KEGG Accession	FC ^b^	*p*	Bin ^c^	Subcellular Localization
Proteins displaying a higher abundance under Zn stress in comparison to control					
probable glutathione S-transferase parC	XP_021729368.1	39.1	8.6 × 10^−6^	9, 10, 26	cytoplasm, plastid, nucleus
putative 12-oxophytodienoate reductase 11 isoform X1	XP_021714783.1	27.6	7.3 × 10^−7^	9	cytoplasm, peroxisome
non-symbiotic hemoglobin 1-like	XP_021731656.1	15	1.0 × 10^−4^	10	cytoplasm, nucleus
mitochondrial uncoupling protein 4-like	XP_021725393.1	13.1	1.9 × 10^−4^	2, 26	mitochondrion
glutathione S-transferase-like	XP_021763022.1	10.6	2.3 × 10^−5^	10, 26	nucleus, cytoplasm, plasma membrane
probable inactive purple acid phosphatase 29	XP_021726654.1	10.5	5.0 × 10^−5^	25	cytoplasm
alcohol dehydrogenase 1	XP_021760951.1	10.1	1.9 × 10^−4^	3	cytoplasm
probable aldo-keto reductase 4	XP_021724433.1	8.2	5.0 × 10^−5^	26	mitochondrion
bifunctional riboflavin biosynthesis protein RIBA 1, chloroplastic-like	XP_021713589.1	5.9	2.3 × 10^−4^	7	plastid
LOW QUALITY PROTEIN: UPF0603 protein At1g54780, chloroplastic-like	XP_021772984.1	4.9	7.3 × 10^−3^	1, 19	plastid
Proteins displaying a decreased abundance under Zn stress in comparison to control					
peroxidase 3-like	XP_021726359.1	5	3.7 × 10^−4^	21	cell wall
ABC transporter F family member 3-like	XP_021757719.1	3.5	1.9 × 10^−4^	17	cytoplasm, nucleus
aspartyl protease AED3-like	XP_021752910.1	2.9	4.0 × 10^−4^	19, 26	cell wall
glutamine synthetase, chloroplastic	XP_021727743.1	2.4	1.4 × 10^−3^	4, 25	plastid
dihydrolipoyl dehydrogenase 2, chloroplastic-like	XP_021730249.1	2.3	2.7 × 10^−2^	5	plastid
peroxidase 72-like	XP_021714590.1	2.3	1.5 × 10^−2^	21	cell wall
vacuolar-processing enzyme-like	XP_021716668.1	2.2	3.7 × 10^−4^	19	vacuole
apoptosis-inducing factor 2-like	XP_021725188.1	2.1	4.4 × 10^−3^	35	cytoplasm
40S ribosomal protein S27-2	XP_021762421.1	2.1	2.9 × 10^−2^	17	ribosome, cytoplasm
plasma membrane ATPase 4-like	XP_021738895.1	2	6.2 × 10^−5^	24, 26	plasma membrane

^a^ Identification of peptides and annotation of proteins relied on the database search against amino acid sequences of *Chenopodium quinoa* (uploaded from KEGG protein database) accomplished with the SEQUEST algorithm; Mercator v4.5 was used for proteins annotation, subcellular localization of proteins was defined using BUSCA; ^b^ FC, fold change, for proteins that demonstrated an increase in abundance, FC(Zn stress/Control) indicates an (≥1.5-fold) increase in their abundance under stress Zn compared to the control, and for proteins that showed a decrease in abundance, FC(Control/Zn stress) shows a (≥1.5-fold) decrease in their abundance under Zn stress compared to the control. Thus, the FC values were adjusted for cases showing a decrease by presenting the inverse of the FC value; ^c^ Numbers correspond to the bins annotated with Mercator MapMan software (v4.5) (Table S1-6).

**Table 2:** Top 20 most strongly regulated accessions among the 40 proteins constituting the part of amaranth young leaf proteome, which was differentially expressed in response to Zn treatment.

Protein Name ^a^	KEGG Accession	FC ^b^	*p*	Bin ^c^	Subcellular Localization
Proteins displaying a higher abundance under Zn stress in comparison to control					
receptor-like protein 12	XP_021726832.1	2.7	4.1 × 10^−3^	26	plasma membrane
aspartate aminotransferase, cytoplasmic	XP_021723631.1	2.2	2.3 × 10^−2^	4	cytoplasm
CBS domain-containing protein CBSX3, mitochondrial-like	XP_021763137.1	2.2	4.4 × 10^−4^	10	mitochondrion
ferredoxin-NADP reductase, chloroplastic-like	XP_021727571.1	2.2	5.3 × 10^−3^	1	chloroplast
probable aldo-keto reductase 4	XP_021724433.1	2.0	3.3 × 10^−2^	26	mitochondrion
polygalacturonase inhibitor-like	XP_021724286.1	1.9	6.7 × 10^−3^	26	cell wall
plasma membrane ATPase 4-like	XP_021723433.1	1.9	7.7 × 10^−4^	24, 26	plasma membrane
enolase	XP_021743148.1	1.9	1.1 × 10^−2^	3	cytoplasm
dihydropyrimidine dehydrogenase (NADP^+^), chloroplastic-like	XP_021732698.1	1.8	3.8 × 10^−4^	6	chloroplast
peroxiredoxin-2E-1, chloroplastic-like	XP_021743149.1	1.8	1.1 × 10^−3^	10	chloroplast
Proteins displaying a decreased abundance under Zn stress in comparison to control					
magnesium-protoporphyrin IX monomethyl ester [oxidative] cyclase, chloroplastic-like	XP_021745614.1	3.1	1.7 × 10^−3^	7	chloroplast
PsbC (chloroplast)	YP_009380126.1	2.6	7.0 × 10^−3^	1	chloroplast
60S ribosomal protein L4-like	XP_021721538.1	2.1	6.5 × 10^−4^	17	ribosome, cytoplasm
stearoyl-[acyl-carrier-protein] 9-desaturase, chloroplastic	XP_021751679.1	2.0	2.5 × 10^−2^	4	chloroplast
porphobilinogen deaminase, chloroplastic-like	XP_021752880.1	1.9	8.1 × 10^−3^	7	chloroplast
translocase of chloroplast 159, chloroplastic-like	XP_021763183.1	1.9	4.2 × 10^−3^	23	chloroplast
granule-bound starch synthase 2, chloroplastic/amyloplastic-like	XP_021721398.1	1.8	1.1 × 10^−2^	3	chloroplast
NdhH (chloroplast)	YP_009380186.1	1.7	2.3 × 10^−2^	1	chloroplast
60S ribosomal protein L19-3-like	XP_021724307.1	1.7	8.4 × 10^−3^	17, 26	nucleolus
40S ribosomal protein S30	XP_021774723.1	1.7	1.5 × 10^−2^	17	ribosome, cytoplasm

^a^ Identification of peptides and annotation of proteins relied on the database search against amino acid sequences of *Chenopodium quinoa* (uploaded from KEGG protein database) accomplished with the SEQUEST algorithm; Mercator v4.5 was used for proteins annotation, subcellular localization of proteins was defined using BUSCA; ^b^ FC, fold change, for proteins that demonstrated an increase in abundance, FC(Zn stress/Control) indicates an (≥1.5-fold) increase in their abundance under stress Zn compared to the control, and for proteins that showed a decrease in abundance under Zn stress compared to the control, the FC values were adjusted by presenting the inverse of the FC value as FC(Control/Zn stress, ≥1.5); ^c^ Numbers correspond to the bins annotated with Mercator MapMan software (v4.5) (Table S1-6).

**Table 3:** Top 18 most strongly regulated accessions among the 21 proteins constituting the part of amaranth root proteome, which was differentially expressed in response to Zn treatment.

Protein Name ^a^	KEGG Accession	FC ^b^	*p*	Bin ^c^	Subcellular Localization
Proteins displaying a higher abundance under Zn stress in comparison to control					
heat shock protein 83	XP_021726736.1	4.2	5.1 × 10^−6^	26	cytoplasm
annexin D2-like	XP_021714165.1	2.1	5.9 × 10^−4^	22, 26	cytoplasm, plasma membrane
probable glutathione S-transferase parC	XP_021729368.1	2.1	7.2 × 10^−6^	9, 10, 26	cytoplasm, chloroplast, nucleus
uncharacterized protein LOC110692268	XP_021724961.1	2.0	1.5 × 10^−3^	-	extracellular space
low quality protein: urease-like	XP_021744615.1	1.8	6.1 × 10^−3^	6	cytoplasm
betaine aldehyde dehydrogenase, chloroplastic	XP_021733695.1	1.6	4.6 × 10^−5^	9, 26	chloroplast
citrate synthase, mitochondrial-like	XP_021731867.1	1.5	5.6 × 10^−4^	2	mitochondrion
luminal-binding protein-like	XP_021763741.1	1.5	5.050^−3^	19	endoplasmic reticulum
Proteins displaying a decreased abundance under Zn stress in comparison to control					
callose synthase 10-like	XP_021720217.1	2.6	1.0 × 10^−2^	21	plasma membrane
PetA (chloroplast)	YP_009380143.1	2.1	1.6 × 10^−4^	1	chloroplast
NdhH (chloroplast)	YP_009380186.1	1.8	1.1 × 10^−2^	1	chloroplast
photosystem II 10 kDa polypeptide, chloroplastic	XP_021748287.1	1.8	1.9 × 10^−3^	1	chloroplast
magnesium-chelatase subunit ChlI, chloroplastic-like	XP_021749286.1	1.7	8.9 × 10^−3^	7	chloroplast
ribosomal protein S14 (chloroplast)	YP_009380128.1	1.7	1.7 × 10^−2^	17	ribosome, chloroplast
LRR receptor-like serine/threonine-protein kinase GSO1	XP_021775210.1	1.7	3.1 × 10^−3^	18, 26	plasma membrane
uncharacterized protein LOC110707738 isoform X1	XP_021741469.1	1.7	2.1 × 10^−2^	-	chloroplast
50S ribosomal protein L9, chloroplastic	XP_021724417.1	1.6	3.7 × 10^−3^	17	ribosome, chloroplast
cytochrome b6/f complex iron-sulfur subunit, chloroplastic	XP_021726772.1	1.6	5.7 × 10^−3^	1	chloroplast

^a^ Identification of peptides and annotation of proteins relied on the database search against amino acid sequences of *Chenopodium quinoa* (uploaded from KEGG protein database) accomplished with the SEQUEST algorithm; Mercator v4.5 was used for proteins annotation; subcellular localization of proteins was defined using BUSCA; ^b^ FC, fold change, for proteins that demonstrated an increase in abundance; FC(Zn stress/Control) indicates an (≥1.5-fold) increase in their abundance under stress Zn compared to the control, and for proteins that showed a decrease in abundance under Zn stress compared to the control, the FC values were adjusted by presenting the inverse of the FC value as FC(Control/Zn stress, ≥1.5); ^c^ Numbers correspond to the bins annotated with Mercator MapMan software (v4.5) (Table S1-6).

## Data Availability

The original data presented in the study are openly available via the ProteomeXchange Consortium in the PRIDE [137] database with a unique identifier PXD069557 and 10.6019/PXD069557.

## Supplementary Materials

The following supporting information can be downloaded at https://www.mdpi.com/article/10.3390/plants14213315/s1, Supplementary Information 1: Protocols, Figures, and Tables; Supplementary Information 2: Peptides, proteins, and protein groups (non-redundant proteins), identified in amaranth (*Amaranthus caudatus*) plants under Zn stress conditions; Supplementary Information 3: Proteins, differentially expressed (ANOVA) in amaranth (*A. caudatus*) plants under Zn stress conditions; Supplementary Information 4: Proteins, differentially expressed (*t*-test) in amaranth (*A. caudatus*) plants under Zn stress conditions; Supplementary Information 5: Functional classes of proteins, differentially expressed in amaranth (*A. caudatus*) plants under Zn stress conditions; Supplementary Information 6: Prediction of localization of the proteins, differentially expressed in amaranth (*A. caudatus*) plants under Zn stress conditions.

## Abbreviations

9-LOX5, linoleate 9S-lipoxygenase 5; 12-OPDAR, 12-oxophytodienoate reductase; ABC, ammonium bicarbonate; APX, ascorbate peroxidase; BADH, betaine aldehyde dehydrogenase; BCA, bicinchoninic acid; CEF, cyclic electron flow; DAP, differentially abundant protein; DDA, data-dependent acquisition; ETC, electron transport chain; FC, fold change; FNR, ferredoxin-NADP^+^ reductase; GSH, reduced glutathione; GST, Glutathione S-transferase; HCA, hierarchical clustering analysis; IAA, iodoacetamide; JA, jasmonic acid; KEGG, Kyoto Encyclopedia of Genes and Genomes; LRR, leucine reach repeats; MDHA, monodehydroascorbate reductase; MS/MS, tandem mass spectrometry; OEE2, oxygen-evolving enhancer protein 2; PCA, Principal component analysis; PP2A, protein phosphatase; PPP, pentose phosphate pathway; PS, photosystem; RC, reactive carbonyl; ROS, reactive oxygen species; RP, ribosomal protein; RSD, relative standard deviation; SA, salicylic acid; SDS, sodium dodecyl sulfate; SDS-PAGE, sodium dodecyl sulfate polyacrylamide gel electrophoresis; SnRK1, Sucrose Non-fermenting-Related Kinase 1; TAIR, The Arabidopsis Information Resource; TEF, transcript elongation factor; TOR, Target of Rapamycin.

## References

[1] Ali A., Bhat B.A., Rather G.A., Malla B.A., Ganie S.A., Aftab T., Hakeem K.R. (2020). Proteomic Studies of Micronutrient Deficiency and Toxicity. Plant Micronutrients: Deficiency and Toxicity Management.

[2] Hamzah Saleem M., Usman K., Rizwan M., Al Jabri H., Alsafran M. (2022). Functions and Strategies for Enhancing Zinc Availability in Plants for Sustainable Agriculture. Front. Plant Sci..

[3] Stanton C., Sanders D., Krämer U., Podar D. (2022). Zinc in Plants: Integrating Homeostasis and Biofortification. Mol. Plant.

[4] Broadley M.R., White P.J., Hammond J.P., Zelko I., Lux A. (2007). Zinc in Plants. New Phytol..

[5] Claus J., Bohmann A., Chavarría-Krauser A. (2013). Zinc Uptake and Radial Transport in Roots of Arabidopsis Thaliana: A Modelling Approach to Understand Accumulation. Ann. Bot..

[6] Krämer U. (2025). Changing Paradigms for the Micronutrient Zinc, a Known Protein Cofactor, as a Signal Relaying Also Cellular Redox State. Quant. Plant Biol..

[7] White P.J., Broadley M.R. (2011). Physiological Limits to Zinc Biofortification of Edible Crops. Front. Plant Sci..

[8] Hall J.L. (2002). Cellular Mechanisms for Heavy Metal Detoxification and Tolerance. J. Exp. Bot..

[9] Kaur H., Garg N. (2021). Zinc Toxicity in Plants: A Review. Planta.

[10] Oyewo O.A., Adeniyi A., Bopape M.F., Onyango M.S. (2020). Heavy Metal Mobility in Surface Water and Soil, Climate Change, and Soil Interactions. Climate Change and Soil Interactions.

[11] Vardhan K.H., Kumar P.S., Panda R.C. (2019). A Review on Heavy Metal Pollution, Toxicity and Remedial Measures: Current Trends and Future Perspectives. J. Mol. Liq..

[12] Amin H., Arain B.A., Jahangir T.M., Abbasi A.R., Abbasi M.S., Amin F. (2023). Comparative Zinc Tolerance and Phytoremediation Potential of Four Biofuel Plant Species. Int. J. Phytoremediat..

[13] Wang C., Zhang S.H., Wang P.F., Hou J., Zhang W.J., Li W., Lin Z.P. (2009). The Effect of Excess Zn on Mineral Nutrition and Antioxidative Response in Rapeseed Seedlings. Chemosphere.

[14] Fontes R.L.F., Cox F.R. (1995). Effects of Sulfur Supply on Soybean Plants Exposed to Zinc Toxicity. J. Plant Nutr..

[15] Zemanová V., Pavlíková D., Novák M., Hnilička F. (2024). The Dual Role of Zinc in Spinach Metabolism: Beneficial × Toxic. Plants.

[16] Meng Y., Xiang C., Huo J., Shen S., Tang Y., Wu L. (2023). Toxicity Effects of Zinc Supply on Growth Revealed by Physiological and Transcriptomic Evidences in Sweet Potato (*Ipomoea batatas* (L.) Lam). Sci. Rep..

[17] Rucińska-Sobkowiak R. (2016). Water Relations in Plants Subjected to Heavy Metal Stresses. Acta Physiol. Plant.

[18] Balafrej H., Bogusz D., Triqui Z.-E.A., Guedira A., Bendaou N., Smouni A., Fahr M. (2020). Zinc Hyperaccumulation in Plants: A Review. Plants.

[19] Barrameda-Medina Y., Montesinos-Pereira D., Romero L., Blasco B., Ruiz J.M. (2014). Role of GSH Homeostasis under Zn Toxicity in Plants with Different Zn Tolerance. Plant Sci..

[20] Szopiński M., Sitko K., Gieroń Ż., Rusinowski S., Corso M., Hermans C., Verbruggen N., Małkowski E. (2019). Toxic Effects of Cd and Zn on the Photosynthetic Apparatus of the *Arabidopsis halleri* and Arabidopsis Arenosa Pseudo-Metallophytes. Front. Plant Sci..

[21] DalCorso G., Martini F., Fasani E., Manara A., Visioli G., Furini A. (2021). Enhancement of Zn Tolerance and Accumulation in Plants Mediated by the Expression of Saccharomyces Cerevisiae Vacuolar Transporter ZRC1. Planta.

[22] Sarret G., Saumitou-Laprade P., Bert V., Proux O., Hazemann J.-L., Traverse A., Marcus M.A., Manceau A. (2002). Forms of Zinc Accumulated in the Hyperaccumulator *Arabidopsis halleri*. Plant Physiol..

[23] Zeng X.-W., Ma L.Q., Qiu R.-L., Tang Y.-T. (2011). Effects of Zn on Plant Tolerance and Non-Protein Thiol Accumulation in Zn Hyperaccumulator *Arabis paniculata* Franch. Environ. Exp. Bot..

[24] Rastogi A., Shukla S. (2013). Amaranth: A New Millennium Crop of Nutraceutical Values. Crit. Rev. Food Sci. Nutr..

[25] Riggins C.W., Barba de la Rosa A.P., Blair M.W., Espitia-Rangel E. (2021). Editorial: Amaranthus: Naturally Stress-Resistant Resources for Improved Agriculture and Human Health. Front. Plant Sci..

[26] Lukatkin A.S., Bashmakov D.I., Al Harbawee W.E.Q., Teixeira da Silva J.A. (2021). Assessment of Physiological and Biochemical Responses of *Amaranthus retroflexus* Seedlings to the Accumulation of Heavy Metals with Regards to Phytoremediation Potential. Int. J. Phytoremediat..

[27] Osmolovskaya N., Bilova T., Gurina A., Orlova A., Vu V.D., Sukhikh S., Zhilkina T., Frolova N., Tarakhovskaya E., Kamionskaya A. (2025). Metabolic Responses of *Amaranthus caudatus* Roots and Leaves to Zinc Stress. Plants.

[28] Lucini L., Bernardo L. (2015). Comparison of Proteome Response to Saline and Zinc Stress in Lettuce. Front. Plant Sci..

[29] Šimon M., Shen Z.-J., Ghoto K., Chen J., Liu X., Gao G.-F., Jemec Kokalj A., Novak S., Drašler B., Zhang J.-Y. (2021). Proteomic Investigation of Zn-Challenged Rice Roots Reveals Adverse Effects and Root Physiological Adaptation. Plant Soil..

[30] Fukao Y., Ferjani A., Tomioka R., Nagasaki N., Kurata R., Nishimori Y., Fujiwara M., Maeshima M. (2011). iTRAQ Analysis Reveals Mechanisms of Growth Defects Due to Excess Zinc in Arabidopsis. Plant Physiol..

[31] Frolov A., Blüher M., Hoffmann R. (2014). Glycation Sites of Human Plasma Proteins Are Affected to Different Extents by Hyperglycemic Conditions in Type 2 Diabetes Mellitus. Anal. Bioanal. Chem..

[32] Nouri J., Khorasani N., Lorestani B., Karami M., Hassani A.H., Yousefi N. (2009). Accumulation of Heavy Metals in Soil and Uptake by Plant Species with Phytoremediation Potential. Environ. Earth Sci..

[33] Yan A., Wang Y., Tan S.N., Mohd Yusof M.L., Ghosh S., Chen Z. (2020). Phytoremediation: A Promising Approach for Revegetation of Heavy Metal-Polluted Land. Front. Plant Sci..

[34] Nagajyoti P.C., Lee K.D., Sreekanth T.V.M. (2010). Heavy Metals, Occurrence and Toxicity for Plants: A Review. Environ. Chem. Lett..

[35] Subba P., Mukhopadhyay M., Mahato S.K., Bhutia K.D., Mondal T.K., Ghosh S.K. (2014). Zinc Stress Induces Physiological, Ultra-Structural and Biochemical Changes in Mandarin Orange (*Citrus reticulata* Blanco) Seedlings. Physiol. Mol. Biol. Plants.

[36] Hasanuzzaman M., Bhuyan M.H.M.B., Parvin K., Bhuiyan T.F., Anee T.I., Nahar K., Hossen M.S., Zulfiqar F., Alam M.M., Fujita M. (2020). Regulation of ROS Metabolism in Plants under Environmental Stress: A Review of Recent Experimental Evidence. Int. J. Mol. Sci..

[37] Tripathy B.C., Oelmüller R. (2012). Reactive Oxygen Species Generation and Signaling in Plants. Plant Signal. Behav..

[38] Das K., Roychoudhury A. (2014). Reactive Oxygen Species (ROS) and Response of Antioxidants as ROS-Scavengers during Environmental Stress in Plants. Front. Environ. Sci..

[39] Skórzyńska-Polit E., Drążkiewicz M., Krupa Z. (2010). Lipid Peroxidation and Antioxidative Response in Arabidopsis Thaliana Exposed to Cadmium and Copper. Acta Physiol. Plant.

[40] Khan M., Samrana S., Zhang Y., Malik Z., Khan M.D., Zhu S. (2020). Reduced Glutathione Protects Subcellular Compartments From Pb-Induced ROS Injury in Leaves and Roots of Upland Cotton (*Gossypium hirsutum* L.). Front. Plant Sci..

[41] Waśkiewicz A., Gładysz O., Szentner K., Goliński P. (2014). Role of Glutathione in Abiotic Stress Tolerance. Oxidative Damage to Plants.

[42] Gill S.S., Anjum N.A., Hasanuzzaman M., Gill R., Trivedi D.K., Ahmad I., Pereira E., Tuteja N. (2013). Glutathione and Glutathione Reductase: A Boon in Disguise for Plant Abiotic Stress Defense Operations. Plant Physiol. Biochem..

[43] Hasanuzzaman M., Bhuyan M.H.M.B., Anee T.I., Parvin K., Nahar K., Mahmud J.A., Fujita M. (2019). Regulation of Ascorbate-Glutathione Pathway in Mitigating Oxidative Damage in Plants under Abiotic Stress. Antioxidants.

[44] Xiao T., ShangGuan X., Wang Y., Tian Z., Peng K., Shen Z., Hu Z., Xia Y. (2024). The Germin-like Protein OsGLP8-7 Is Involved in Lignin Synthesis for Acclimation to Copper Toxicity in Rice. J. Plant Physiol..

[45] Tamás L., Mistrík I., Huttová J., Halušková L., Valentovičová K., Zelinová V. (2010). Role of Reactive Oxygen Species-Generating Enzymes and Hydrogen Peroxide during Cadmium, Mercury and Osmotic Stresses in Barley Root Tip. Planta.

[46] Leon J., Lawton M.A., Raskin I. (1995). Hydrogen Peroxide Stimulates Salicylic Acid Biosynthesis in Tobacco. Plant Physiol..

[47] Qi X., Chen M., Liang D., Xu Q., Zhou F., Chen X. (2020). Jasmonic Acid, Ethylene and ROS Are Involved in the Response of Cucumber (*Cucumis sativus* L.) to Aphid Infestation. Sci. Hortic..

[48] León Morcillo R.J., Ocampo J.A., García Garrido J.M. (2012). Plant 9-Lox Oxylipin Metabolism in Response to Arbuscular Mycorrhiza. Plant Signal. Behav..

[49] Miersch O., Neumerkel J., Dippe M., Stenzel I., Wasternack C. (2008). Hydroxylated Jasmonates Are Commonly Occurring Metabolites of Jasmonic Acid and Contribute to a Partial Switch-off in Jasmonate Signaling. New Phytol..

[50] Ilyas M., Rasheed A., Mahmood T. (2016). Functional Characterization of Germin and Germin-like Protein Genes in Various Plant Species Using Transgenic Approaches. Biotechnol. Lett..

[51] Mao L., Ge L., Ye X., Xu L., Si W., Ding T. (2022). ZmGLP1, a Germin-like Protein from Maize, Plays an Important Role in the Regulation of Pathogen Resistance. Int. J. Mol. Sci..

[52] Qu Z.-L., Zhong N.-Q., Wang H.-Y., Chen A.-P., Jian G.-L., Xia G.-X. (2006). Ectopic Expression of the Cotton Non-Symbiotic Hemoglobin Gene GhHbd1 Triggers Defense Responses and Increases Disease Tolerance in Arabidopsis. Plant Cell Physiol..

[53] Oteiza P.I. (2012). Zinc and the Modulation of Redox Homeostasis. Free Radic. Biol. Med..

[54] Dang P.M., Lamb M.C., Bowen K.L., Chen C.Y. (2019). Identification of Expressed R-Genes Associated with Leaf Spot Diseases in Cultivated Peanut. Mol. Biol. Rep..

[55] Zhou H., Xie Y., Jiang Y., Nadeem H., Wang Y., Yang N., Zhu H., Tang C. (2023). GhTLP1, a Thaumatin-like Protein 1, Improves Verticillium Wilt Resistance in Cotton via JA, ABA and MAPK Signaling Pathway-Plant Pathways. Int. J. Biol. Macromol..

[56] Zrenner R., Verwaaijen B., Genzel F., Flemer B., Grosch R. (2021). Transcriptional Changes in Potato Sprouts upon Interaction with Rhizoctonia Solani Indicate Pathogen-Induced Interference in the Defence Pathways of Potato. Int. J. Mol. Sci..

[57] Rathinam M., Rao U., Sreevathsa R. (2020). Novel Biotechnological Strategies to Combat Biotic Stresses: Polygalacturonase Inhibitor (PGIP) Proteins as a Promising Comprehensive Option. Appl. Microbiol. Biotechnol..

[58] Połeć-Pawlak K., Ruzik R., Lipiec E., Ciurzyńska M., Gawrońska H. (2007). Investigation of Pb(ii) Binding to Pectin in Arabidopsis Thaliana. J. Anal. At. Spectrom..

[59] País S.M., Téllez-Iñón M.T., Capiati D.A. (2009). Serine/Threonine Protein Phosphatases Type 2A and Their Roles in Stress Signaling. Plant Signal. Behav..

[60] Neumann D., Lichtenberger O., Günther D., Tschiersch K., Nover L. (1994). Heat-Shock Proteins Induce Heavy-Metal Tolerance in Higher Plants. Planta.

[61] Fitzgerald T.L., Waters D.L.E., Henry R.J. (2009). Betaine Aldehyde Dehydrogenase in Plants. Plant Biol..

[62] Konopka-Postupolska D., Clark G. (2017). Annexins as Overlooked Regulators of Membrane Trafficking in Plant Cells. Int. J. Mol. Sci..

[63] Dufoo-Hurtado M.D., Huerta-Ocampo J.Ã., Barrera-Pacheco A., Barba De La Rosa A.P., Mercado-Silva E.M. (2015). Low Temperature Conditioning of Garlic (*Allium sativum* L.) Â€œseedâ€ Cloves Induces Alterations in Sprouts Proteome. Front. Plant Sci..

[64] Mortimer J.C., Laohavisit A., Macpherson N., Webb A., Brownlee C., Battey N.H., Davies J.M. (2008). Annexins: Multifunctional Components of Growth and Adaptation. J. Exp. Bot..

[65] Rüffer M., Steipe B., Zenk M.H. (1995). Evidence against Specific Binding of Salicylic Acid to Plant Catalase. FEBS Lett..

[66] Sun T., Liu F., Wang W., Wang L., Wang Z., Li J., Que Y., Xu L., Su Y. (2018). The Role of Sugarcane Catalase Gene ScCAT2 in the Defense Response to Pathogen Challenge and Adversity Stress. Int. J. Mol. Sci..

[67] Chen C., He G., Li J., Perez-Hormaeche J., Becker T., Luo M., Wallrad L., Gao J., Li J., Pardo J.M. (2023). A Salt Stress-activated GSO1-SOS2-SOS1 Module Protects the *Arabidopsis* Root Stem Cell Niche by Enhancing Sodium Ion Extrusion. EMBO J..

[68] Creff A., Brocard L., Joubès J., Taconnat L., Doll N.M., Marsollier A.-C., Pascal S., Galletti R., Boeuf S., Moussu S. (2019). A Stress-Response-Related Inter-Compartmental Signalling Pathway Regulates Embryonic Cuticle Integrity in Arabidopsis. PLoS Genet..

[69] Racolta A., Bryan A.C., Tax F.E. (2014). The Receptor-like Kinases GSO1 and GSO2 Together Regulate Root Growth in *Arabidopsis* through Control of Cell Division and Cell Fate Specification. Dev. Dyn..

[70] Zhu Q., Feng Y., Xue J., Chen P., Zhang A., Yu Y. (2023). Advances in Receptor-like Protein Kinases in Balancing Plant Growth and Stress Responses. Plants.

[71] Tamás M., Sharma S., Ibstedt S., Jacobson T., Christen P. (2014). Heavy Metals and Metalloids As a Cause for Protein Misfolding and Aggregation. Biomolecules.

[72] Stępiński D. (2025). Decoding Plant Ribosomal Proteins: Multitasking Players in Cellular Games. Cells.

[73] Mazahar M., Achala B., Anusree S., Kirti P.B. (2019). Ribosomal Proteins and Their Extra Ribosomal Functions in Abiotic Stress Tolerance of Plants. Mod. Concepts Dev. Agron..

[74] Fakih Z., Germain H. (2025). Implication of Ribosomal Protein in Abiotic and Biotic Stress. Planta.

[75] Grasser K.D. (2025). The Role of RNA Polymerase II Transcript Elongation Factors in Plant Stress Responses. J. Exp. Bot..

[76] Mao X., Yu C., Li L., Wang M., Yang L., Zhang Y., Zhang Y., Wang J., Li C., Reynolds M.P. (2022). How Many Faces Does the Plant U-Box E3 Ligase Have?. Int. J. Mol. Sci..

[77] Chai M., Wei P., Chen Q., An R., Chen J., Yang S., Wang X. (2006). NADK3, a Novel Cytoplasmic Source of NADPH, Is Required under Conditions of Oxidative Stress and Modulates Abscisic Acid Responses in Arabidopsis. Plant J..

[78] Valderrama R., Corpas F.J., Carreras A., Gómez-Rodríguez M.V., Chaki M., Pedrajas J.R., Fernández-Ocaña A., Del Río L.A., Barroso J.B. (2006). The Dehydrogenase-mediated Recycling of NADPH Is a Key Antioxidant System against Salt-induced Oxidative Stress in Olive Plants. Plant Cell Environ..

[79] Moustakas M., Dobrikova A., Sperdouli I., Hanć A., Moustaka J., Adamakis I.-D.S., Apostolova E. (2024). Photosystem II Tolerance to Excess Zinc Exposure and High Light Stress in *Salvia sclarea* L. *Agronomy*
**2024**, *14*, 589. Agronomy.

[80] Petrov V., Hille J., Mueller-Roeber B., Gechev T.S. (2015). ROS-Mediated Abiotic Stress-Induced Programmed Cell Death in Plants. Front. Plant Sci..

[81] Zhang C., Shuai J., Ran Z., Zhao J., Wu Z., Liao R., Wu J., Ma W., Lei M. (2020). Structural Insights into NDH-1 Mediated Cyclic Electron Transfer. Nat. Commun..

[82] Shuyskaya E., Rakhmankulova Z., Prokofieva M., Kazantseva V., Lunkova N. (2023). Impact of Salinity, Elevated Temperature, and Their Interaction with the Photosynthetic Efficiency of Halophyte Crop *Chenopodium quinoa* Willd. Agriculture.

[83] Huihui Z., Xin L., Zisong X., Yue W., Zhiyuan T., Meijun A., Yuehui Z., Wenxu Z., Nan X., Guangyu S. (2020). Toxic Effects of Heavy Metals Pb and Cd on Mulberry (*Morus alba* L.) Seedling Leaves: Photosynthetic Function and Reactive Oxygen Species (ROS) Metabolism Responses. Ecotoxicol. Environ. Saf..

[84] Potters G., Horemans N., Jansen M.A.K. (2010). The Cellular Redox State in Plant Stress Biology—A Charging Concept. Plant Physiol. Biochem..

[85] Bai J., Qin Y., Liu J., Wang Y., Sa R., Zhang N., Jia R. (2017). Proteomic Response of Oat Leaves to Long-Term Salinity Stress. Environ. Sci. Pollut. Res..

[86] Yang Y., Wang S., Zhao C., Jiang X., Gao D. (2024). Responses of Non-Structural Carbohydrates and Biomass in Plant to Heavy Metal Treatment. Sci. Total Environ..

[87] Cai S.-W., Huang W.-X., Xiong Z.-T., Ye F.-Y., Ren C., Xu Z.-R., Liu C., Deng S.-Q., Zhao J. (2013). Comparative Study of Root Growth and Sucrose-Cleaving Enzymes in Metallicolous and Non-Metallicolous Populations of Rumex Dentatus under Copper Stress. Ecotoxicol. Environ. Saf..

[88] Li C., Liu Y., Tian J., Zhu Y., Fan J. (2020). Changes in Sucrose Metabolism in Maize Varieties with Different Cadmium Sensitivities under Cadmium Stress. PLoS ONE.

[89] Varshney V., Singh J., Salvi P., Sharma D., Singh S., Sharma S.K., Singh R. (2023). Sugar Signaling and Their Interplay in Mitigating Abiotic Stresses in Plant: A Molecular Perspective. Smart Plant Breeding for Field Crops in Post-Genomics Era.

[90] German M.A., Asher I., Petreikov M., Dai N., Schaffer A.A., Granot D. (2004). Cloning, Expression and Characterization of LeFRK3, the Fourth Tomato (*Lycopersicon esculentum* Mill.) Gene Encoding Fructokinase. Plant Sci..

[91] Granot D., Kelly G., Stein O., David-Schwartz R. (2014). Substantial Roles of Hexokinase and Fructokinase in the Effects of Sugars on Plant Physiology and Development. J. Exp. Bot..

[92] Fulda S., Mikkat S., Stegmann H., Horn R. (2011). Physiology and Proteomics of Drought Stress Acclimation in Sunflower (*Helianthus annuus* L.). Plant Biol..

[93] Guglielminetti L., Morita A., Yamaguchi J., Loreti E., Perata P., Alpi A. (2006). Differential Expression of Two Fructokinases in *Oryza sativa* Seedlings Grown under Aerobic and Anaerobic Conditions. J. Plant Res..

[94] Bui L.T., Novi G., Lombardi L., Iannuzzi C., Rossi J., Santaniello A., Mensuali A., Corbineau F., Giuntoli B., Perata P. (2019). Conservation of Ethanol Fermentation and Its Regulation in Land Plants. J. Exp. Bot..

[95] Jardine K.J., McDowell N. (2023). Fermentation-mediated Growth, Signaling, and Defense in Plants. New Phytol..

[96] Wurzinger B., Nukarinen E., Nägele T., Weckwerth W., Teige M. (2018). The SnRK1 Kinase as Central Mediator of Energy Signaling between Different Organelles. Plant Physiol..

[97] Li G., Zhao Y. (2024). The Critical Roles of Three Sugar-Related Proteins (HXK, SnRK1, TOR) in Regulating Plant Growth and Stress Responses. Hortic. Res..

[98] Busche M., Scarpin M.R., Hnasko R., Brunkard J.O. (2021). TOR Coordinates Nucleotide Availability with Ribosome Biogenesis in Plants. Plant Cell.

[99] Crepin N., Rolland F. (2019). SnRK1 Activation, Signaling, and Networking for Energy Homeostasis. Curr. Opin. Plant Biol..

[100] Tsai A.Y.-L., Gazzarrini S. (2014). Trehalose-6-Phosphate and SnRK1 Kinases in Plant Development and Signaling: The Emerging Picture. Front. Plant Sci..

[101] Han C., Wang H., Shi W., Bai M.-Y. (2024). The Molecular Associations between the SnRK1 Complex and Carbon/Nitrogen Metabolism in Plants. New Crops.

[102] Peixoto B., Baena-González E. (2022). Management of Plant Central Metabolism by SnRK1 Protein Kinases. J. Exp. Bot..

[103] Angulo-Bejarano P.I., Puente-Rivera J., Cruz-Ortega R. (2021). Metal and Metalloid Toxicity in Plants: An Overview on Molecular Aspects. Plants.

[104] Fukao Y., Ferjani A. (2011). V-ATPase Dysfunction under Excess Zinc Inhibits Arabidopsis Cell Expansion. Plant Signal. Behav..

[105] Li J., Guo Y., Yang Y. (2022). The Molecular Mechanism of Plasma Membrane H+-ATPases in Plant Responses to Abiotic Stress. J. Genet. Genom..

[106] Janicka-Russak M., Kabala K., Burzynski M., Klobus G. (2008). Response of Plasma Membrane H+-ATPase to Heavy Metal Stress in *Cucumis sativus* Roots. J. Exp. Bot..

[107] Ulhassan Z., Sheteiwy M.S., Khan A.R., Hamid Y., Azhar W., Hussain S., Salam A., Zhou W. (2025). Zinc Toxicity in Plants: A Brief Overview on Recent Developments. Zinc in Plants.

[108] Verbruggen N., Hermans C., Schat H. (2009). Molecular Mechanisms of Metal Hyperaccumulation in Plants. New Phytol..

[109] Do T.H.T., Martinoia E., Lee Y., Hwang J.-U. (2021). 2021 Update on ATP-Binding Cassette (ABC) Transporters: How They Meet the Needs of Plants. Plant Physiol..

[110] I. Stolarczyk E., J. Reiling C., M. Paumi C. (2011). Regulation of ABC Transporter Function Via Phosphorylation by Protein Kinases. CPB.

[111] Yadav S., Kalwan G., Gill S.S., Jain P.K. (2025). The ABC Transporters and Their Epigenetic Regulation under Drought Stress in Chickpea. Plant Physiol. Biochem..

[112] Hashmi S.S., Lubna, Bilal S., Jan R., Asif S., Abdelbacki A.M.M., Kim K.-M., Al-Harrasi A., Asaf S. (2025). Exploring the Role of ATP-Binding Cassette Transporters in Tomato (*Solanum lycopersicum*) under Cadmium Stress through Genome-Wide and Transcriptomic Analysis. Front. Plant Sci..

[113] Kosakivska I.V., Babenko L.M., Romanenko K.O., Korotka I.Y., Potters G. (2021). Molecular Mechanisms of Plant Adaptive Responses to Heavy Metals Stress. Cell Biol. Int..

[114] Jia H., Wang X., Wei T., Zhou R., Muhammad H., Hua L., Ren X., Guo J., Ding Y. (2019). Accumulation and Fixation of Cd by Tomato Cell Wall Pectin under Cd Stress. Environ. Exp. Bot..

[115] Bitto E., Bingman C.A., McCoy J.G., Allard S.T.M., Wesenberg G.E., Phillips G.N. (2005). The Structure at 1.6 Å Resolution of the Protein Product of the At4g34215 Gene from *Arabidopsis thaliana*. Acta Crystallogr. D Biol. Crystallogr..

[116] Anderson A.C., Stangherlin S., Pimentel K.N., Weadge J.T., Clarke A.J. (2022). The SGNH Hydrolase Family: A Template for Carbohydrate Diversity. Glycobiology.

[117] Panikashvili D., Shi J.X., Schreiber L., Aharoni A. (2009). The Arabidopsis *DCR* Encoding a Soluble BAHD Acyltransferase Is Required for Cutin Polyester Formation and Seed Hydration Properties. Plant Physiol..

[118] Doyama K., Yamaji K., Haruma T., Ishida A., Mori S., Kurosawa Y. (2021). Zn Tolerance in the Evergreen Shrub, Aucuba Japonica, Naturally Growing at a Mine Site: Cell Wall Immobilization, Aucubin Production, and Zn Adsorption on Fungal Mycelia. PLoS ONE.

[119] Pfaff J., Denton A.K., Usadel B., Pfaff C. (2020). Phosphate Starvation Causes Different Stress Responses in the Lipid Metabolism of Tomato Leaves and Roots. Biochim. Biophys. Acta (BBA) Mol. Cell Biol. Lipids.

[120] Bouain N., Shahzad Z., Rouached A., Khan G.A., Berthomieu P., Abdelly C., Poirier Y., Rouached H. (2014). Phosphate and Zinc Transport and Signalling in Plants: Toward a Better Understanding of Their Homeostasis Interaction. J. Exp. Bot..

[121] Ding J., Liu L., Wang C., Shi L., Xu F., Cai H. (2021). High Level of Zinc Triggers Phosphorus Starvation by Inhibiting Root-to-Shoot Translocation and Preferential Distribution of Phosphorus in Rice Plants. Environ. Pollut..

[122] Sabet M.S., Zamani K., Lohrasebi T., Malboobi M.A., Valizadeh M. (2018). Functional Assessment of an Overexpressed Arabidopsis Purple Acid Phosphatase Gene (Atpap26) in Tobacco Plants. Iran. J. Biotechnol..

[123] Wang F., Ding D., Li J., He L., Xu X., Zhao Y., Yan B., Li Z., Xu J. (2020). Characterisation of Genes Involved in Galactolipids and Sulfolipids Metabolism in Maize and Arabidopsis and Their Differential Responses to Phosphate Deficiency. Funct. Plant Biol..

[124] Foroughi S., Baker A.J.M., Roessner U., Johnson A.A.T., Bacic A., Callahan D.L. (2014). Hyperaccumulation of Zinc by *Noccaea caerulescens* Results in a Cascade of Stress Responses and Changes in the Elemental Profile. Metallomics.

[125] López-Orenes A., Martínez-Pérez A., Calderón A.A., Ferrer M.A. (2014). Pb-Induced Responses in *Zygophyllum fabago* Plants Are Organ-Dependent and Modulated by Salicylic Acid. Plant Physiol. Biochem..

[126] Liu W., Park S.-W. (2021). 12-Oxo-Phytodienoic Acid: A Fuse and/or Switch of Plant Growth and Defense Responses?. Front. Plant Sci..

[127] Kavita B., Shukla S., Naresh Kumar G., Archana G. (2008). Amelioration of Phytotoxic Effects of Cd on Mung Bean Seedlings by Gluconic Acid Secreting Rhizobacterium Enterobacter Asburiae PSI3 and Implication of Role of Organic Acid. World J. Microbiol. Biotechnol..

[128] Kornecki J.F., Carballares D., Tardioli P.W., Rodrigues R.C., Berenguer-Murcia Á., Alcántara A.R., Fernandez-Lafuente R. (2020). Enzyme Production of d -Gluconic Acid and Glucose Oxidase: Successful Tales of Cascade Reactions. Catal. Sci. Technol..

[129] Corkins M.E., Wilson S., Cocuron J.-C., Alonso A.P., Bird A.J. (2017). The Gluconate Shunt Is an Alternative Route for Directing Glucose into the Pentose Phosphate Pathway in Fission Yeast. J. Biol. Chem..

[130] Ledesma A., De Lacoba M., Rial E. (2002). The mitochondrial uncoupling proteins. Genome Biol..

[131] Bilova T., Lukasheva E., Brauch D., Greifenhagen U., Paudel G., Tarakhovskaya E., Frolova N., Mittasch J., Balcke G.U., Tissier A. (2016). A Snapshot of the Plant Glycated Proteome. J. Biol. Chem..

[132] Sengupta D., Naik D., Reddy A.R. (2015). Plant Aldo-Keto Reductases (AKRs) as Multi-Tasking Soldiers Involved in Diverse Plant Metabolic Processes and Stress Defense: A Structure-Function Update. J. Plant Physiol..

[133] Frolov A., Bilova T., Paudel G., Berger R., Balcke G.U., Birkemeyer C., Wessjohann L. (2017). Early Responses of Mature Arabidopsis Thaliana Plants to Reduced Water Potential in the Agar-Based Polyethylene Glycol Infusion Drought Model. J. Plant Physiol..

[134] Greifenhagen U., Frolov A., Blüher M., Hoffmann R. (2016). Site-Specific Analysis of Advanced Glycation End Products in Plasma Proteins of Type 2 Diabetes Mellitus Patients. Anal. Bioanal. Chem..

[135] Leonova T., Ihling C., Saoud M., Frolova N., Rennert R., Wessjohann L.A., Frolov A. (2022). Does Filter-Aided Sample Preparation Provide Sufficient Method Linearity for Quantitative Plant Shotgun Proteomics?. Front. Plant Sci..

[136] Frolov A., Shumilina J., Etemadi Afshar S., Mashkina V., Rhomanovskaya E., Lukasheva E., Tsarev A., Sulima A.S., Shtark O.Y., Ihling C. (2025). Responsivity of Two Pea Genotypes to the Symbiosis with Rhizobia and Arbuscular Mycorrhiza Fungi—A Proteomics Aspect of the “Efficiency of Interactions with Beneficial Soil Microorganisms” Trait. Int. J. Mol. Sci..

[137] Perez-Riverol Y., Bandla C., Kundu D.J., Kamatchinathan S., Bai J., Hewapathirana S., John N.S., Prakash A., Walzer M., Wang S. (2025). The PRIDE database at 20 years: 2025 update. Nucleic Acids Res..

