# Supplementary material for: Metabolic Responses to the Zinc Stress in the Roots and Leaves of Amaranthus caudatus: The Proteomics View
Source: Plants (Basel). 2025 Oct 30;14(21):3315. doi: 10.3390/plants14213315 (PMC12609541; doi:10.3390/plants14213315)
Supplement: Supplementary file 1 [file plants-14-03315-s001.zip › Supplementary Information 1_ revised_(without_color_marks).pdf]

Metabolic responses to the zinc stress in the roots and leaves of *Amaranthus caudatus*: the proteomics view

Anastasia Gurina,[1] Tatiana Bilova[1,2*], Daria Gorbach[2] Alena Soboleva,[2] Nataliia Stepanova,[3] Olga Babich,[4] Cristian Ihling,[5] Anastasia Kamionskaya,[3] Natalia Osmolovskaya[1] and Andrej Frolov[2*]

Supplementary Information 1

[1]Department of Plant Physiology and Biochemistry, St. Petersburg State University, 199034 St. Petersburg, Russia;

[2]Laboratory of Analytical Biochemistry and Biotechnology, K.A. Timiryazev Institute of Plant Physiology of the Russian Academy of Science, 127276 Moscow, Russia**;**

[3]FSI Federal Research Centre Fundamentals of Biotechnology of the Russian Academy of Science, 119071 Moscow, Russia;

[4]REC “Applied Biotechnologies”, Immanuel Kant Baltic Federal University, 236041 Kaliningrad, Russia;

[5] Department of Pharmaceutical Chemistry and Bioanalytics, Institute of Pharmacy, Martin-Luther Universität Halle-Wittenberg, 06120 Halle, Germany.

*Corresponding authors:

Dr. Andrej Frolov

K.A. Timiryazev Institute of Plant Physiology RAS

Laboratory of Analytical Biochemistry and Biotechnology

Botanicheskaya Ulitsa, 35

Moscow, 127276

Tel. +7 (499) 6785400

Email: analbiochem@ifr.moscow

Dr. Tatiana Bilova

K.A. Timiryazev Institute of Plant Physiology RAS

Laboratory of Analytical Biochemistry and Biotechnology

Botanicheskaya Ulitsa, 35

Moscow, 127276

Tel. +7 (499) 6785400

Email: bilova.tatiana@gmail.com

Directory

Material and reagents …………………………………………………………………………………5

Protocols

Protocol S1-1. Composition of nutrient solution ………………………………………………………..6

Protocol S1-1 Tryptic digestion of the total protein isolated from amaranth leaves and roots………………6

Tables

Table S1-1 Protein concentrations, protein recoveries and normalized lane densities acquired by SDS-PAGE densitometric analysis of individual samples of protein extracts isolated from the amaranth plants………...7

Table S1-2 Eigenvalues (loadings) obtained in the principal component analysis for the top-20 major contributors, in particular PC1, of differentially expressed part of amaranth proteome of the roots, young and mature leaves with or without Zn stress application …………………………………………………………9

Table S1-3 Eigenvalues (loadings) obtained in the principal component analysis for the top-20 major contributors, in particular PC1, of differentially expressed part of amaranth proteome of the roots, young and mature leaves without (control) Zn stress application ………………………………………………………11

Table S1-4 Eigenvalues (loadings) obtained in the principal component analysis for the top-20 major contributors, in particular PC1, of differentially expressed part of amaranth proteome of the roots, young and mature leaves with Zn stress application ……………………………………………………………………13

Table S1-5 Eigenvalues (loadings) obtained in the principal component analysis for the top-10 up- and down-accumulated entries, in particular PC1, of differentially expressed part of amaranth root proteome in response to Zn treatment ……………….……………………………………………………………………...….15

Table S1-6 28 functional classes (BINs) that could be annotated with Mercator MapMan software……..…17

Table S1-7 Eigenvalues (loadings) obtained in the principal component analysis for the top-10 up- and down-accumulated entries, in particular PC1, of differentially expressed part of amaranth young leaf proteome in response to Zn treatment ……………………………………………………………………………………..18

Table S1-8 Eigenvalues (loadings) obtained in the principal component analysis for the top up- and down-accumulated entries, in particular PC1, of differentially expressed part of amaranth mature leaf proteome in response to Zn treatment ……………………………………………………………………………………..20

Table S1-9 Parameters of the nanoHPLC separation method applied for ESI-Orbitrap-MS DDA experiments ………………………………………………………………………………………..……………21

Table S1-10 Instrument settings applied for ESI-LIT-Orbitrap-MS DDA experiments……………………22

Table S1-11 The settings of Proteome Discoverer 2.2 and QIProgenesis for the database search and label-free relative quantification………………………………………………………………………………….23

Figures

Figure S1-1 Shoots of seven week-old *A.caudatus* plants grown in the first independent experiment in hydroponic nutrient solution in the presence (Zn^{2+}-treated group) and absence (Control group) of 300 μmol/L $ZnSO_4$ for one week………………………………………………………………………………………24

Figure S1-2 Impact of exogenous Zn^{2+} (300 μmol/L) on stomatal conductivity, chlorophyll content and photosystem II activity of the third mature leaf of control and Zn treated plants ……………………………25

Figure S1-3 Leaf related water content of *A. caudatus* young and mature leaves of control and Zn treated plants of the 1st and 2nd plant experiments………………………………………………………………26

Figure S1-4 Shoots of seven week-old *A.caudatus* plants grown in the second independent experiment in hydroponic nutrient solution in the presence (Zn^{2+}-treated group) and absence (Control group) of 300 μmol/L $ZnSO_4$ for one week………………………………………………………………………………………27

Figure S1-5 Electropherograms of total protein extracts and corresponding filtrates from the amaranth leaves and roots under normal (sufficient water) growing conditions ………………………………………………28

Figure S1-6 Electropherograms of total protein extracts and corresponding filtrates from the amaranth leaves and roots under Zn stress conditions ……………………………………………………………………………29.

Figure S1-7 Numbers of protein groups, peptides and possible proteins identified in <u>roots, young and mature leaves</u> of the amaranth plants grown without Zn treatment …………………………………………………30

Figure S1-8 Numbers of protein groups, peptides and possible proteins identified in <u>roots, young and mature leaves</u> of the amaranth plants grown under Zn treatment ……………………………………………………31

Figure S1-9 Numbers of protein groups, proteins and peptides identified in <u>roots</u> of amaranth plants grown with (Stress) or without (Control) Zn treatment …………………………………………………………….31

Figure S1-10 Numbers of protein groups, proteins and peptides identified in <u>young leaves</u> of amaranth plants grown with (Stress) or without (Control) Zn treatment …………………………………………………….31

Figure S1-11 Numbers of protein groups, proteins and peptides identified in mature leaves of amaranth plants grown with (Stress) or without (Control) Zn treatment…………………………………………..................32

Figure S1-12 Principal component analysis with a loadings plot performed for all the treatment groups (roots, young and mature leaves) with or without stress application ……………………………………………33

Figure S1-13 Hierarchical clustering with a heatmap representation showing expression patterns of individual samples performed for all groups of plants grown without Zn treatment and all groups of plants grown under Zn stress conditions. ………………………………………………………………………………34

Figure S1-14 Principal component analysis performed for the roots of amaranth plants with a scores plot and loadings plot showing individual contribution of variables in the observed differences……………………..35

Figure S1-15 Principal component analysis performed for the young leaves of amaranth plants with a scores plot and loadings plot showing individual contribution of variables in the observed differences …………….36

Figure S1-16 Principal component analysis performed for the mature leaves of amaranth plants with a scores plot and loadings plot showing individual contribution of variables in the observed differences …………..37

Figure S1-17 Hierarchical clustering with a heatmap representation showing expression patterns in individual samples of the amaranth roots ……………………………………………………………………….38

Figure S1-18 Hierarchical clustering with a heatmap representation showing expression patterns in individual samples of the amaranth young leaves ………………………………..……………………………..39

Figure S1-19 Hierarchical clustering with a heatmap representation showing expression patterns in individual samples of the amaranth mature leaves ……………………………………………………………….39

Materials and reagents

Unless stated otherwise, materials were obtained from the following manufacturers. AMRESCO LLC (Cleveland, Ohio, USA): bis-acrylamide (ultra pure grade), phenylmethyl sulfonyl fluoride (high purity grade); Bioanalytical Technologies 3M Company (Saint Paul, Minnesota, USA): Empore™ solid phase octodecyl extraction discs; Calbiochem (Madison, Wisconsin, USA): glycine (molecular biology grade); Dia-M (Moscow, Russia): phenol for molecular biology (99.5 %); Ekos-1 (Moscow, Russia): acetone (extra pure); Helicon (Moscow, Russia): ammonium persulfate (ACS grade), sodium dodecyl sulfate (biotechnology grade), acrylamide 2K (standard grade, extra pure), Urea (USP grade, DL-dithiothreitol (biotechnology grade), potassium chloride (CP); LiChrosolv (Merck KGaA, Darmstadt, Germany): acetonitrile (HPLC grade); Merck KGaA (Darmstadt, Germany): acetonitrile (LC-MS grade), methanol (LC-MS grade); PanReac AppliChem (Darmstadt, Germany): 2-propanol (for HPLC, ≥ 99.9 %); Reachem (Moscow, Russia): hydrochloric acid (GR for analysis); SERVA Electrophoresis GmbH (Heidelberg, Germany): Coomassie Brilliant Blue G-250 (ultra-pure), 2-mercaptoethanol (research grade), trypsin NB (sequencing grade, modified from porcine pancreas); Santa Cruz Biotechnology (Santa Cruz, USA): BCA protein assay kit; Sigma-Aldrich (Saint Louis, Missouri, USA): ethylenediaminetetraacetic acid, iodoacetamide (BioUltra), ammonium bicarbonate (≥ 99.0%), formic acid (≥ 98%), ammonium acetate (≥ 98%); Thermo Fisher Scientific (Waltham, Massachusetts, USA): PageRullerTM Plus Prestained Protein Ladder #26619 (10–250 kDa); Dichrom GmbH (Marl, Germany): Progenta™ adaptors for stagetips. Water was purified in-house (resistance 5–15 mΩ/cm) on water conditioning and purification systems Elix 3 UV (Millipore, Moscow, Russia).

Protocols

Protocol S1-1. Composition of nutrient solution

The composition of full nutrient solution (in mmol/L) was as follows: $Ca(NO_3)_2{\times}4H_2O$ – 3.81; KNO_3 – 6.44; $MgSO_4{\times}7H_2O$ – 0.81; KH_2PO_4 – 1.83; NH_4NO_3 – 0.87; Fe-EDTA – 0.09; H_3BO_3 – 0.047; $MnSO_4{\times}5H_2O$ – 0.007; $ZnSO_4{\times}7H_2O$ – 0.0007; $CuSO_4{\cdot}5H_2O$ – 0.0008; $(NH_4)_2MoO_4$ – 0.0005. Nutrient solutions in the culturing vessels were continuously aerated and replaced every seven days. The pH values were daily monitored and maintained at 5.8±0.1. If necessary, the pH values were re-adjusted with 0.01 N H_2SO_4 or NaOH.

Protocol S1-2 Tryptic digestion of the total protein isolated from amaranth leaves and roots

The tryptic digestion of protein aliquots (25 µg) relied on the filter-aided sample preparation (FASP)-based protocol of Leonova et al [1] with minimal changes. In detail, Amicon Ultra 30K filter units (Merck KGaA, Darmstadt, Germany) were passivated overnight in 500 µL of 5% (*v/v*) Tween-20 (300 r.pm., 20 ^{o}C). The next day, filter units were washed three times with Milli-Q water (30 min, 300 r.p.m., 20 ^{o}C). Then, aliquots of protein isolates were applied and the sample volumes were adjusted to 200 µL with 8 mol/L urea in 50 mmol/L Tris-HCl buffer (Urea buffer, pH 8.0). Samples were centrifuged three times after washing with the same solution (10 min, 14 000g, 4°C) and afterwards protein disulfide bonds were reduced with 100 mmol/L DTT in urea buffer while shaking (1 h, 450 r.p.m., 4 ^{o}C) with subsequent centrifugation (10 min, 14 000g, 4 ^{o}C). The resulted sulfhydryls were alkylated with 50 mmol/L IAA in urea buffer (1 h, 450 r.p.m., 4 ^{o}C) and samples were centrifuged as above. Thereafter, filters were washed with urea buffer three times (10 min, 14 000g, 4 °C). Buffer exchange was performed with 50 mmol/L NH_4HCO_3 (ABC buffer) in water centrifugating each time (10 min, 14 000g, 4 °C) followed by digestion with trypsin in the same buffer in the enzyme:protein ratio 1:20 (4 h, 450 r.p.m., 37 ^{o}C) and 1:50 (14 h, 450 r.p.m., 37 ^{o}C). To assess the completeness of protein digestion, flipped filter units were transferred to new polypropylene receiver tubes and washed twice with 50 µL ABC buffer with subsequent SDS-PAGE verification [1].

Tables

Table S1-1. Protein concentrations, protein recoveries and normalized lane densities acquired by SDS-PAGE densitometric analysis of individual samples of protein extracts isolated from the amaranth plants under normal or Zn-stress conditions

#	Sample	Sample weight, g	Protein concentration, mg/mL	Protein recovery, mg/g FW	Normalized lane densities[a]
**1**	YL1 (Control)	0.19806	1.31	0.66	562731
**2**	YL2 (Control)	0.196	1.16	0.59	567813
**3**	YL3 (Control)	0.20422	1.06	0.52	570688
**4**	YL4 (Control)	0.2041	1.35	0.66	543030
**5**	YL5 (Control)	0.20409	1.40	0.68	551883
**6**	YL6 (Control)	0.20315	1.36	0.67	556724
**7**	ML1 (Control)	0.2025	1.21	0.60	601293
**8**	ML2 (Control)	0.19987	1.28	0.64	593211
**9**	ML3 (Control)	0.1998	1.00	0.50	596759
**10**	ML4 (Control)	0.2047	0.96	0.47	568312
**11**	ML5 (Control)	0.19683	1.42	0.72	616555
**12**	ML6 (Control)	0.2035	1.29	0.63	603179
**13**	R1 (Control)	0.2504	0.33	0.13	479742
**14**	R2 (Control)	0.25128	0.76	0.30	501613
**15**	R3 (Control)	0.2474	0.35	0.14	507458
**16**	R4 (Control)	0.246	0.42	0.17	499998
**17**	R5 (Control)	0.2538	0.43	0.17	491962
**18**	R6 (Control)	0.24798	0.66	0.27	494432
**19**	YL1 (Stress)	0.2017	1.13	0.56	495395
**20**	YL2 (Stress)	0.2048	1.00	0.49	513442
**21**	YL3 (Stress)	0.19819	1.11	0.56	507056
**22**	YL4 (Stress)	0.20472	0.65	0.32	514736
**23**	YL5 (Stress)	0.19848	1.08	0.54	505105
**24**	YL6 (Stress)	0.1972	1.27	0.65	503788
**25**	ML1 (Stress)	0.19916	0.91	0.46	394707
**26**	ML2 (Stress)	0.20315	1.10	0.54	381076
**27**	ML3 (Stress)	0.19838	1.06	0.53	363613

**28**	ML4 (Stress)	0.1971	1.24	0.63	391763
**29**	ML5 (Stress)	0.2035	1.05	0.52	417400
**30**	ML6 (Stress)	0.20179	1.11	0.55	412301
**31**	R1 (Stress)	0.2494	0.61	0.24	492302
**32**	R2 (Stress)	0.24907	0.94	0.38	559503
**33**	R3 (Stress)	0.2488	0.78	0.31	559571
**34**	R4 (Stress)	0.2517	1.02	0.41	485310
**35**	R5 (Stress)	0.24582	0.56	0.23	408627
**36**	R6 (Stress)	0.2546	0.73	0.29	493901

[a] optical densities of individual SDS-PAGE lanes. YL – young leaves, ML – mature leaves, R – roots.

Table S1-2. Eigenvalues (loadings) obtained in the principal component analysis for the top 20 major contributors, in particular PC1, of differentially expressed part of amaranth proteome of the roots, young and mature leaves with (Zn) or without (control) Zn stress application.

Protein name[a]	PC1[b]	FC[c]	*p*[d]	Highest mean condition	Lowest mean condition
germin-like protein	2.64	157.7	0.00E+00	roots_Zn	ML_control
LOW QUALITY PROTEIN: UPF0603 protein At1g54780, chloroplastic-like	2.57	1461.8	0.00E+00	ML_control	roots_control
putative pentatricopeptide repeat-containing protein At1g19290	2.04	179.0	4.93E-14	ML_control	roots_Zn
PsbE (chloroplast)	1.98	183.3	0.00E+00	ML_control	roots_Zn
thioredoxin M-type, chloroplastic-like	1.84	117.1	0.00E+00	ML_control	roots_Zn
ribulose-1,5-bisphosphate carboxylase/oxygenase large subunit (chloroplast)	1.82	94.1	0.00E+00	ML_control	roots_Zn
linoleate 13S-lipoxygenase 2-1, chloroplastic-like	1.72	119.8	0.00E+00	ML_Zn	roots_Zn
PsaA (chloroplast)	1.68	195.0	9.44E-15	ML_control	roots_control
glyceraldehyde-3-phosphate dehydrogenase B, chloroplastic	1.58	94.9	0.00E+00	ML_control	roots_Zn
purple acid phosphatase 22-like	1.55	59.1	4.00E-15	roots_control	YL_control
endochitinase At2g43590-like	1.52	55.8	0.00E+00	roots_Zn	YL_Zn
ATP-dependent DNA helicase PIF1-like	1.40	53.0	0.00E+00	ML_control	roots_control
50S ribosomal protein L12, chloroplastic-like	1.34	51.9	0.00E+00	YL_Zn	roots_Zn

protein CURVATURE THYLAKOID 1A, chloroplastic-like	1.30	68.7	0.00E+00	ML_Zn	roots_control
PsbA (chloroplast)	1.28	85.8	0.00E+00	ML_control	roots_control
ferredoxin--NADP reductase, chloroplastic-like	1.20	57.9	0.00E+00	ML_control	roots_Zn
fructose-bisphosphate aldolase 1, chloroplastic	1.19	92.7	0.00E+00	ML_control	roots_Zn
peroxidase 3-like	1.18	62.5	0.00E+00	roots_control	ML_Zn
oxygen-evolving enhancer protein 1, chloroplastic-like	1.14	56.1	0.00E+00	ML_control	roots_control
PsbF (chloroplast)	1.13	68.1	0.00E+00	ML_control	roots_Zn

[a]Identification of peptides and annotation of protein relied on a search against amino acid sequences of *Chenopodium quinoa* (uploaded from KEGG protein database) accomplished with SEQUEST algorithm, Mercator v4.5 was used for proteins annotation, subcellular localization of proteins was defined using BUSCA; [b]PC, principal component; [c]FC, fold change; [d]*p*, *p*-value; YL – young leaves, ML – mature leaves.

Table S1-3. Eigenvalues (loadings) obtained in the principal component analysis for the top-20 major contributors, in particular PC1, of differentially expressed part of amaranth proteome of the roots, young and mature leaves without (control) Zn stress application.

Protein name[a]	PC1[b]	FC[c]	*p*[d]	Highest mean condition	Lowest mean condition
uncharacterized protein LOC110707738 isoform X1	2.11	Infinity	0.00E+00	ML_control	roots_control
LOW QUALITY PROTEIN: UPF0603 protein At1g54780, chloroplastic-like	2.09	1461.8	1.30E-12	ML_control	roots_control
probable glutathione S-transferase parC isoform X1	2.04	Infinity	0.00E+00	ML_control	roots_control
chlorophyll a-b binding protein, chloroplastic	1.79	203.5	1.16E-13	ML_control	roots_control
germin-like protein	1.78	72.9	3.46E-13	roots_control	ML_control
PsaA (chloroplast)	1.67	244.8	2.81E-12	ML_control	roots_control
putative pentatricopeptide repeat-containing protein At1g19290	1.65	155.4	1.83E-14	ML_control	roots_control
PsbE (chloroplast)	1.62	162.9	1.18E-11	ML_control	roots_control
peroxidase 3-like	1.50	62.4	3.15E-11	roots_control	ML_control
PsaB (chloroplast)	1.47	185.5	5.04E-12	ML_control	roots_control
purple acid phosphatase 22-like	1.43	59.1	1.23E-08	roots_control	YL_control
ribulose-1,5-bisphosphate carboxylase/oxygenase large subunit (chloroplast)	1.42	102.4	2.89E-15	ML_control	roots_control

thioredoxin M-type, chloroplastic-like	1.34	103.3	4.85E-11	ML_control	roots_control
PsbA (chloroplast)	1.30	115.1	8.29E-14	ML_control	roots_control
elongation factor TuB, chloroplastic-like	1.27	62.1	7.28E-13	ML_control	roots_control
glyceraldehyde-3-phosphate dehydrogenase B, chloroplastic	1.24	95.9	0.00E+00	ML_control	roots_control
PetB (chloroplast)	1.24	90.9	1.78E-09	ML_control	roots_control
MLP-like protein 31	1.18	50.1	9.32E-10	roots_control	ML_control
endochitinase At2g43590-like	1.11	43.3	1.76E-10	roots_control	YL_control
protein CURVATURE THYLAKOID 1A, chloroplastic-like	1.05	62.8	1.90E-14	ML_control	roots_control

[a]Identification of peptides and annotation of protein relied on a search against amino acid sequences of *Chenopodium quinoa* (uploaded from KEGG protein database) accomplished with SEQUEST algorithm, Mercator v4.5 was used for proteins annotation, subcellular localization of proteins was defined using BUSCA; [b]PC, principal component; [c]FC, fold change; [d]*p*, *p*-value; YL – young leaves, ML – mature leaves.

Table S1-4. Eigenvalues (loadings) obtained in the principal component analysis for the top 20 major contributors, in particular PC1, of differentially expressed part of amaranth proteome of the roots, young and mature leaves with Zn stress application.

**Protein name**[a]	**PC1**[b]	**FC**[c]	***p***[d]	**Highest mean condition**	**Lowest mean condition**
germin-like protein	2.59	129.8	2.20E-14	roots_Zn	ML_Zn
peroxidase 12-like	2.26	113.5	1.08E-09	roots_Zn	ML_Zn
NdhJ (chloroplast)	2.21	Infinity	0.00E+00	ML_Zn	roots_Zn
putative pentatricopeptide repeat-containing protein At1g19290	1.99	166.0	2.19E-06	ML_Zn	roots_Zn
LOW QUALITY PROTEIN: UPF0603 protein At1g54780, chloroplastic-like	1.67	214.4	7.45E-14	ML_Zn	roots_Zn
ribulose-1,5-bisphosphate carboxylase/oxygenase large subunit (chloroplast)	1.66	84.5	1.56E-13	ML_Zn	roots_Zn
linoleate 13S-lipoxygenase 2-1, chloroplastic-like	1.66	119.8	1.11E-16	ML_Zn	roots_Zn
bifunctional riboflavin biosynthesis protein RIBA 1, chloroplastic-like	1.65	58.8	2.24E-13	roots_Zn	ML_Zn
non-symbiotic hemoglobin 1-like	1.61	47.5	3.29E-10	roots_Zn	YL_Zn
PsbE (chloroplast)	1.59	140.2	7.39E-14	ML_Zn	roots_Zn
50S ribosomal protein L9, chloroplastic	1.56	96.0	7.88E-15	YL_Zn	roots_Zn
alcohol dehydrogenase 1	1.54	48.5	3.57E-12	roots_Zn	ML_Zn
glyceraldehyde-3-phosphate dehydrogenase B, chloroplastic	1.52	89.4	2.66E-15	ML_Zn	roots_Zn
thioredoxin M-type, chloroplastic-like	1.51	85.0	9.73E-12	ML_Zn	roots_Zn

probable inactive ATP-dependent zinc metalloprotease FTSHI 5, chloroplastic	1.31	66.7	1.41E-11	ML_Zn	roots_Zn
endochitinase At2g43590-like	1.30	55.8	4.21E-10	roots_Zn	YL_Zn
50S ribosomal protein L12, chloroplastic-like	1.28	51.9	1.97E-10	YL_Zn	roots_Zn
PsaA (chloroplast)	1.22	76.3	7.48E-09	ML_Zn	roots_Zn
purple acid phosphatase 22-like	1.13	35.2	6.83E-09	roots_Zn	YL_Zn
oxygen-evolving enhancer protein 2, chloroplastic	1.12	62.2	1.44E-12	ML_Zn	roots_Zn

[a]Identification of peptides and annotation of protein relied on a search against amino acid sequences of *Chenopodium quinoa* (uploaded from KEGG protein database) accomplished with SEQUEST algorithm, Mercator v4.5 was used for proteins annotation, subcellular localization of proteins was defined using BUSCA; [b]PC, principal component; [c]FC, fold change; [d]*p*, *p*-value; YL – young leaves, ML – mature leaves.

Table S1-5. Eigenvalues (loadings) obtained in the principal component analysis for the top-10 up- and down-accumulated entries, in particular PC1, of differentially expressed part of amaranth root proteome in response to Zn treatment.

**Protein name[a]**	**PC1[b]**	***p*[d]**	**FC[c]**
Proteins displaying a higher abundance under Zn stress in comparison to control			
putative 12-oxophytodienoate reductase 11 isoform X1	0.15	7.32E-07	27.9
probable glutathione S-transferase parC	0.15	8.60E-06	39.4
chloroplast stem-loop binding protein of 41 kDa b, chloroplastic-like	0.09	1.85E-05	1.6
glutathione S-transferase-like	0.14	2.31E-05	10.6
probable inactive purple acid phosphatase 29	0.13	5.03E-05	10.6
probable aldo-keto reductase 4	0.15	5.03E-05	8.0
non-symbiotic hemoglobin 1-like	0.13	1.01E-04	14.9
mitochondrial uncoupling protein 4-like	0.12	1.95E-04	13.0
alcohol dehydrogenase 1	0.13	1.95E-04	9.8
bifunctional riboflavin biosynthesis protein RIBA 1, chloroplastic-like	0.13	2.29E-04	6.1
Proteins displaying a decreased abundance under Zn stress in comparison to control			
plasma membrane ATPase 4-like	-0.13	6.22E-05	2.0
plasma membrane ATPase 4-like isoform X1	-0.12	8.32E-05	1.9
tubulin alpha-3 chain	-0.11	1.40E-04	1.7
ABC transporter F family member 3-like	-0.14	1.95E-04	3.5
tubulin beta-5 chain-like	-0.12	3.47E-04	1.9
peroxidase 3-like	-0.14	3.68E-04	4.9
vacuolar-processing enzyme-like	-0.14	3.68E-04	2.1
aspartyl protease AED3-like	-0.15	3.97E-04	2.8

PetA (chloroplast)	-0.09	9.08E-04	1.5
glutamine synthetase, chloroplastic	-0.13	1.36E-03	2.5

[a]Identification of peptides and annotation of protein relied on a search against amino acid sequences of *Chenopodium quinoa* (uploaded from KEGG protein database) accomplished with SEQUEST algorithm, Mercator v4.5 was used for proteins annotation, subcellular localization of proteins was defined using BUSCA; [b]PC, principal component; [c]FC, fold change, for proteins that demonstrated an increase in abundance, FC(Zn stress/Control) indicates an (≥1.5-fold) increase in their abundance under stress Zn compared to the control, and for proteins that showed a decrease in abundance, FC(Control/Zn stress) shows a (≥1.5-fold) decrease in their abundance under Zn stress compared to the control. Thus, the FC values were adjusted for cases showing a decrease by presenting the inverse of the FC value; [d]*p*, *p*-value.

Table S1-6. 28 functional classes (BINs) that could be annonated with Mercator MapMan (v4.5) software.

№ Bincode	Function
bin1	Photosynthesis
bin2	Cellular respiration
bin3	Carbohydrate metabolism
bin4	Amino acid metabolism
bin5	Lipid metabolism
bin6	Nucleotide metabolism
bin7	Coensyme metabolism
bin8	Polyamine metabolism
bin9	Secondary metabolism
bin10	Redox homeostasis
bin11	Phytohormone action
bin12	Chromatin organisation
bin13	Cell cycle organisation
bin14	DNA damage response
bin15	RNA biosyntesis
bin16	RNA processing
bin17	Protein biosynthesis
bin18	Protein modification
bin19	Protein homeostasis
bin20	Cytoskeleton organisation
bin21	Cell wall organisation
bin22	Vesicle trafficing
bin23	Protein translocation
bin24	Solute transport
bin25	Nutrient uptake
bin26	External stimuli response
bin27	Multi-process regulation

Table S1-7. Eigenvalues (loadings) obtained in the principal component analysis for the top-10 up- and down-accumulated entries, in particular PC1, of differentially expressed part of amaranth young leaf proteome in response to Zn treatment.

**Protein name**[a]	**PC1**[b]	***p***[d]	**FC**[c]
Proteins displaying a higher abundance under Zn stress in comparison to control			
CBS domain-containing protein CBSX3, mitochondrial-like	0.22	9.01E-03	2.1
dihydropyrimidine dehydrogenase (NADP(+)), chloroplastic-like	0.19	9.01E-03	1.9
plasma membrane ATPase 4-like	0.21	1.07E-02	1.9
peroxiredoxin-2E-1, chloroplastic-like	0.17	1.23E-02	1.7
dihydrolipoyl dehydrogenase, mitochondrial	0.19	1.23E-02	1.5
receptor-like protein 12	0.21	2.55E-02	2.6
ferredoxin--NADP reductase, chloroplastic-like	0.20	2.55E-02	2.1
polygalacturonase inhibitor-like	0.20	2.55E-02	2.0
aminomethyltransferase, mitochondrial-like	0.20	2.78E-02	1.6
monodehydroascorbate reductase-like	0.18	2.86E-02	1.5
Proteins displaying a decreased abundance under Zn stress in comparison to control			
uncharacterized protein LOC110707738 isoform X1	-0.09	8.92E-03	1.6
60S ribosomal protein L10-like	-0.11	8.92E-03	1.6
60S ribosomal protein L4-like	-0.10	1.01E-02	2.1
magnesium-protoporphyrin IX monomethyl ester [oxidative] cyclase, chloroplastic-like	-0.21	1.60E-02	3.0
PsbC (chloroplast)	-0.11	2.55E-02	2.6
translocase of chloroplast 159, chloroplastic-like	-0.20	2.55E-02	1.9
elongation factor TuB, chloroplastic-like	-0.07	2.55E-02	1.6

elongation factor 2-like	-0.13	2.55E-02	1.6
60S ribosomal protein L13-1-like	-0.11	2.55E-02	1.5
porphobilinogen deaminase, chloroplastic-like	-0.12	2.75E-02	2.0

[a]Identification of peptides and annotation of protein relied on a search against amino acid sequences of *Chenopodium quinoa* (uploaded from KEGG protein database) accomplished with SEQUEST algorithm, Mercator v4.5 was used for proteins annotation, subcellular localization of proteins was defined using BUSCA; [b]PC, principal component; [c]FC, fold change, for proteins that demonstrated an increase in abundance, FC(Zn stress/Control) indicates an (≥1.5-fold) increase in their abundance under stress Zn compared to the control, and for proteins that showed a decrease in abundance under Zn stress compared to the control, the FC values were adjusted by presenting the inverse of the FC value as FC(Control/Zn stress, ≥1.5); [d]*p*, *p*-value.

Table S1-8. Eigenvalues (loadings) obtained in the principal component analysis for the top up- and down-accumulated entries, in particular PC1, of differentially expressed part of amaranth mature leaf proteome in response to Zn treatment.

Protein name[a]	PC1[b]	*p*[d]	FC[c]
Proteins displaying a higher abundance under Zn stress in comparison to control			
heat shock protein 83	0.14	2.91E-04	4.3
probable glutathione S-transferase parC	0.20	2.91E-04	2.1
betaine aldehyde dehydrogenase, chloroplastic	0.22	1.24E-03	1.6
annexin D2-like	0.18	7.93E-03	2.1
citrate synthase, mitochondrial-like	0.22	7.93E-03	1.5
uncharacterized protein LOC110692268	0.18	1.54E-02	2.0
LOW QUALITY PROTEIN: urease-like	0.22	2.32E-02	1.7
luminal-binding protein-like	0.20	2.32E-02	1.5
Proteins displaying a decreased abundance under Zn stress in comparison to control			
PetA (chloroplast)	0.21	3.25E-03	2.1
catalase	0.27	1.54E-02	1.6
photosystem II 10 kDa polypeptide, chloroplastic	0.24	1.75E-02	1.9
LRR receptor-like serine/threonine-protein kinase GSO1	0.26	2.12E-02	1.6
50S ribosomal protein L9, chloroplastic	0.24	2.32E-02	1.6
cytochrome b6-f complex iron-sulfur subunit, chloroplastic	0.25	2.32E-02	1.6
magnesium-chelatase subunit ChlI, chloroplastic-like	0.23	2.98E-02	1.7
NdhA (chloroplast)	0.24	2.98E-02	1.6
callose synthase 10-like	0.15	3.22E-02	2.6
NdhH (chloroplast)	0.20	3.22E-02	1.9
PsaA (chloroplast)	0.25	3.42E-02	1.5
ribosomal protein S14 (chloroplast)	0.23	3.94E-02	1.6
uncharacterized protein LOC110707738 isoform X1	0.21	4.68E-02	1.6

[a]Identification of peptides and annotation of protein relied on a search against amino acid sequences of *Chenopodium quinoa* (uploaded from KEGG protein database) accomplished with SEQUEST algorithm, Mercator v4.5 was used for proteins annotation, subcellular localization of proteins was defined using BUSCA; [b]PC, principal component; [c]FC, fold change, for proteins that demonstrated an increase in abundance, FC(Zn stress/Control) indicates an (≥1.5-fold) increase

in their abundance under stress Zn compared to the control, and for proteins that showed a decrease in abundance under Zn stress compared to the control, the FC values were adjusted by presenting the inverse of the FC value as FC(Control/Zn stress, ≥1.5); [d]*p*, *p*-value.

Table S1-9. Parameters of the nanoHPLC separation method applied for ESI-Orbitrap-MS DDA experiments

Parameter	Setting
Injection volume	10 µL
Sample pickup flow	30 µL/min
Injection mode	Full loop injection
Wash of the trap column	15 min with 0.1% (*v/v*) TFA at a flow rate of 30 µL/min
Eluents	A: 0.1% (*v/v*) aq. formic acid; B: 0.1% (*v/v*) formic acid in acetonitrile
Elution flow rate	0.3 µL/min
Column temperature	40ºC
Elution regimen	Linear gradient – from 3 to 40% eluent B in 45 min
	Linear gradient – from 40 to 99% eluent B in 5 min
	Isocratic at 99% eluent B during 5 min
	Linear gradient – from 99 to 3% eluent B in 5 min
	Isocratic at 3% eluent B during 15 min

Table S1-10. Instrument settings applied for ESI-LIT-Orbitrap-MS DDA experiments

Parameter	Setting
MS conditions	
Ionization mode	Positive ion mode
Resolution	60 000 at *m/z* 200
Ion spray voltage (IS)	1900 V
Capillary temperature	275 °C
Default charge state	2
Microscans	1
AGC Target	2×10^{5}
Maximum trap injection time (IT)	50 ms
Number of scan ranges	1
Mass to charge ratio (*m/z*) range	300 – 1500
MS/MS conditions	
Fragmentation type	Collision induced dissociation (CID)
Resolution	15 000
AGC target	5×10^{4}
Maximum IT	200 ms
TopN	5
Isolation window	1.5 Th
NCE	35 %
Intensity threshold	3×10^{4}
Charge state rejected	Unassigned, $+1 < n < +7$
Peptide match	Preferred
Exclude isotopes	On
Duration of dynamic exclusion duration	60 s

Table S1-11. The settings of Proteome Discoverer 2.2 and QIProgenesis for the database search and label-free relative quantification

**QIProgenesis**	
Max. fold change	≤ 1.5
ANOVA *p*-value	≤ 0.05
q-value	≤ 0.05
rank	>3
CV	≤ 60.5
**Proteome Discoverer 2.2**	
Parent mass error tolerance	10 ppm
Fragment mass error tolerance	0.8 Da
Min.peptide length	6
Max. peptide length	144
Missed cleavage sites	3
Fixed modifications	Carbamidomethylation
Dynamic modifications	Oxidation
Peptide and protein level FDR	0.05

Figures

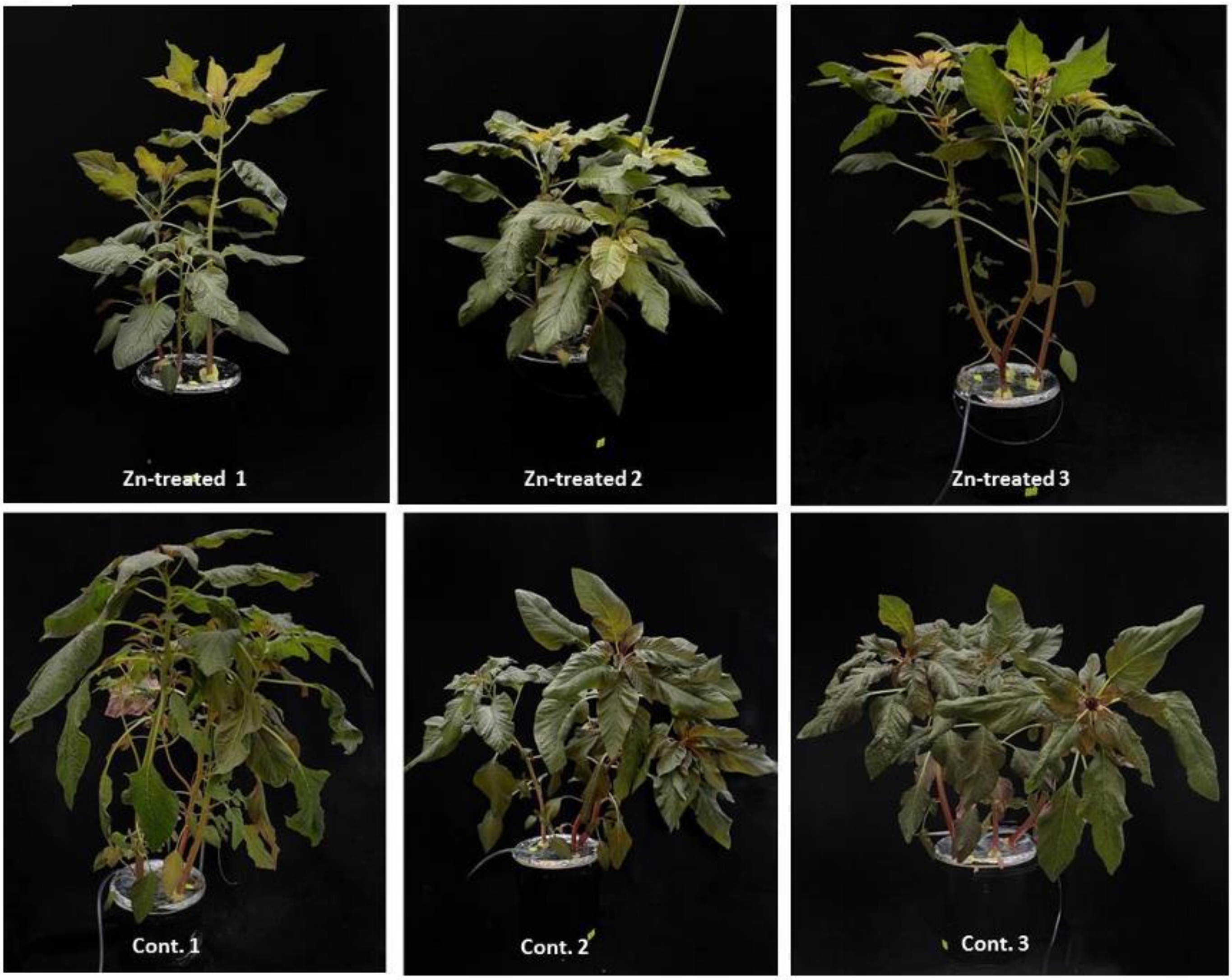

Figure S1-1 Shoots of seven week-old *A.caudatus* plants grown in the first independent experiment in hydroponic nutrient solution in the presence (Zn^{2+}-treated group, n = 9) and absence (Control group, n = 9) of 300 μmol/L $ZnSO_4$ for one week. Each photo presents three plants grown in a vessel. There were three vessels in control and Zn^{2+}-treated groups. This figure was originally featured in Osmolovskaya et al. [2].

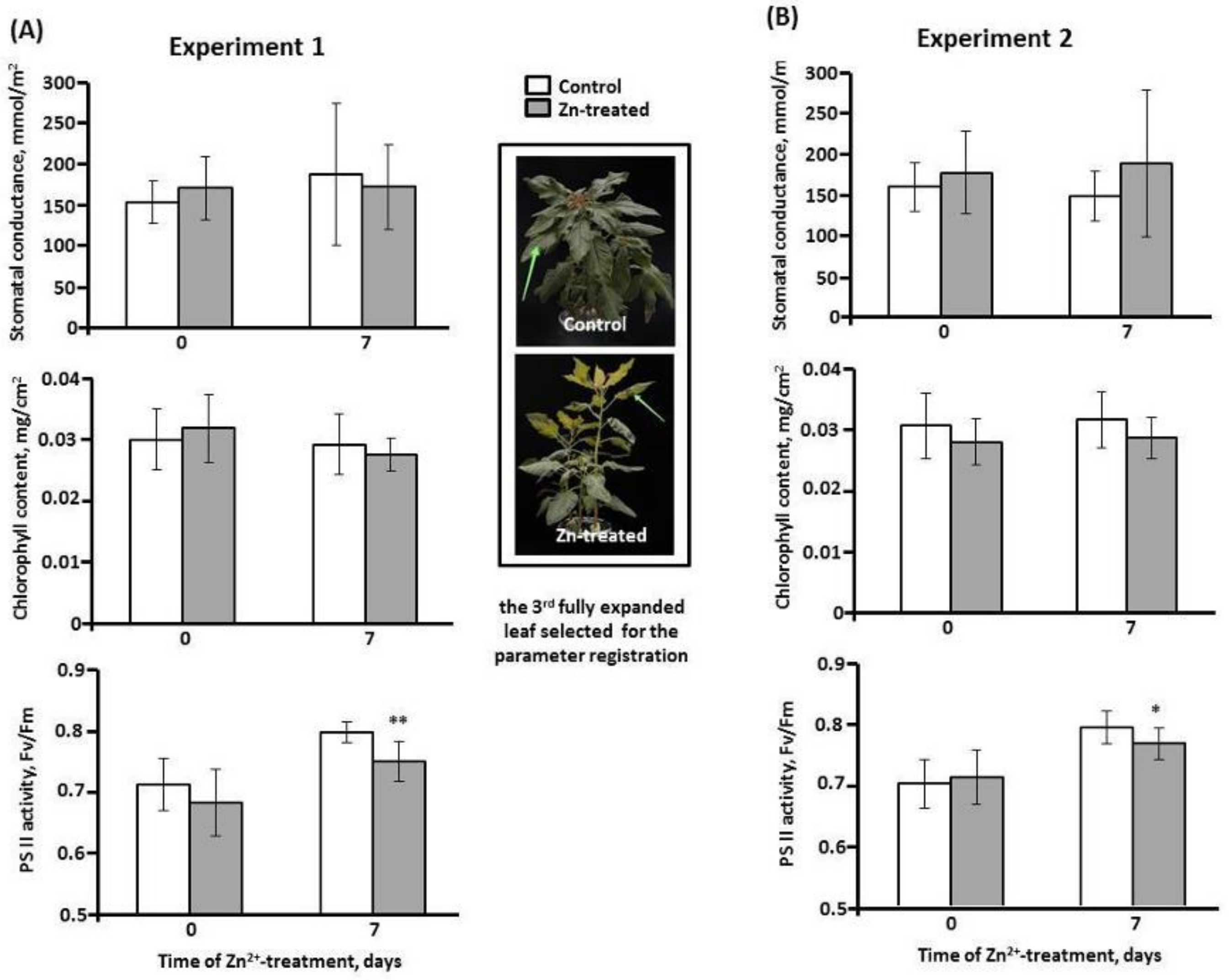

Figure S1-2. Impact of exogenous Zn^{2+} (300 μmol/L) on stomatal conductivity, chlorophyll content and photosystem II (PS II) activity of the third mature leaf (the leaf is indicated by green arrows at photos in the insert; numeration of the mature leaves were done just beneath the plant top with young (i.e. not fully expanded) leaves) from six week-old (before Zn2+-exposure, 0 day) and seven week-old *A.caudatus* plants (n = 9) (after Zn^{2+}-exposure, 7 day) grown in the first (A) and second (B) independent identical experiments in hydroponic nutrient solution. Grey columns indicate the Zn-treated group, namely plants which physiological parameters were measured prior (0 day) and after the Zn-stress exposure (7 day). The Zn-stress exposure of the plants was performed by supplementation of 300 μmol/L $ZnSO_4$ in hydroponic nutrient solution for one week. White columns indicate the control group of plants which were grown during the whole experimental period (from 0 to 7 days) in hydroponic nutrient solution without the addition of 300 μmol/L $ZnSO_4$. F_v/F_m – variable fluorescence (F_v)/maximal fluorescence (F_m). Asteriks * and ** indicate significant difference between Zn-treated and control groups per time point at *p*(*t*-test)<0.05 and <0.01, respectively. The experimental data obtained for a leaf of each plant are presented in Osmolovskaya et al. [2] Supplementary Information 2, Tables S2(1–3).

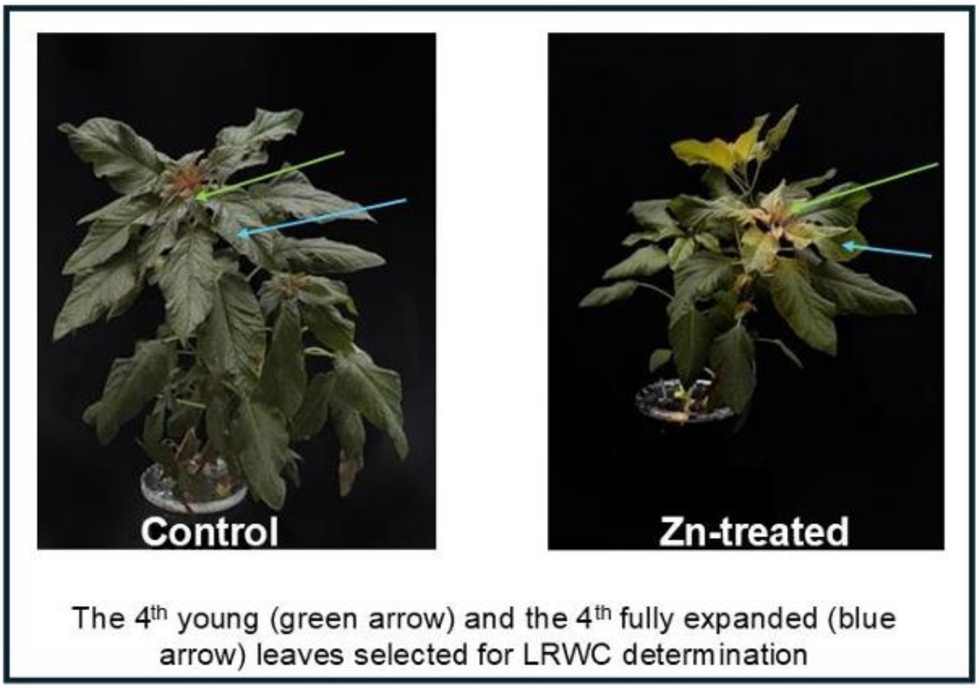

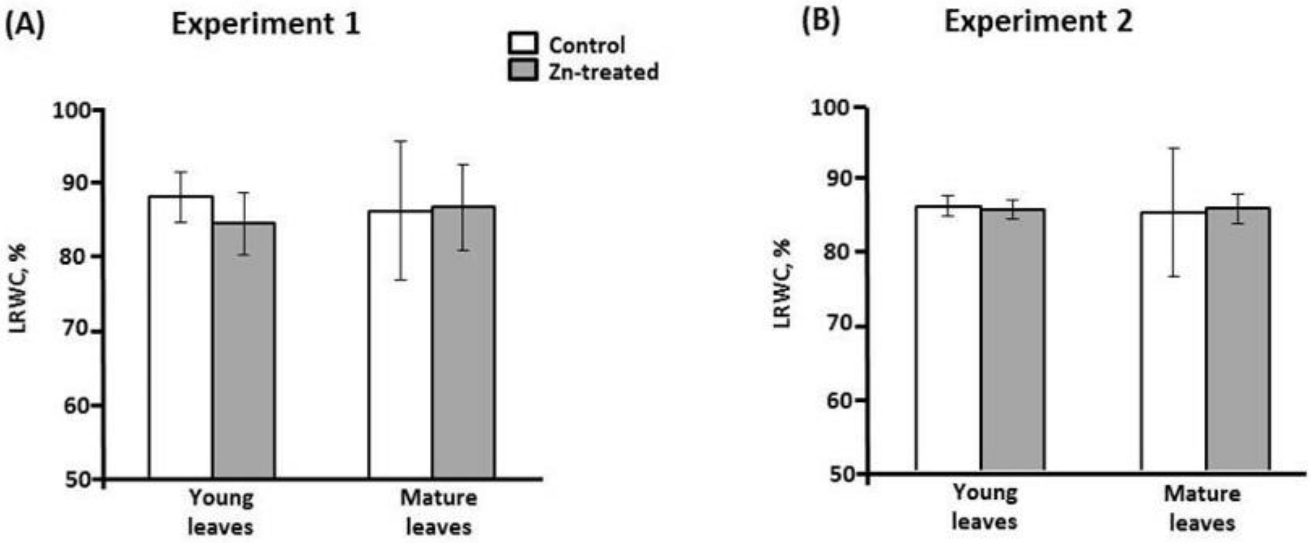

Figure S1-3. Leaf related water content (LRWC) of *A. caudatus* young and mature leaves of control and Zn treated plants of the 1st and 2nd plant experiments. In each experiment the 4th young and the 4th mature leaves of every plant (as indicated by green arrows at photos in the insert; numeration of the mature leaves were done just beneath the plant top with young (i.e. not fully expanded) leaves) were collected from seven week-old plants (n = 9) grown in hydroponic nutrient solution in presence (Zn^{2+}-treated group) and absence (control group) of 300 μmol/L $ZnSO_4$ for one week. The experimental data obtained for each plant are presented in Osmolovskaya et al. [2] Supplementary Information 2, Table S2(4).

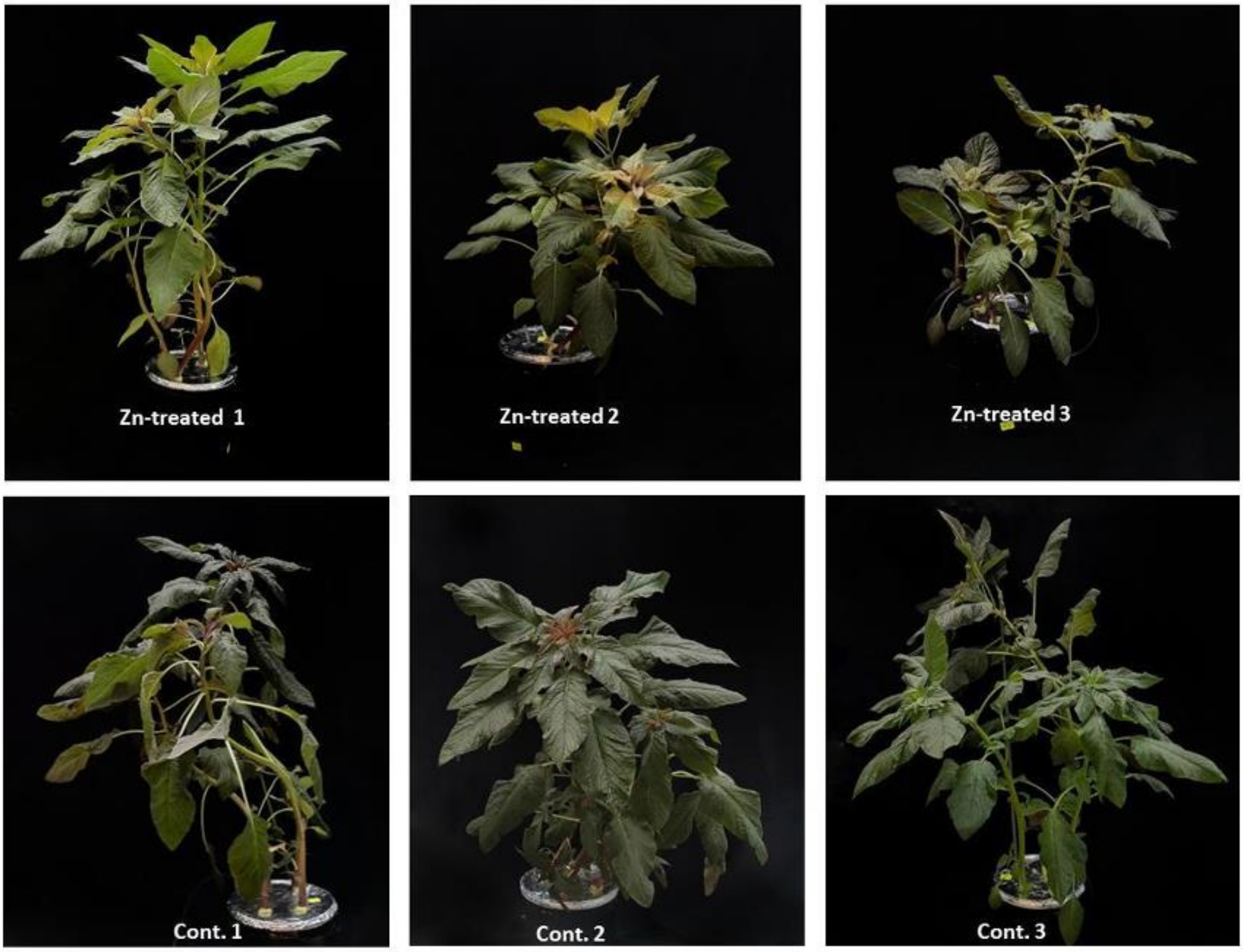

Figure S1-4 Shoots of seven week-old *A.caudatus* plants grown in the second independent experiment in hydroponic nutrient solution in the presence (Zn^{2+}-treated group, n = 9) and absence (Control group, n = 9) of 300 μmol/L $ZnSO_4$ for one week. Each photo presents three plants grown in a vessel. There were three vessels in control and Zn^{2+}-treated groups. This figure was originally featured in Osmolovskaya et al. [2].

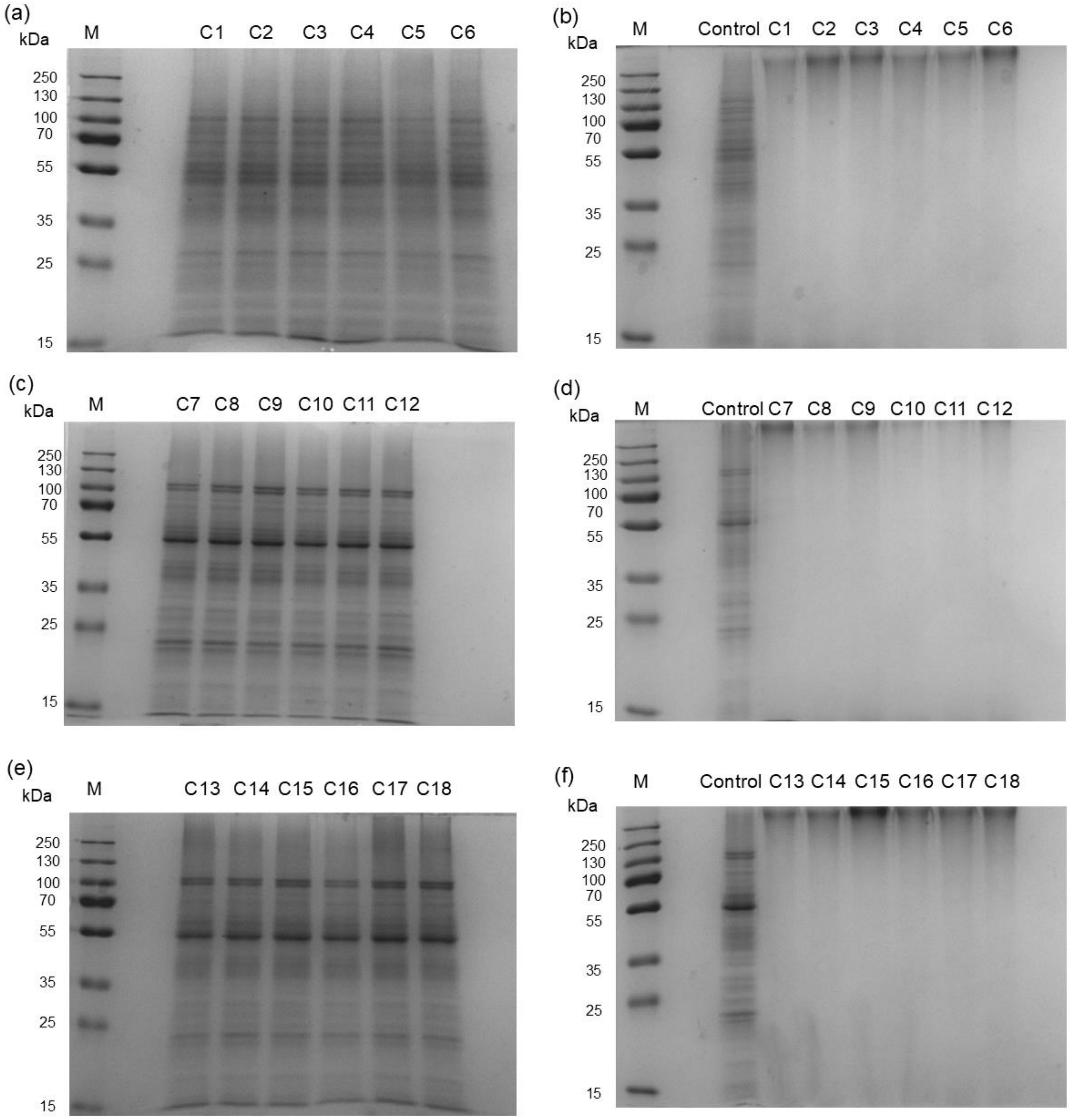

Figure S1-5. Electropherograms of total protein (a, c, e) extracts (5 μg per lane) and corresponding filtrates (b, d, f) from the amaranth leaves and roots under normal (sufficient water) growing conditions. M – marker of molecular weights (PageRuller Plus Prestained Protein Ladder, 10–250 kDa, Thermo Fisher Scientific), Control – total protein sample for digest control, 1-6 – samples obtained from young leaves; 7-12 – samples obtained from mature leaves; 13-18 - samples obtained from roots.

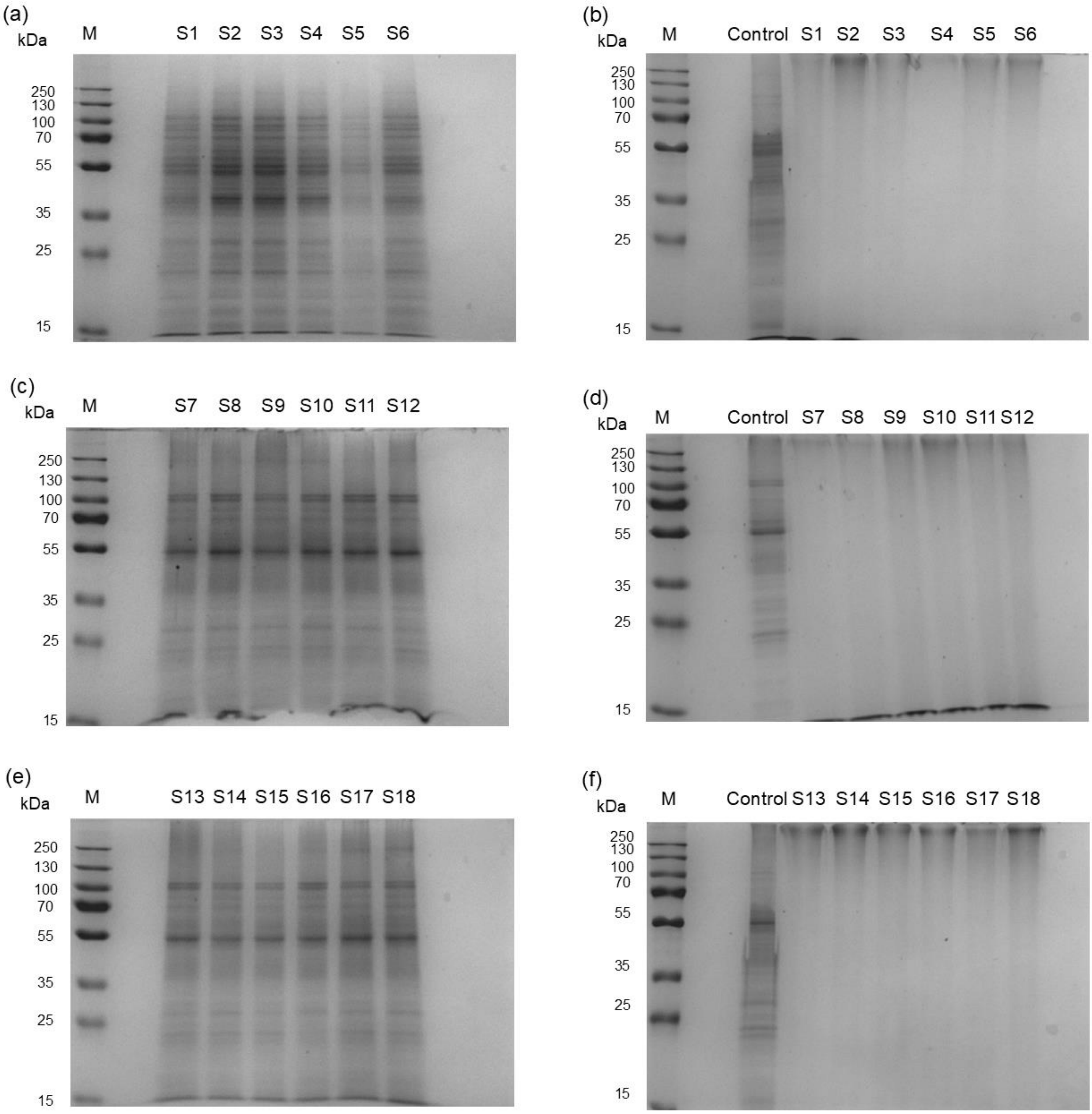

Figure S1-6 Electropherograms of total protein (a, c, e) extracts (5 μg per lane) and corresponding filtrates (b, d, f) from the amaranth leaves and roots under Zn stress conditions. M – marker of molecular weights (PageRuller Plus Prestained Protein Ladder, 10–250 kDa, Thermo Fisher Scientific), Control – total protein sample for digest control, 1-6 – samples obtained from young leaves; 7-12 – samples obtained from mature leaves; 13-18 – samples obtained from roots.

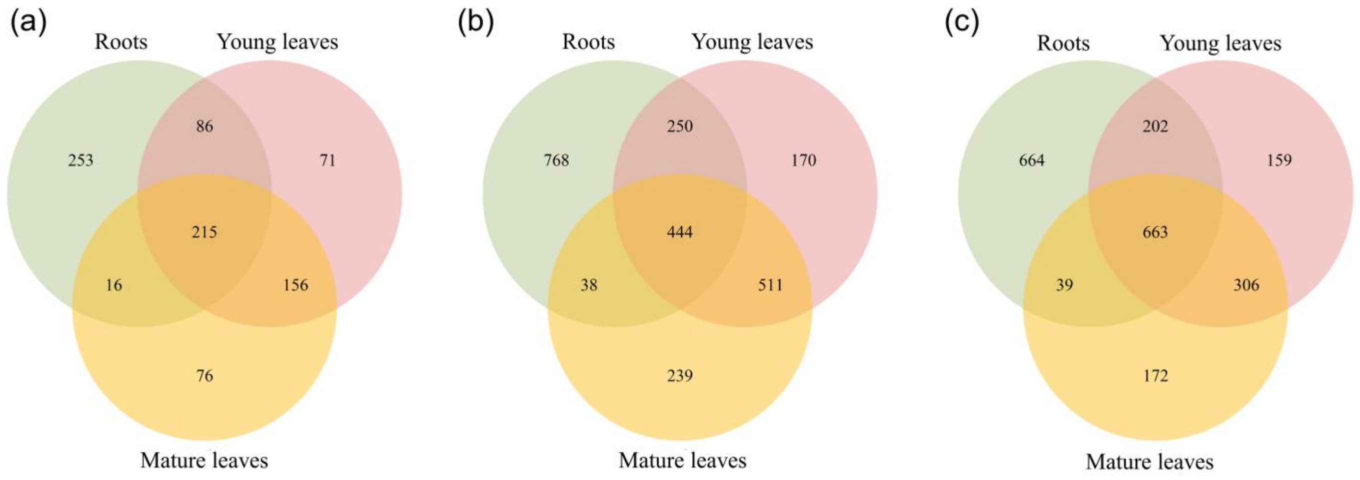

Figure S1-7. Numbers of protein groups (a), peptides (b) and possible proteins (c) identified in roots, young and mature leaves of the amaranth plants grown without Zn treatment (supplementation of 300 μmol/L Zn^{2+} to the hydroponic solution for 7 days.

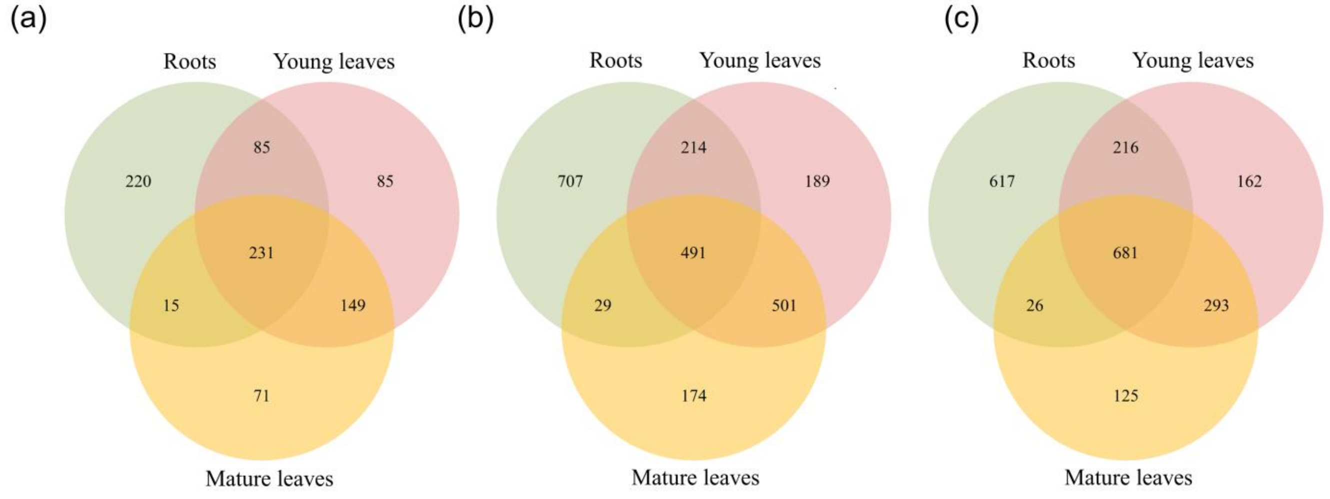

Figure S1-8. Numbers of protein groups (a), peptides (b) and possible proteins (c) identified in roots, young and mature leaves of the amaranth plants grown under Zn treatment (supplementation of 300 μmol/L Zn^{2+} to the hydroponic solution for 7 days).

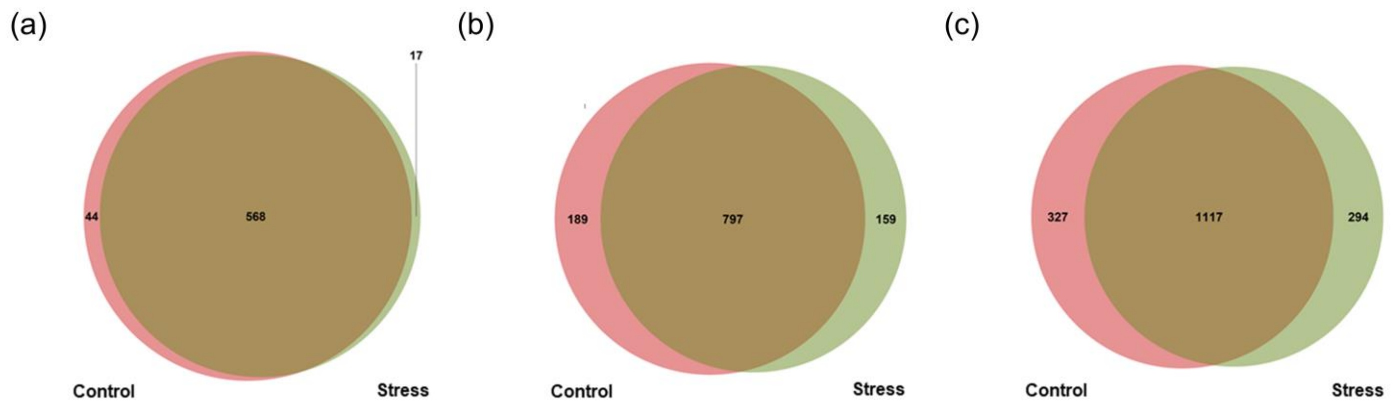

Figure S1-9. Numbers of protein groups (a), proteins (b) and peptides (c) identified in roots of amaranth plants grown with (Stress) or without (Control) Zn treatment (supplementation of 300 µmol/L Zn^{2+} to the hydroponic solution for 7 days).

.

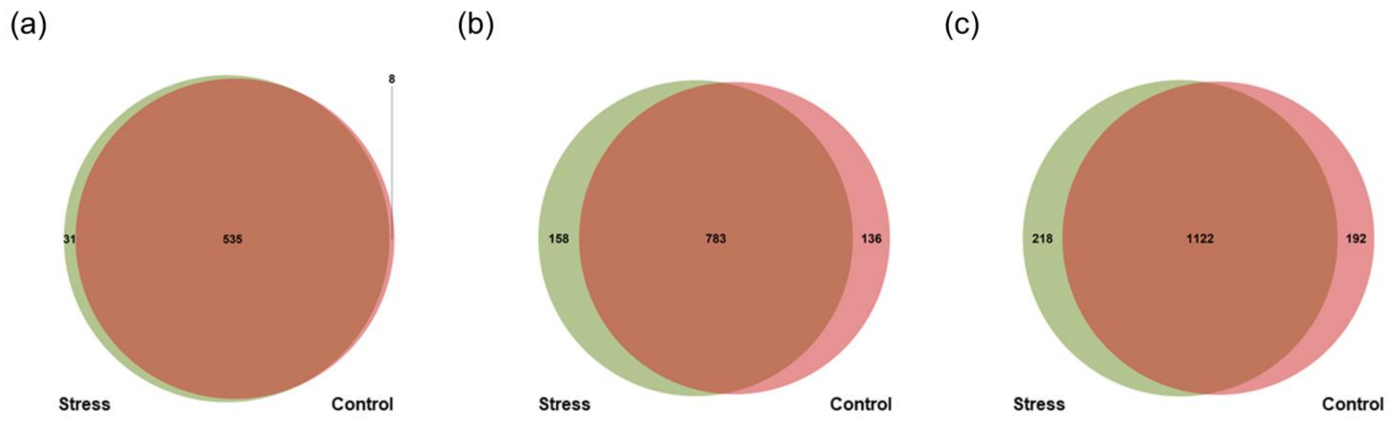

Figure S1-10. Numbers of protein groups (a), proteins (b) and peptides (c) identified in young leaves of amaranth plants grown with (Stress) or without (Control) Zn treatment (supplementation of 300 µmol/L Zn^{2+} to the hydroponic solution for 7 days).

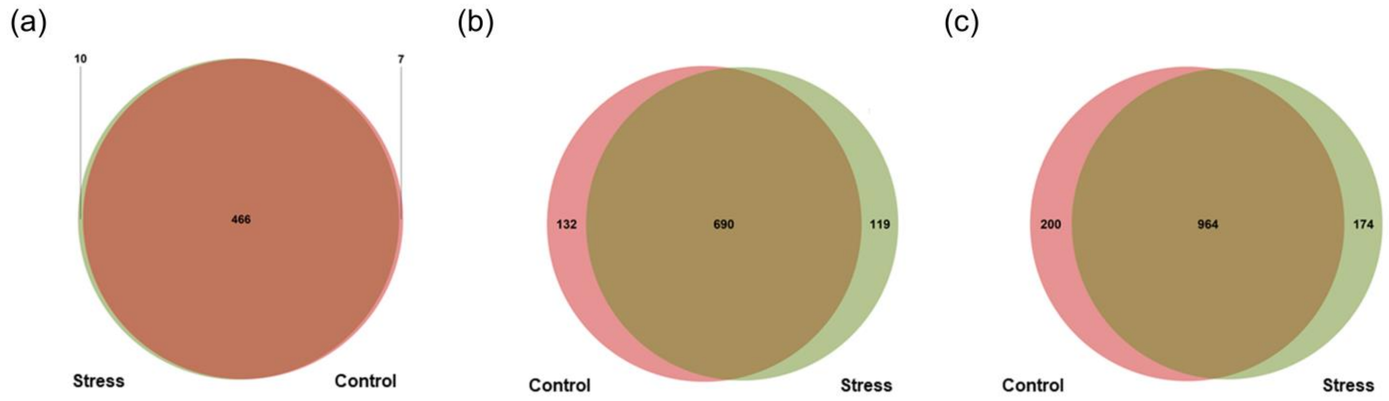

Figure S1-11. Numbers of protein groups (a), proteins (b) and peptides (c) identified in mature leaves of amaranth plants grown with (Stress) or without (Control) Zn treatment (supplementation of 300 μmol/L Zn^{2+} to the hydroponic solution for 7 days).

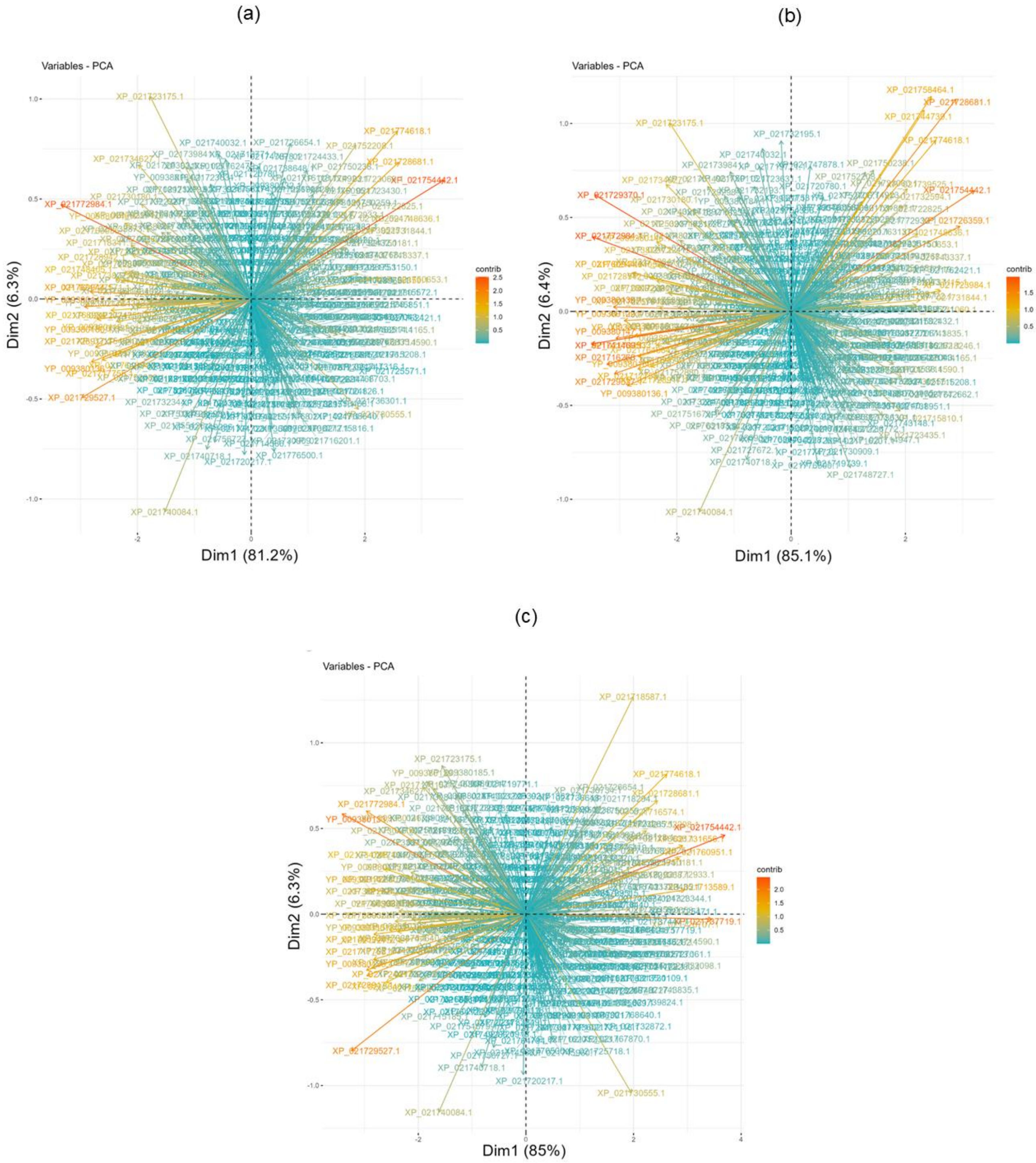

Figure S1-12. Principal component analysis with a loadings plot performed for all the treatment groups (roots, young and mature leaves) with or without stress application (a), all groups of plants grown without Zn treatment (b) and all groups of plants grown under Zn stress conditions (c) (supplementation of 300 µmol/L Zn^{2+} to the hydroponic solution for 7 days).

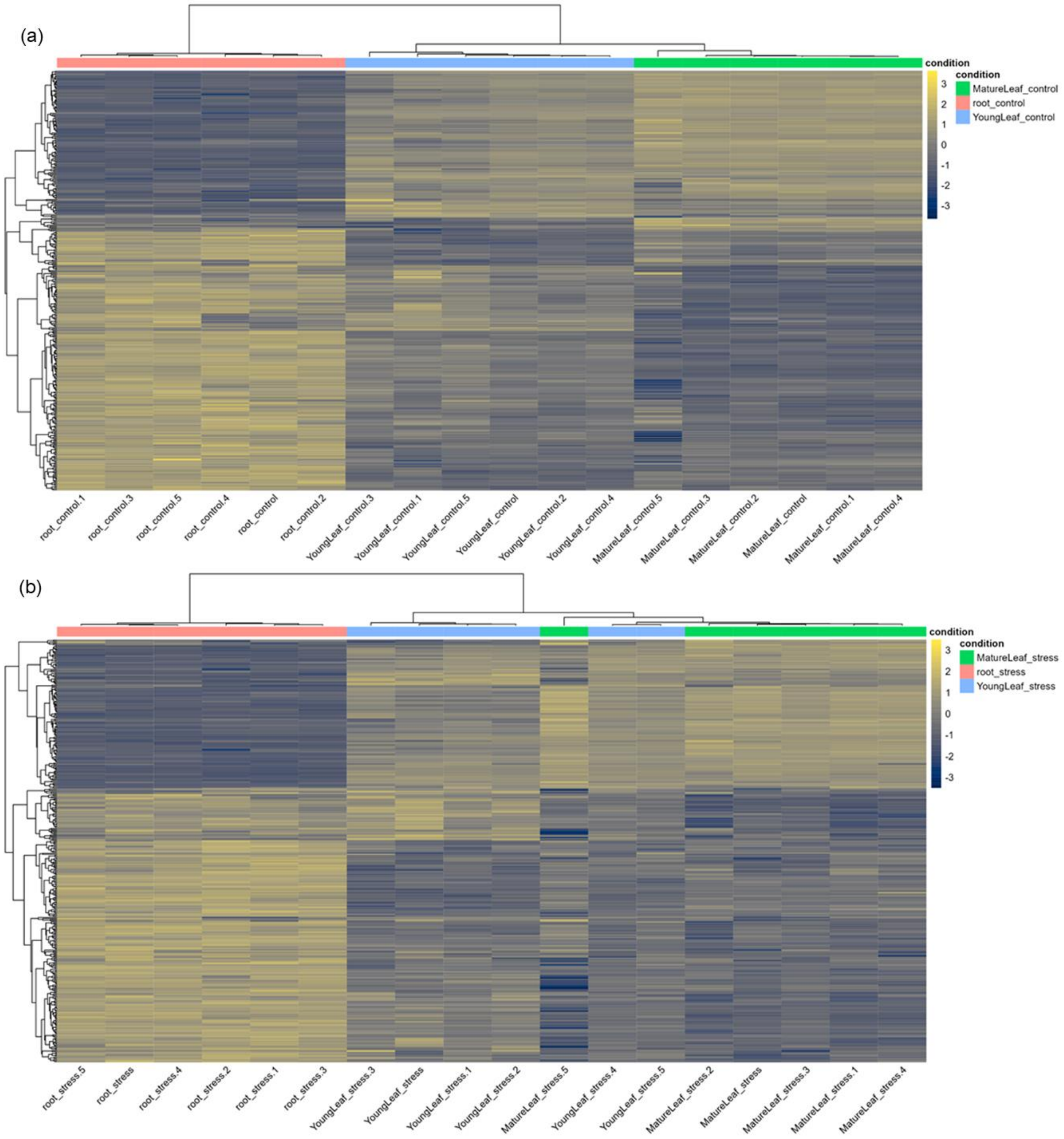

Figure S1-13. Hierarchical clustering with a heatmap representation showing expression patterns of individual samples performed for all groups of plants grown without Zn treatment (a) and all groups of plants grown under Zn stress conditions (b). Control – plants grown without Zn treatment, Stress – plants grown under Zn stress (supplementation of 300 μmol/L Zn^{2+} to the hydroponic solution for 7 days).

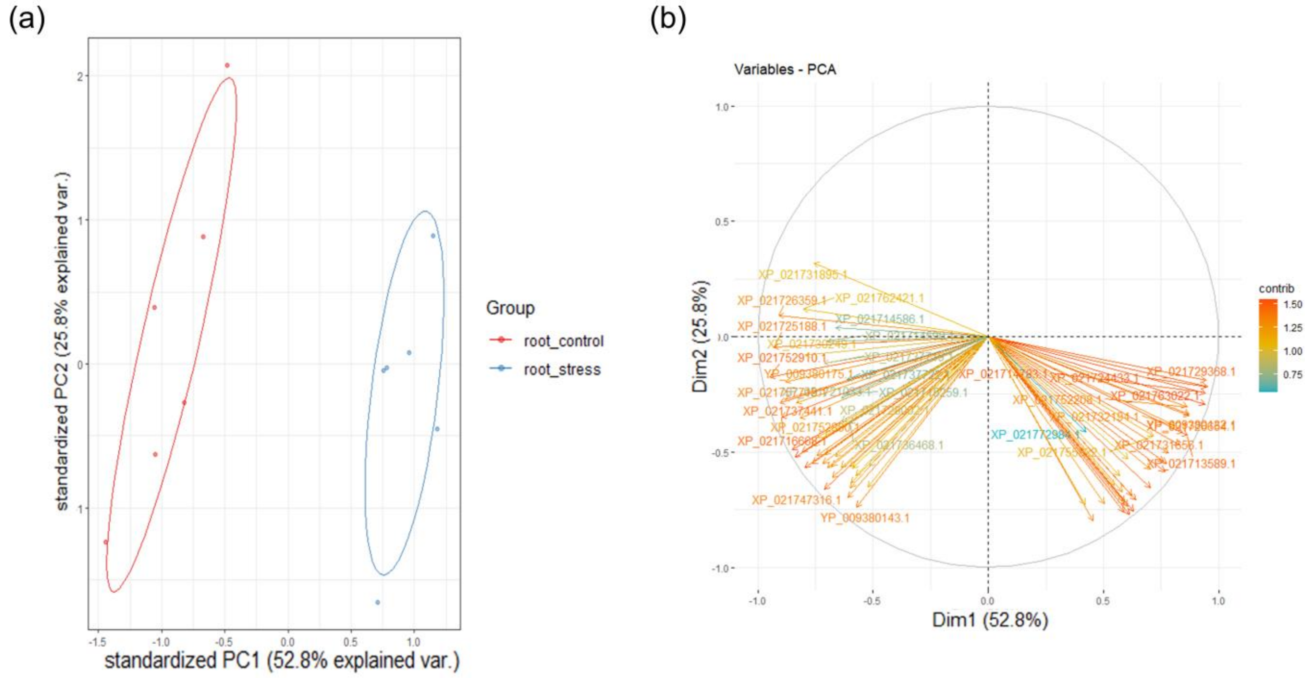

Figure S1-14. Principal component analysis performed for the roots of amaranth plants with a scores plot (a) and loadings plot (b) showing individual contribution of variables in the observed differences. Control – plants grown without Zn treatment, Stress – plants grown under Zn stress (supplementation of 300 µmol/L Zn^{2+} to the hydroponic solution for 7 days).

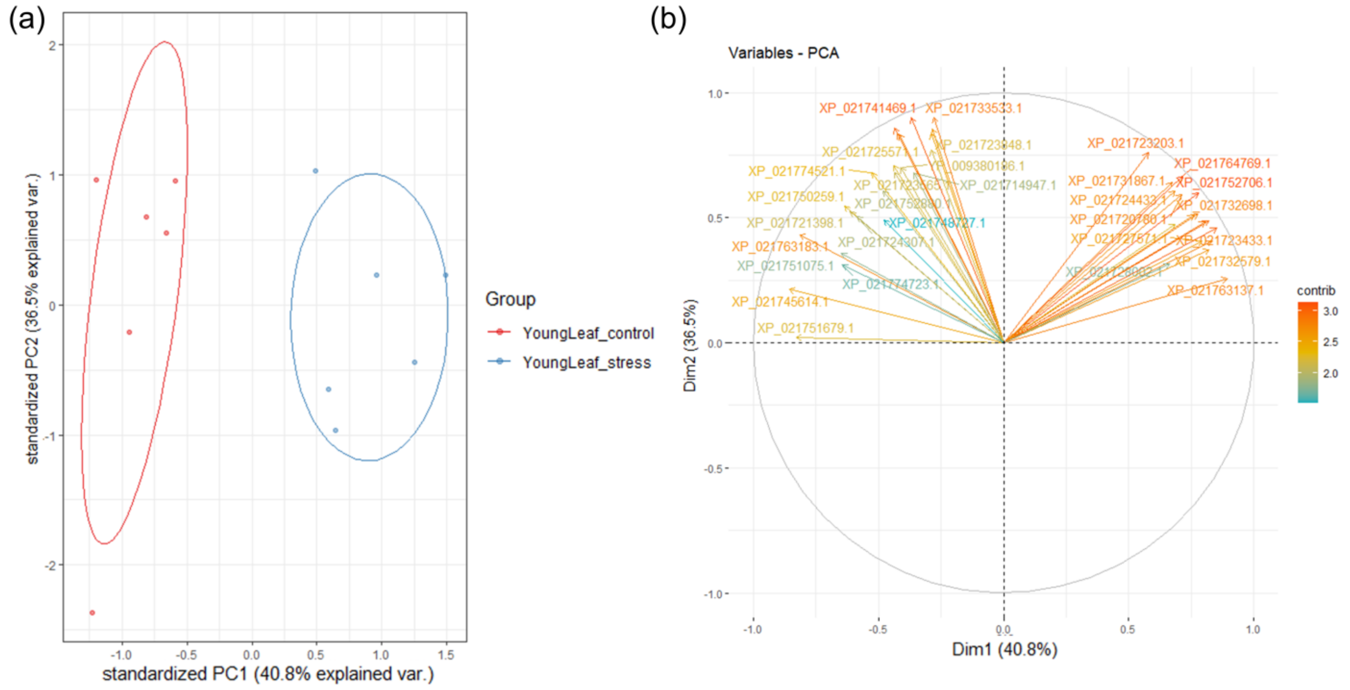

Figure S1-15. Principal component analysis performed for the young leaves of amaranth plants with a scores plot (a) and loadings plot (b) showing individual contribution of variables in the observed differences. Control – plants grown without Zn treatment, Stress – plants grown under Zn stress (supplementation of 300 µmol/L Zn^{2+} to the hydroponic solution for 7 days).

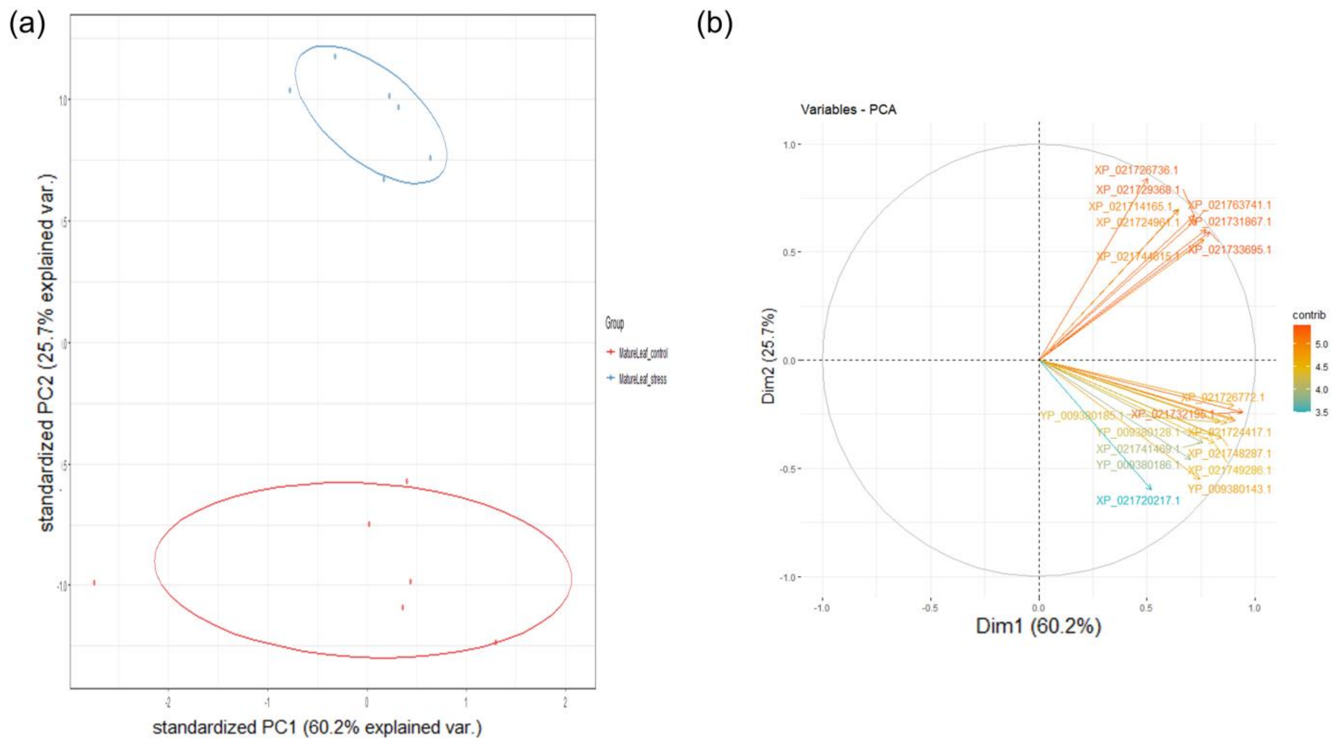

Figure S1-16. Principal component analysis performed for the mature leaves of amaranth plants with a scores plot (a) and loadings plot (b) showing individual contribution of variables in the observed differences. Control – plants grown without Zn treatment, Stress – plants grown under Zn stress (supplementation of 300 μmol/L Zn^{2+} to the hydroponic solution for 7 days).

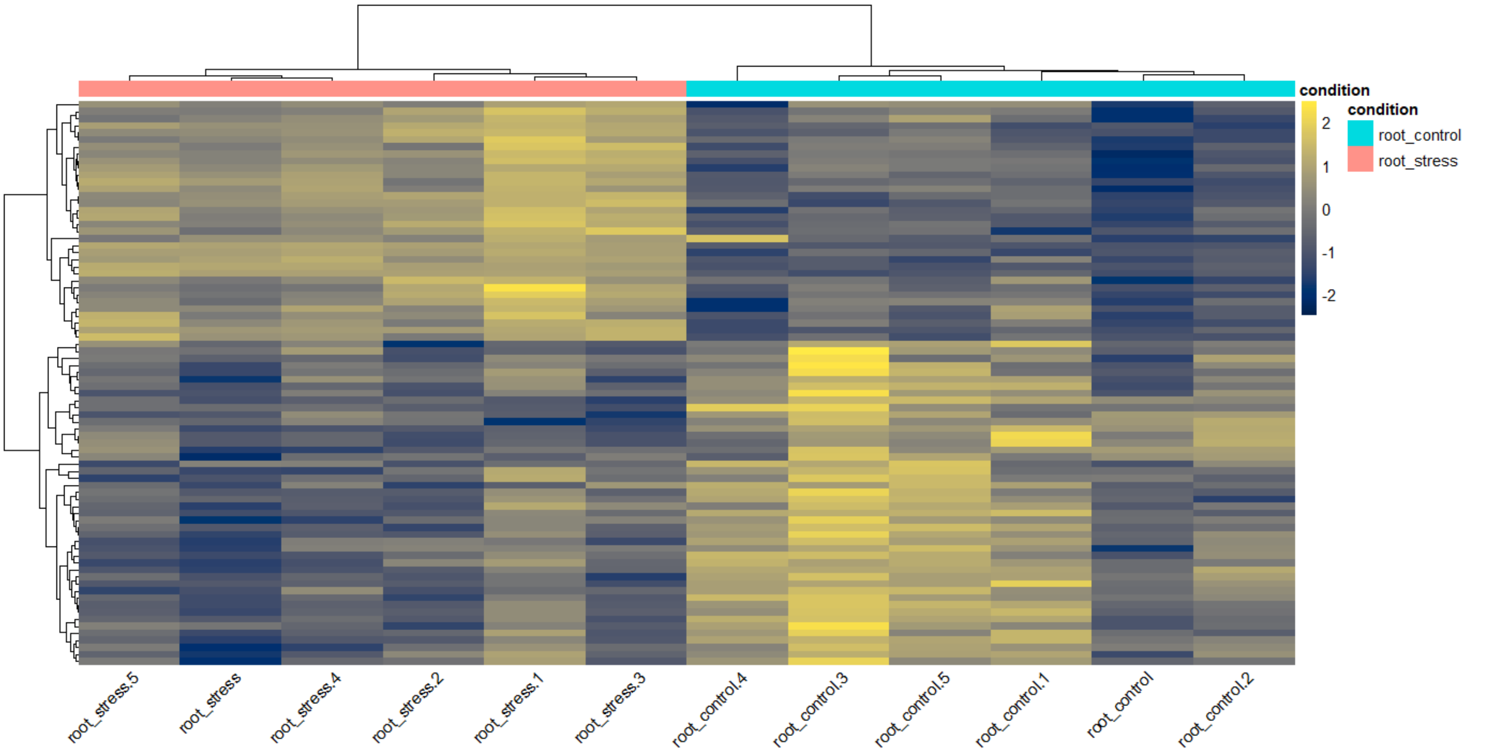

Figure S1-17. Hierarchical clustering with a heatmap representation showing expression patterns in individual samples of the amaranth roots. Control – plants grown without Zn treatment, Stress – plants grown under Zn stress (supplementation of 300 µmol/L Zn^{2+} to the hydroponic solution for 7 days).

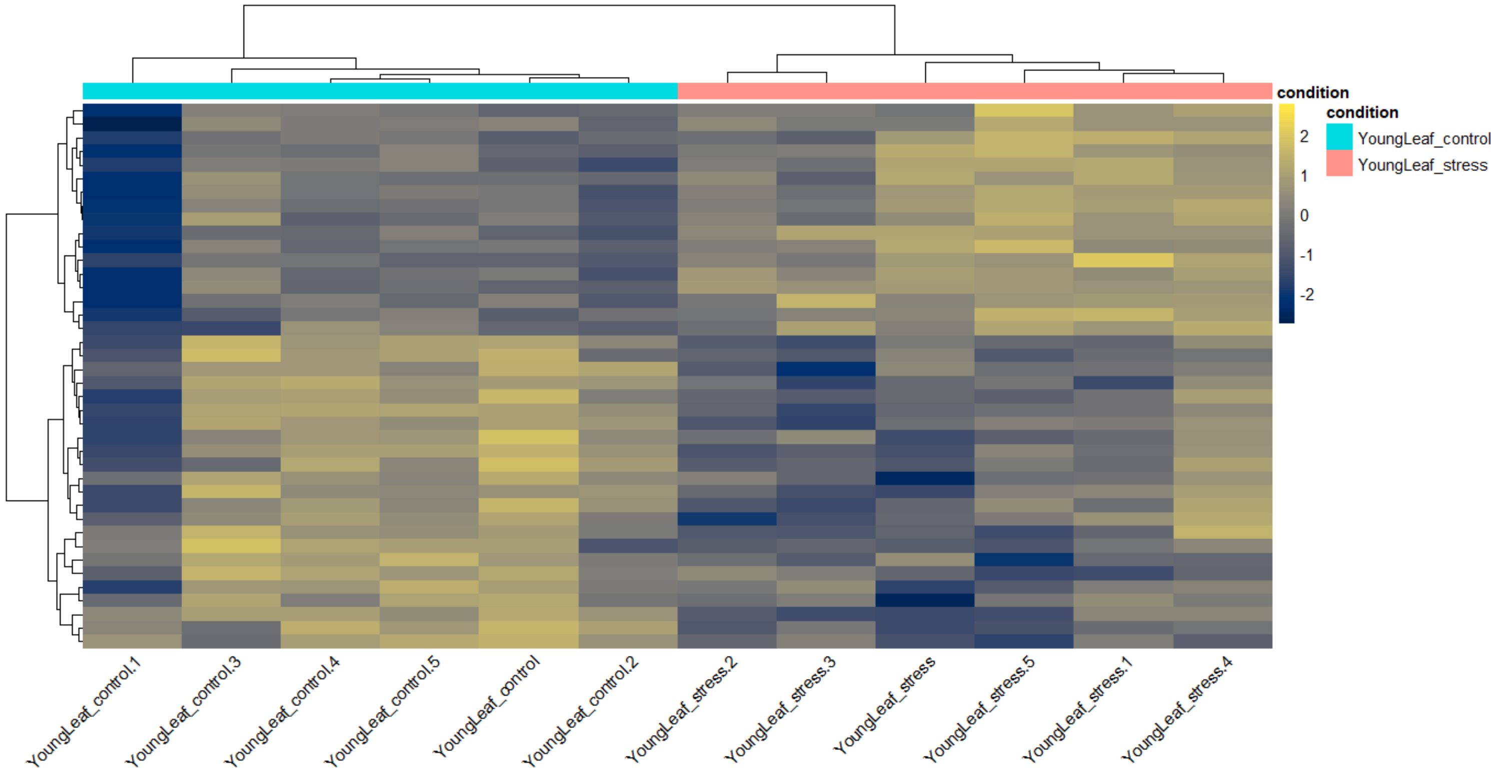

Figure S1-18. Hierarchical clustering with a heatmap representation showing expression patterns in individual samples of the amaranth young leaves. Control – plants grown without Zn treatment, Stress – plants grown under Zn stress (supplementation of 300 µmol/L Zn^{2+} to the hydroponic solution for 7 days).

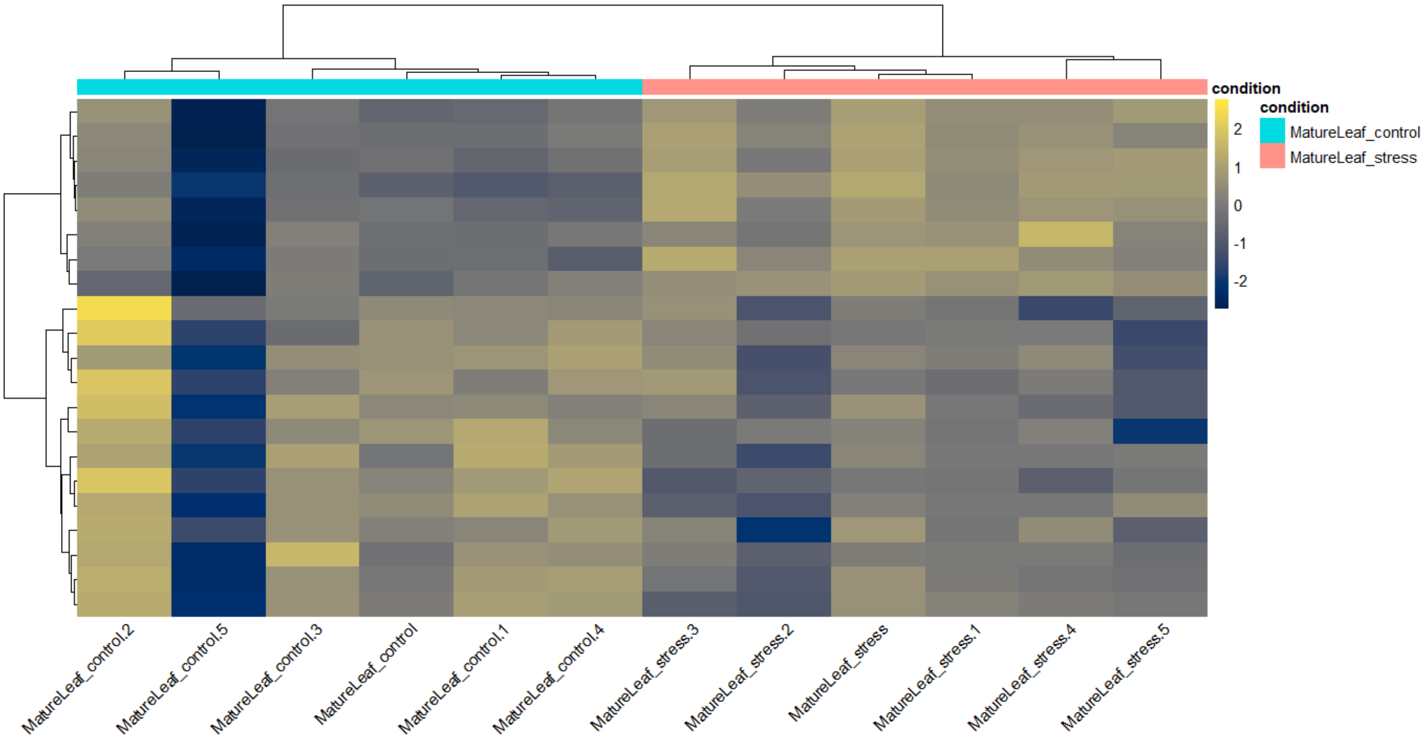

Figure S1-19. Hierarchical clustering with a heatmap representation showing expression patterns in individual samples of the amaranth mature leaves. Control – plants grown without Zn treatment, Stress – plants grown under Zn stress (supplementation of 300 µmol/L Zn^{2+} to the hydroponic solution for 7 days).

Reference list

1. Leonova, T., Ihling, C., Saoud, M., Frolova, N., Rennert, R., Wessjohann, L.A., Frolov, A., 2022. Does filter-aided sample preparation provide sufficient method linearity for quantitative plant shotgun proteomics? Front. Plant Sci. 13, 874761. https://doi.org/10.3389/fpls.2022.874761

2. Osmolovskaya, N.; Bilova, T.; Gurina, A.; Orlova, A.; Vu, V.D.; Sukhikh, S.; Zhilkina, T.; Frolova, N.; Tarakhovskaya, E.; Kamionskaya, A.; et al. Metabolic Responses of *Amaranthus caudatus* Roots and Leaves to Zinc Stress. *Plants* **2025**, *14*, 2119. https://doi.org/10.3390/plants14142119
